# Semidefinite programming for manipulating acoustic traps in real time (SMART)

**DOI:** 10.1038/s41598-025-93153-8

**Published:** 2025-05-20

**Authors:** Sebastian Zehnter, Kevin Endres, Martin Kronbichler, Marco A. B. Andrade, Felix Funke, Christoph Ament

**Affiliations:** 1 Chair of Control Engineering, University of Augsburg, Augsburg, 86159 Germany; 2 Chair of High-Performance Scientific Computing, University of Augsburg, Augsburg, 86159 Germany; 3 Faculty of Mathematics, Ruhr University Bochum, Bochum, 44801 Germany; 4 Institute of Physics, University of São Paulo, São Paulo, 05508-090 Brazil

**Keywords:** Acoustic levitation, Semidefinite programming, Phase recovery, Phase retrieval problem, Acoustic traps, Sound pressure field, Real time, Contactless handling, Block-coordinate minimisation, Burer–Monteiro method, Engineering, Physics

## Abstract

Sound waves can be used for trapping and manipulating objects immersed in liquids or air. However, most acoustic levitation techniques are limited to particles with diameters much smaller than the acoustic wavelength or require time-consuming optimisation-based methods that hinder the dynamic manipulation of objects. Here, we present an approach based on semidefinite programming to manipulate levitated objects in real time. To demonstrate this technique, a phased array consisting of 256 ultrasonic transducers operating at 40 kHz is used for rotating a non-spherical Rayleigh object or to translate Mie spheres along various trajectories. In contrast to previous optimisation-based approaches, the proposed method can determine the emission phases of each transducer in real time, strongly facilitating the implementation of a model-based closed-loop control in future acoustic levitation systems. This is a fundamental step for manipulating levitated objects precisely and at high speeds.

## Introduction

The acoustic radiation force is a non-linear physical phenomenon that results from the interaction of sound waves with the surface of objects^1^. In acoustic levitation, this force is used to counteract the gravitational force and to keep objects in stable suspension^2–4^. In comparison to other contactless handling methods that employ magnetic, electric, optical, or aerodynamic forces^5^, the biggest advantage of acoustic levitation is its ability to trap solid and liquid samples of different material properties^6–12^, sizes^13–17^, and masses^12,14,18^ in multiple media, such as air^14^, water^19^, and biological tissue^20,21^. This feature makes acoustic levitation a highly promising technique in numerous applications, e.g., in photogrammetry^22^, crystallography^23^, spectroscopy^24^, pharmacy^2,25^, and microassembly^26^.

In the past, Langevin-type transducers (LTs) were mostly employed for levitation^27–29^, but in recent studies^15–17,30,31^ they have been replaced by phased arrays of transducers (PATs)^3^. This transition, which gained momentum with the studies of Ochiai et al.^32,33^ and Marzo et al.^14^, is due to the fact that PATs can create more complex sound fields through the individual control of the phase^14^, amplitude^34^, or frequency^35^ of each transducer. In contrast to approaches in acoustic holography that employ passive structures to modulate the phase^36,37^, the spatial resolution of pressure fields generated with PATs is still significantly lower. However, the crucial advantage of PATs is their ability to rapidly change the sound field, which is frequently used to move small and lightweight objects^34,38^. Here, objects are usually enclosed in an acoustic trap, a sound pressure field in which they are stably suspended due to an equilibrium of forces^14^.

In the Rayleigh regime, where particles are much smaller than the acoustic wavelength $\lambda$ (diameter $d \ll \lambda$), the manipulation capabilities of traps were limited to four degrees of freedom (DOF) on a single object^14^, or, in case of several objects, to certain arrangements^39,40^, in the past. In recent years, these restrictions could be mostly resolved by approaches employing singular value decomposition (SVD)^22,41^ for a single object or holographic acoustic elements^14^ and multi-point algorithms^30,34,42^ in case of multiple objects. In the Mie $(d \approx \lambda )$ and the geometric regime $(d \gg \lambda )$, the progress was much smaller. Although the PAT-based static levitation of a regular octahedron with a diagonal length of $5.9\,\lambda$^15^ and of spheres with $\lambda$^16^ or even multiple $\lambda$ in diameter^15,17,43^ have been shown, a controlled dynamic manipulation of such objects has not been demonstrated yet due to many limitations. 

**Fig. 1:**
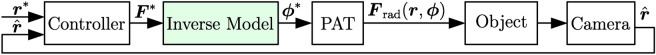
**Sketch of a position control loop**. Based on a desired position $\varvec{r}^{*} \in {\mathbb {R}}^{3 \times 1}$ and a measured position $\hat{\varvec{r}} = \hat{r}_\textrm{x} \varvec{e}_\textrm{x} + \hat{r}_\textrm{y} \varvec{e}_\textrm{y} + \hat{r}_\textrm{z} \varvec{e}_\textrm{z}$, a controller will compute a desired force $\varvec{F}^{*}$. From the input $\varvec{F}^{*}$, an inverse model highlighted in green will determine a feasible control vector, here a vector of phase angles $\varvec{\phi }^{*} \in {\mathbb {R}}^{{N} \times 1}$, to individually control each of the *N* transducers of the PAT. Subsequently, the emitted wave fronts from the transducers will superimpose to an appropriate sound field. This field impinges on the surface of the levitated object, exerting the radiation force $\varvec{F}_\textrm{rad}(\varvec{r},\varvec{\phi })$ that causes the object to move from its current position $\varvec{r}$ to $\hat{\varvec{r}}$. This quantity is obtained by a camera system and fed back to close the control loop.

The key problem here is the lack of a real-time capable inverse model that would enable a model-based closed-loop control, see Fig. 1. Although several linear^44–46^ and non-linear models^47,48^ have been proposed to predict the motion of Rayleigh objects based on a given control vector $\varvec{\phi }$, it was pointed out in the work of Paneva et al.^48^ that it is usually not possible to invert these models due to the lack of differential flatness^49^. This means that the radiation force $\varvec{F}_\textrm{rad}(\varvec{r},\varvec{\phi })$ exerted on an object placed at $\varvec{r}$ can be obtained from a given $\varvec{\phi }$, but the inverse projection is ambiguous, since a feasible $\varvec{\phi ^{*}}$ usually cannot be inferred directly from a desired force $\varvec{F}^{*}.$This problem is also evident in the few proposed approaches^50–52^, where crucial simplifications were exploited for controller design that only apply to their applications. As a consequence, there is currently no generalisable model-based closed-loop control that can be applied to various tasks in contactless handling. However, only such a control allows the compensation of unknown disturbances like acoustic streaming^53,54^, acoustic viscous torques^55^, and harmonic generation^56^, as these physical phenomena are often neglected in acoustic models^26^. Furthermore, its usage would improve current limitations of acoustic levitation systems such as low speed, undesired oscillations of the levitated object and reduced precision in positioning. Thus, a model-based closed-loop control is a mandatory requirement to use PAT-based acoustic levitation systems not only in academic research, as it is the case today, but also reliably in future industrial applications. The keystone to its realisation is a generalisable and real-time capable inverse model, which is currently not available.

Here, we present such a model that allows the manipulation of acoustic traps in real time. The core element of the inverse model is an algorithm that is able to obtain a feasible $\varvec{\phi }^{*}$ for the PAT in real time that shifts a given complex pressure field, sampled at certain control points, by a desired vector $\Delta \varvec{r}^{*}$. Based on the work of Waldspurger et al.^57^, we approximate this phase retrieval problem by transforming it into a convex semidefinite program (SDP)^58^ called *PhaseCut*^57^, which is similar to the well-known *MaxCut*^59^ problem. This allows us to apply recent results for *MaxCut* to *PhaseCut* and to develop a greedy algorithm based on techniques like the Burer–Monteiro method^60,61^ and block-coordinate minimisation (BCM)^62^. As a result, our proposed algorithm provides both high convergence rate and low execution time, solving *PhaseCut* in up to $0.6\,\textrm{ms}$ in our use case ($256\times 26$). Even for a high number of transducers *N* and control points *M* (e.g., $N \times M = 1024 \times 38$, see Table 2), the execution time is less than $11\,\textrm{ms}$ on a single CPU core.

Furthermore, we show that the algorithm is able to largely restore the original radiation force distribution of a trap even for large total displacements, allowing the force distribution to be considered as constant for small trap shifts. This fact has significantly facilitated the creation of two multivariate polynomial models that unambiguously link an approximate resulting force $\varvec{F}_\textrm{res}$ acting on the object to a trap shift by $\Delta \varvec{r}$ (and vice versa). These models as well as the aforementioned algorithm essentially form the inverse model (see Fig. 1) that establishes the causal chain $\varvec{F}^{*} \rightarrow \Delta \varvec{r} \rightarrow \varvec{\phi }^{*}$. Moreover, we demonstrate how the proposed inverse model can be used together with a dynamics model of a levitated object. To achieve this, we apply a model predictive control (MPC) algorithm on a path-following problem to translate a Mie sphere along a given reference trajectory by means of a model-based optimal feed-forward control. This paves the road for the development of a model-based closed-loop control for acoustic levitation systems.

In addition, we demonstrate the real-time capability and the precision of the proposed method in several experiments: In case of a pure kinematic open-loop control, we show the precise rotation of a non-spherical Rayleigh object in a twin trap as well as the translation of a Mie sphere in mid-air along a sinusoidal trajectory (see supplementary video 1). In contrast, the advantages of a model-based optimal feed-forward control are exemplary demonstrated for a linear (see supplementary videos 2 & 3), circular (supplementary video 4), infinite symbol (supplementary video 5), and a cross house trajectory (supplementary video 6). The fast linear trajectory achieved approximate velocities of up to $150\,\textrm{mm}\,\textrm{s}^{-1}$, the circular, infinite symbol and cross house trajectories showed maximum position uncertainties of $e_\textrm{max} \approx 1.0\,\textrm{mm}$, $0.8\,\textrm{mm}$, and $0.6\,\textrm{mm}$ respectively.

## Results and discussion

### Structure of the inverse model

**Fig. 2:**
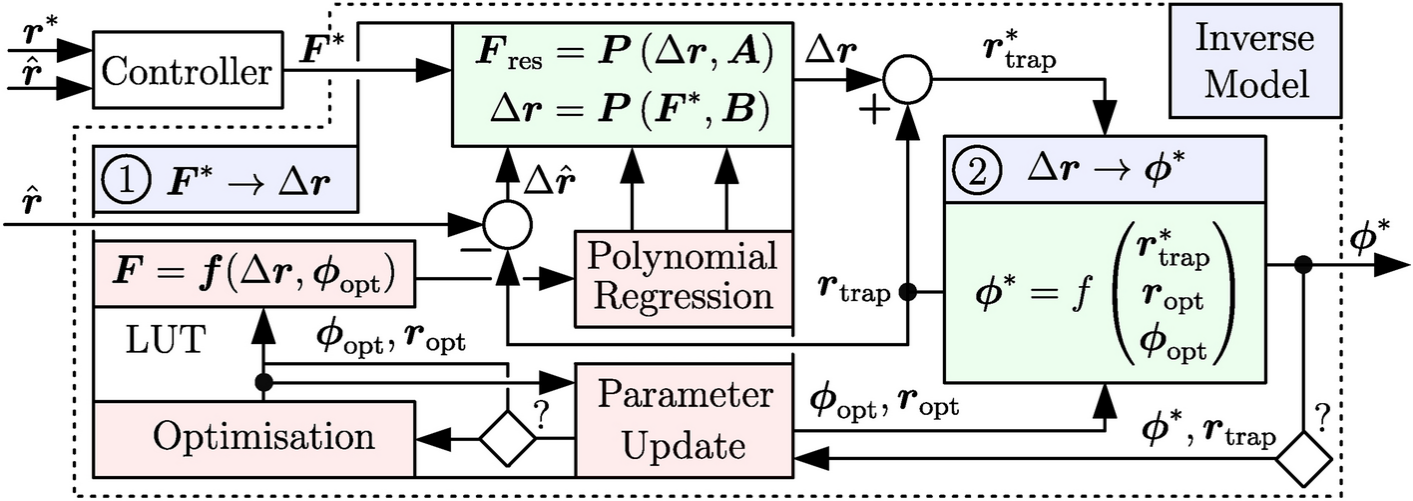
**Structure of the inverse model**. The components ${\textcircled {{1}}}$ and ${\textcircled {{2}}}$ of the inverse model (), the real-time capable functions () iteratively executed in the *online phase*, and the auxiliary functions () executed only a priori in *offline phases* are highlighted.

The structure of the inverse model is depicted in Fig. 2. Its task is to unambiguously determine a feasible activation $\varvec{\phi }^{*}$ for the PAT that exerts a desired force $\varvec{F}^{*}$ on the levitated object. The model is comprised of the components ${\textcircled {{1}}}$ ($\varvec{F}^{*} \rightarrow \Delta \varvec{r})$ and ${\textcircled {{2}}}$ ($\Delta \varvec{r} \rightarrow \varvec{\phi }^{*}$) to set up the necessary causal chain $\varvec{F}^{*} \rightarrow \Delta \varvec{r} \rightarrow \varvec{\phi }^{*}$, which ensures the usability of the inverse model for a closed-loop control, see Fig. 1. The first step of component ${\textcircled {{1}}}$ is to determine an initial activation $\varvec{\phi }_\textrm{opt}$ for the PAT that stably suspends an object at $\varvec{r}_\textrm{opt}$. To obtain $\varvec{\phi }_\textrm{opt}$, most approaches rely on optimisation-based methods that employ accurate, but rather time-consuming force models^15–17^. In consequence, these approaches are not real-time capable, especially for big PATs consisting of 256 – 1024 transducers. This means that a valid update of $\varvec{\phi }$ to change the resulting sound pressure field cannot be calculated in a feasible time span for the control circuit. Consequently, we suggest to employ such methods only a priori in an *offline phase* rather than iteratively execute them inside the closed loop during the *online phase*. After a feasible $\varvec{\phi }_\textrm{opt}$ for a given $\varvec{r}_\textrm{opt}$ has been calculated, we continue the *offline phase* by sampling the acoustic radiation force distribution around the generated trap to create a look-up table (LUT). This LUT is comprised of relative distances $\Delta \varvec{r} = \varvec{r} - \varvec{r}_\textrm{trap}$ of the position $\varvec{r}$ of the levitated object from the centre of the acoustic trap $\varvec{r}_\textrm{trap}$ and the corresponding resulting forces $\varvec{F}_\textrm{res} = \varvec{F}_\textrm{rad}(\Delta \varvec{r},\varvec{\phi }_\textrm{opt}) + \varvec{F}_\textrm{g}$, where $\varvec{F}_\textrm{g} = m \varvec{g}$, *m* is the mass of the levitated object, and $\varvec{g} = g_\textrm{x} \varvec{e}_\textrm{x} + g_\textrm{y} \varvec{e}_\textrm{y} - g_\textrm{z} \varvec{e}_\textrm{z}$ denotes the vector of gravitational acceleration. Depending on the object properties and the desired accuracy of the result, various approaches^15–17,63–65^ can be used to determine $\varvec{F}_\textrm{rad}$. The *offline phase* ends with a polynomial regression (see section Polynomial regression in Methods for details), where, based on the LUT data, the coefficient matrices $\varvec{A} \in {\mathbb {R}}^{\alpha \times 3}$ and $\varvec{B} \in {\mathbb {R}}^{\beta \times 3}$ with $\alpha ,\beta \in {\mathbb {N}}^{+}$ of two polynomial models are determined. These models are used to unambiguously predict a resulting force $\varvec{F}_\textrm{res} = \varvec{P}(\Delta \varvec{r},\varvec{A})$ (used for model-based feed-forward control) acting on the levitated object for a given displacement $\Delta \varvec{r}$ as well as to obtain a feasible $\Delta \varvec{r} = \varvec{P}\left( \varvec{F}^{*},\varvec{B}\right)$ in order to exert a desired force $\varvec{F}^{*}$ on the object. These predictions can be performed in real time, since the computational costs for the evaluation of polynomials are minimal. This approach provides a high prediction accuracy for a big volume $\mathcal{V} = \left\{ \varvec{r}_i \in {\mathbb {R}}^3 \, \big \vert \, \Vert \varvec{r}_i - \varvec{r}_\textrm{opt} \Vert _2 \le \epsilon _\mathcal {{V}} \right\}$ centred around $\varvec{r}_\textrm{opt}$, where $\epsilon _\mathcal {V} \in {\mathbb {R}}^{+}$ is in the range of several millimetres and $\Vert \varvec{r}_i - \varvec{r}_\textrm{opt} \Vert _2$ denotes the Euclidean norm between $\varvec{r}_i$ and $\varvec{r}_\textrm{opt}$. Finally, if the levitated object leaves $\mathcal {V}$ or the total working space $\mathcal {W}$ should be increased by concatenating several volumes $\mathcal {V}_{i}$, it is optionally possible (indicated by the rhomb and the question mark in Fig. 2) to trigger a parameter update, where the functions of the *offline phase* will be successively executed again.

After completing the linkage $\varvec{F}^{*} \rightarrow \Delta \varvec{r}$, we take a look at component ${\textcircled {{2}}}$ of the inverse model in Fig. 2 that establishes the relation $\Delta \varvec{r} \rightarrow \varvec{\phi }^{*}$. Here, the task is to determine a feasible activation $\varvec{\phi }^{*}$ that recreates a given acoustic trap, centred at $\varvec{r}_\textrm{opt}$ and defined by $\varvec{\phi }_\textrm{opt}$, at a desired location $\varvec{r}_\textrm{trap}^{*}$. To achieve this, the simplest way is to keep the activations $\varvec{\phi }_\textrm{opt}$ and move the whole PAT by $\Delta \varvec{r} = \varvec{r}_\textrm{trap}^{*} - \varvec{r}_\textrm{opt}$, which is possible when the PAT is mounted on a robot arm^66,67^. Otherwise, $\varvec{\phi }^{*}$ is only feasible when the original radiation force distribution of the trap is recreated at the new adjacent trap centre $\varvec{r}_\textrm{trap}^{*}$. As existing optimisation-based methods^15–17^ are currently too slow to be iteratively executed in a real-time closed-loop control, we propose a different approach. Since the acoustic radiation force $\varvec{F}_\textrm{rad}$ is directly linked with the complex acoustic pressure $\widetilde{\varvec{p}}$ exerted on the object surface, a match of the radiation force distributions around these two locations can be obtained when the original acoustic pressure field of the trap, given by $\widetilde{\varvec{p}} \in {\mathbb {C}}^{P \times 1}$ at locations $\varvec{r}_{i} \in \mathcal {V}$, can be recreated at positions $\varvec{s}_{i} \in \mathcal {V}$ that result from $\varvec{s}_{i} = \varvec{r}_{i} + \Delta \varvec{r}$, $i \in \left\{ 1,2,\dots ,P\right\}$.

A very popular approach for this task is the use of *holographic acoustic elements*, introduced by Marzo et al.^14^. Here, a given activation $\varvec{\phi }_{0}$ for the PAT that stably suspends an object at $\varvec{r}_{0}$ is split up into a focal lens $\varvec{\phi }_{\textrm{foc},0}$ and a trap signature $\varvec{\phi }_{\textrm{trap},0} = \varvec{\phi }_0 - \varvec{\phi }_{\textrm{foc},0}$, where the components $\phi _{\textrm{foc},{0,j}}$ with $j \in \left\{ 1,2,\dots ,N\right\}$ of $\varvec{\phi }_{\textrm{foc},0} \in {\mathbb {R}}^{N \times 1}$ are given by $\phi _{\textrm{foc},{0,j}} = - k \Vert \varvec{r}_0 - \varvec{r}_{\textrm{t},j} \Vert _2$, denoting by $\varvec{r}_{\textrm{t},j}$ the position of the *j*-th transducer of the PAT and by *k* the wave number. Next, a feasible activation $\varvec{\phi }_1 \in {\mathbb {R}}^{N \times 1}$ to shift the trap from $\varvec{r}_{0}$ to $\varvec{r}_1$ is obtained by refocusing the acoustic beam with the approximation $\varvec{\phi }_1 \approx \varvec{\phi }_{\textrm{trap},0} + \varvec{\phi }_{\textrm{foc},1}$. Since the computational costs of these two operations are minimal, the real-time capability of this approach is certainly ensured. Consequently, this method is frequently employed to establish a simple kinematic open-loop control. This is done by iteratively switching activations $\mathcal {A} = \left\{ \varvec{\phi }_0, \varvec{\phi }_1, \varvec{\phi }_2, \dots , \varvec{\phi }_n \right\}$ in ascending order at specific times $t_i$ in order to move an object safely from $\varvec{r}_{0}$ to $\varvec{r}_{n}$ along a discretised trajectory $\mathcal {T} = \left\{ \varvec{r}_{0}, \varvec{r}_{1} , \varvec{r}_{2}, \dots , \varvec{r}_{n} \right\}$ at a moderate velocity, ensuring a sufficiently small distance $\Delta {r}_{ij} = \Vert \varvec{r}_{j} - \varvec{r}_{i}\Vert _2$ between two adjacent positions $\varvec{r}_{i}$ and $\varvec{r}_{j}$, e.g., $\Delta {r}_{ij} \le 0.2\,\textrm{mm}$ for small particles^68^. However, despite its simplicity and popularity, this approach has a significant drawback. Looking at the widely applied piston source model (see Eq. (7) in the work of Marzo et al.^14^), it becomes clear that this method only approximates the phases of the total complex pressure at positions adjacent to the new trap centre, but does not take amplitude changes into account that are caused by the geometric refocusing of the beam. Consequently, this method can only be applied in a small working space $\mathcal {W} = \left\{ \varvec{r}_i \in {\mathbb {R}}^3 \, \big \vert \, \Vert \varvec{r}_i - \varvec{r}_\textrm{opt} \Vert _2 \le \epsilon _\mathcal {W} \right\},$where $\epsilon _\mathcal {W} \in {\mathbb {R}}^{+}$ is in the range of a few millimetres. If the total displacement from the original trap centre $\varvec{r}_\textrm{opt}$ exceeds $\epsilon _\mathcal {W}$, the accuracy of the algorithm, defined by the Euclidean distance between the new trap centre and the equilibrium position $\varvec{r}_\textrm{eq}$ of the object inside the trap, decreases considerably (see Fig. 6), which is caused by a strong mismatch of the radiation force distribution of the shifted trap.

**Fig. 3:**
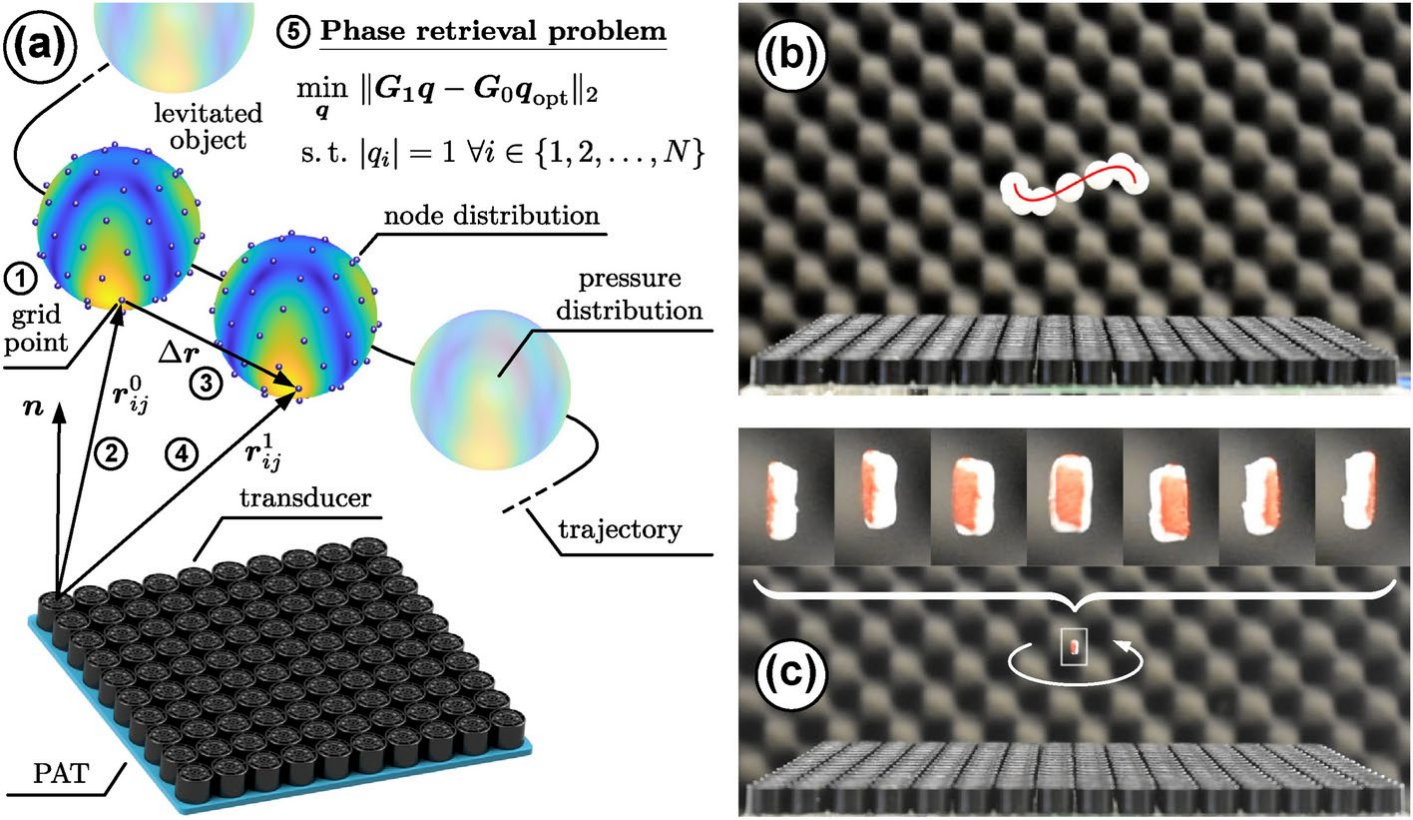
**(a)** Conceptual idea of the trap shift algorithm (${\textcircled {{2}}}$ $\Delta \varvec{r} \rightarrow \varvec{\phi }^{*}$ in Fig. 2). To move a trap given by $\varvec{q}_\textrm{opt} = e^{\textrm{i}\varvec{\phi }_\textrm{opt}} \in {\mathbb {C}}^{N \times 1}$ from $\varvec{r}_{\mathcal {T},0}$ to $\varvec{r}_{\mathcal {T},1} = \varvec{r}_{\mathcal {T},0} + \Delta \varvec{r}$, the following steps according to Algorithm 3 are conducted: ${\textcircled {{1}}}$ Sample the pressure field, specified by $\widetilde{\varvec{p}} \in {\mathbb {C}}^{P \times 1}$ at locations $\mathcal {P} = \left\{ \varvec{r}_1, \varvec{r}_2, \dots , \varvec{r}_P \right\}$, at nodes $\mathcal {M} = \left\{ \varvec{r}_1, \varvec{r}_2, \dots , \varvec{r}_M \right\}$, $M \ll P$, and calculate the propagator matrix $\varvec{G}_0 \in {\mathbb {C}}^{M \times N}$ (${\textcircled {{2}}}$) for a PAT consisting of *N* transducers. Then shift $\mathcal {M}$ by $\Delta \varvec{r}$ (${\textcircled {{3}}}$) and repeat ${\textcircled {{1}}}$ to obtain $\varvec{G}_1 \in {\mathbb {C}}^{M \times N}$ (${\textcircled {{4}}}$). Finally, solve the phase retrieval problem (${\textcircled {{5}}}$) to get a feasible $\varvec{q}^{*} = e^{\textrm{i}\varvec{\phi }^{*}}$ for $\varvec{r}_1$. **(b)** Translation of an expanded polystyrene (EPS) sphere of $8.1\,\textrm{mm}$ in diameter. **(c)** Controlled rotation of an EPS particle. Supplementary movie 1 shows the translation of the Mie sphere and the rotation of an EPS particle.

In consequence, the method of *holographic acoustic elements*^14^ cannot be applied to our problem and we instead opt to formulate a complex-valued phase retrieval problem. To quickly obtain a feasible $\varvec{\phi }^{*}$, we relax this problem by approximating it with a semidefinite program (SDP) called *PhaseCut*^57^, for which we propose a real-time capable algorithm that is stated in Algorithm 3 (see section Semidefinite programming in Methods for details). Its conceptual usage to realise a simple kinematic open-loop control is illustrated in Fig. 3(a). As it can be seen further from Figs. 3(b) and 3(c) as well as from the corresponding supplementary video 1, it is indeed possible to translate and rotate both spherical and non-spherical objects in the Rayleigh as well as in the Mie regime in real time by shifting the sampled sound pressure field distribution of a given acoustic trap by means of Algorithm 3.

### Phase retrieval problem and semidefinite programming

The phase retrieval problem has many undesired properties such as non-convexity, numerous local minima, and the huge number of quadratic constraints that make it very difficult to obtain a valid solution in a feasible time span. Starting with the Gerchberg–Saxton algorithm in 1972^69^, numerous methods such as Eigensolver^42^, IASA^36,70^, IBP^30^, GS–PAT^34^, Diff–PAT^71,72^, LSS^73^, and approaches based on a brute-force search^74,75^, the L-BFGS-B^76^ or the Levenberg–Marquardt (LM) algorithm^77–79^ have been presented to solve this problem. In addition, recently proposed data-driven solutions^80–82^ based on artificial neural networks (ANN) are becoming increasingly important. A detailed overview of current developments can be found in the work of Yang et al.^83^.

However, all the stated methods are of limited use for the present application. For instance, IASA gives accurate results, but usually requires too much computation time, whereas GS–PAT converges quickly to a result, but its accuracy decreases for an increasing number of control points $M \ge 8$^34^. In addition, ANN-based methods are certainly real-time capable, but have a major weakness due to their lack of adaptability. For example, if the transducer amplitudes are altered or the environmental conditions such as temperature, humidity or air pressure change, the only way to deal with this problem is to generate new data points and train the ANN again, which is usually a time-consuming process, depending on the size and layers of the ANN. Finally, some of these methods, e.g. Chen et al.^73^ and Suzuki et al. ^74^, have been exclusively developed for haptic applications, as they only take the amplitudes, but not the phases of $\widetilde{\varvec{p}}$ into account^74^. However, the phases of $\widetilde{\varvec{p}}$ have a non-negligible influence on the resulting acoustic radiation force distribution of the trap, see for example the methods section in the work of Marzo et al.^14^.

To enable the real-time shifting of traps with high accuracy, we employ the idea of Waldspurger et al.^57^, who proposed to approximate the phase retrieval problem by a tractable relaxation into a SDP^58^ called *PhaseCut*, see also^84^. This problem is very similar to the *MaxCut* problem^62^, although there are two notable differences: First, *MaxCut* is a maximisation problem, whereas in *PhaseCut* Eq. (13) is minimised to find an optimal $\varvec{Z}^{*}.$Second, $\varvec{W}$ and $\varvec{Z}$ are real matrices in *MaxCut*^62^, whereas *PhaseCut* is an optimisation problem over the unit complex torus^57^. These observations imply that algorithms developed for *MaxCut* can very likely be adapted to solve *PhaseCut*. Since *MaxCut* is an extensively studied problem, there are numerous published methods for solving it, e.g., refs.^57,59,62,73,84–87^. Our investigations lead to the conclusion that among these a block coordinate minimisation (BCM) with a low-rank factorisation $\varvec{Z} = \varvec{V}^\textrm{H} \varvec{V}, \varvec{Z} \in {\mathbb {C}}^{(N+1) \times (N+1)}$, called the Burer–Monteiro method^60,61^, is particularly suitable for solving Eq. (13). Thus, we mainly adapt the results from refs.^59,62,86^ to *PhaseCut*, which lead to the development of Algorithm 1. Its speed and the accuracy of recreated sound pressure field is affected by three selectable parameters: The number of rows *L* ($1 \le L \ll (N+1)$) of the low-rank matrix $\varvec{V} \in {\mathbb {C}}^{L \times (N+1)}$, the maximum number of iterations $i_\textrm{max}$, as well as the number and placement of nodes $\varvec{r}_i$ with $i = 1,2,\dots ,M$ and $M \ll P$ at which the original field $\widetilde{\varvec{p}} \in {\mathbb {C}}^{P \times 1}$ is sampled to obtain $\varvec{p} \in {\mathbb {C}}^{M \times 1}$.

Without limiting the general case, we investigated the influence of these parameters on Algorithm 1 (respectively Algorithm 3) in simulation by an exemplary shift of a *twin tuning forks trap*^16^ from $\varvec{r}_\textrm{opt} = (0\,\varvec{e}_\textrm{x} + 0\,\varvec{e}_\textrm{y} + 45\,\varvec{e}_\textrm{z})\,\textrm{mm}$ to the position $\varvec{r}_1 = (0\,\varvec{e}_\textrm{x} + 0\,\varvec{e}_\textrm{y} + 50\,\varvec{e}_\textrm{z})\,\textrm{mm}$, see also Fig. 5. To quantify the quality of the optimisation result, we employed the position error $e_\textrm{pos} = \left\| \varvec{r}_{\textrm{eq},1} - \varvec{r}_1 \right\| _2$ between the new trap centre $\varvec{r}_1$ and the equilibrium position of the object inside the trap $\varvec{r}_{\textrm{eq},1}$. To determine $\varvec{r}_{\textrm{eq},1}$, we initially placed the Mie sphere at $\varvec{r}_1$ in simulation and executed Algorithm 2 with $\varvec{r}_\textrm{eq} = \varvec{r}_1$, $\alpha = 20 \times 10^{-3}$, $F_\textrm{tol} = 1 \times 10^{-19}\,\textrm{N}$, $r_\text{tol} = 5\,\text{mm}$, and $i_\textrm{max} = 4000$ as initial values, see section Characterising sound pressure fields in Methods for details. For this example, we observe a very good result as soon as the objective function $\mathcal {J} = \textrm{tr} \left( \varvec{W} \varvec{V}^\textrm{H} \varvec{V} \right)$ of Algorithm 1 reaches a value of less than approximately $4 \times 10^{3}$, which corresponds to an error of $e_\textrm{pos} \approx 0.125\,\textrm{mm}$, see also Figs. 5 and 6(e). Furthermore, we noticed that the randomised initialisation of the matrix $\varvec{V} \in {\mathbb {C}}^{L \times (N+1)}$ for different values of *L* only has an impact on $\mathcal {J}$ at the start of the optimisation, but this influence decreases rapidly with an increasing number of iterations. Since this observation held true in further investigations on different trap types and displacements, we conclude that the parameter *L* neither has a quantifiable influence on the required number of iterations nor on the quality of the optimisation result. Thus, it is reasonable to use $L = 1$ in Algorithm 1 to improve its execution speed.

In addition to *L*, the quantity and locations of nodes in the grid $\mathcal {M}$ at which the pressure field of the trap is sampled can be adjusted in Algorithm 1. Thus, during the reconstruction of a field defined by $\widetilde{\varvec{p}} \in {\mathbb {C}}^{P \times 1}$ at locations $\mathcal {P} = \left\{ \varvec{r}_1, \varvec{r}_2, \dots , \varvec{r}_P \right\}$, one is faced with the task of choosing a set $\mathcal {M} = \left\{ \varvec{r}_1, \varvec{r}_2, \dots , \varvec{r}_M \right\} \subset \mathcal {P}$ with $M \ll P.$ Such a set $\mathcal {M}$ is feasible when three conditions are fulfilled. First, a valid activation $\varvec{q}^{*} \in {\mathbb {C}}^{N \times 1}$ for the PAT can be obtained from Eq. (8). Second, $\varvec{q}^{*}$ creates $\varvec{p} \in {\mathbb {C}}^{M \times 1}$ at $\varvec{r}_i \in \mathcal {M}$ and third, $\varvec{q}^{*}$ recreates $\varvec{p}$ at $\varvec{r}_j \in \mathcal {P} \setminus \mathcal {M}$ sufficiently to preserve the proper characteristics of the field. This problem can be interpreted in the sense that the cumulative error $e_\Omega$ defined as $e_\Omega = \int _{\Omega } \Vert \varvec{G}\varvec{q} - \widetilde{\varvec{p}} \Vert _{2} \, \textrm{d}\Omega$ over a given surface $\Omega$ should be minimised.

**Fig. 4:**
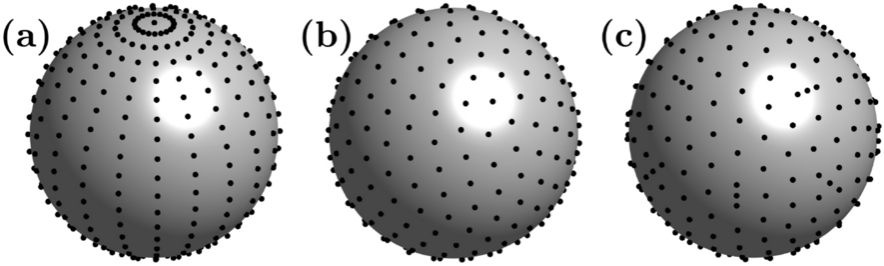
**Examples of node distributions on a spherical surface**. Following the terminology in ref.^88^, a Gaussian grid with $M = 441$ **(a)**, a spherical *t* - design^89^ with $M = 256$ **(b)**, and a Lebedev grid^90,91^ with $M = 266$ **(c)** are depicted.

A complicating aspect here is that this integral cannot be evaluated over the whole surface $\Omega$ due to computational costs. Thus, a few locations $\varvec{r}_i \in \mathcal {M} \subset \Omega$ have to be selected instead, for which a feasible $\varvec{q}^{*}$ exists that reduces $e_\Omega$ to a sufficiently low value. To the best of our knowledge, this problem has not been discussed in literature so far. In our considered examples (see Figs. 3(b) and (c) as well as Figs. 6-9), especially those $\varvec{r}_i$ are suitable that are used as node points in common quadrature formulas. As a consequence, we suggest to use a point grid specified by a Gaussian interpolation scheme^92^ to sample and reconstruct a 2D rectangular acoustic pressure field. In the 3D case, we tested a spherical *t* - design^88^, a Gaussian^88^, and a Lebedev grid^88,90^, which are all depicted in Fig. 4. For their construction, we employed three toolboxes^89,91,93^ in MATLAB. The focus on spherical meshes has two reasons: First, the most prevalent objects in recent studies^16,17,30,34^ have been expanded polystyrene (EPS) spheres. In this case, the mesh could be directly applied to the object surface. Second, this grid type can be used to sample the edge of a volume that contains a desired acoustic pressure distribution that shall be reconstructed, e.g., an acoustic trap. Although it would be desirable to investigate other node distributions to be applied to non-spherical object surfaces, e.g., an octahedron^15^, a systematic investigation of such grid types is beyond the scope of this study. For our two examined cases, namely a *twin tuning forks trap*^16^ (see Fig. 3(b) and Figs. 6 – 9) and a *twin trap*^14^ (see Fig. 3(c)), only $M = 26$ nodes were sufficient to translate or rotate the corresponding sound pressure fields at high precision. Without further evidence, it was even possible to sample the pressure field at only $M = 14$ positions if a slightly lower accuracy (see Algorithm 2) of the shifted trap is acceptable and the total runtime of Algorithm 3 is of main concern. For low node numbers, we have achieved the best results with a Lebedev grid, followed by the spherical *t* - design. With an increasing number of points, the differences between all three grid types became smaller and they achieved a similar performance. In addition, a high number of nodes, e.g., $M \ge 100$, did not significantly improve the positioning accuracy of the shifted trap. For a more detailed analysis of the accuracy of the recreated sound pressure fields, please refer to section [Sec Sec6].

**Fig. 5:**
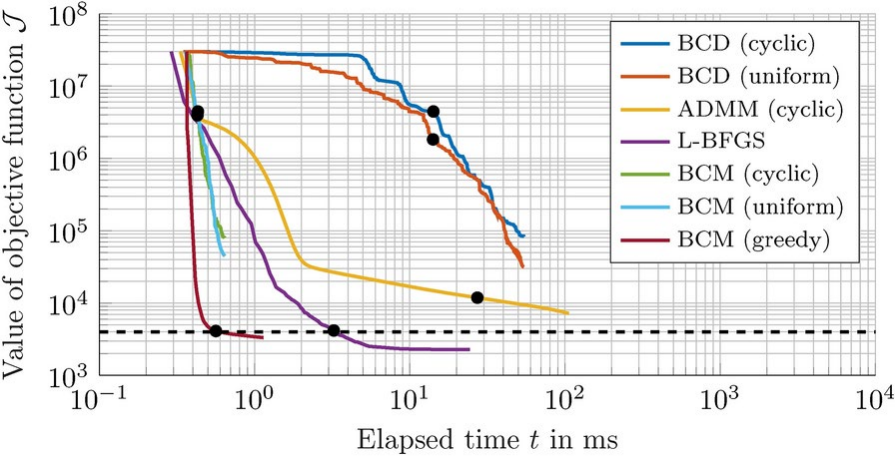
**Convergence speed of various algorithms for the phase retrieval problem**. In this simulative analysis, we employed the parameters of the experimental setup in São Paulo. At first, we obtained a feasible activation $\varvec{\phi }_\textrm{opt}$ from an optimisation^16,17^ to suspend an EPS sphere with $8.1\,\textrm{mm}$ in diameter and a density of $\rho = 15\,\textrm{kg}\mathrm {m^{-3}}$ at $\varvec{r}_\textrm{opt} = (0\,\varvec{e}_\textrm{x}+ 0\,\varvec{e}_\textrm{y} + 45\,\varvec{e}_\textrm{z})\,\textrm{mm}$, $45\,\textrm{mm}$ above the PAT centre, see Fig. 3(b). The phase retrieval problem of a trap shift from $\varvec{r}_\textrm{opt}$ to $\varvec{r}_\textrm{trap} = (0\,\varvec{e}_\textrm{x} + 0\,\varvec{e}_\textrm{y} + 50\,\varvec{e}_\textrm{z})\,\textrm{mm}$ was used. To obtain $\varvec{p} \in {\mathbb {C}}^{M \times 1}$, we sampled $\widetilde{\varvec{p}} \in {\mathbb {C}}^{P \times 1}$ at $M = 26$ nodes that were placed on the object surface using a Lebedev grid scheme^90,91^, see also Fig. 4(c). To ensure a fair comparison, all algorithms (BCD^57,84^, ADMM^73^, L-BFGS^76,94^, and Algorithm 1) with various coordinate selection strategies (see Eq. (22)) were implemented in C++ with a hardware-aware programming style, compiled with the gcc compiler version 13.2 with flags -O3-march=znver4 and run on a *single core* of an AMD Ryzen 7840U CPU operated at 5.1 GHz. Here, we used $i_\textrm{max} = 400$ for L-BFGS as well as $L = 1$ and $i_\textrm{max} = 1000$ for the SDP-based algorithms. For this experiment, an optimal result is reached when the objective function $\mathcal {J}$ yields values of $\lesssim 4 \times 10^{3}$, indicated by the dashed line. Algorithm 1 approximately reaches this value after 256 iterations in $0.56\,\textrm{ms}$, L-BFGS after 50 iterations in $3.0\,\textrm{ms}$. These two points and the values of $\mathcal {J}$ after 256 iterations for the other algorithms are marked in black.

Using the results from these preliminary investigations we initialised $\varvec{V}$ with $$\varvec{V} = \begin{pmatrix} (e^{i\varvec{\phi }_\textrm{opt}})^\top&1 \end{pmatrix}$$ and selected $M = 26$ and $i_\textrm{max} = 1000$ for the runtime analysis depicted in Fig. 5. In this example, Algorithm 1 is outperforming its competitors with a *greedy* coordinate selection strategy. The necessary time of $t_\textrm{opt} \approx 0.56\,\textrm{ms}$ of Algorithm 1 to obtain an optimal solution is, for example, more than five times better that the approach presented in ref.^76^ when an L-BFGS algorithm^94^ is employed. Furthermore, the proposed approach by Chen and Goulart^73^ that uses the alternating direction method of multipliers (ADMM) in combination with the Burer–Monteiro method^60,61^, shows less optimisation progress for the same number of iterations while requiring considerably more computation time. Furthermore, for small trap shifts, as considered here, Algorithm 1 with a *greedy* selection strategy (see section Semidefinite programming in Methods for details) only needs approximately as much iterations as transducers *N* used in the PAT to converge to an optimal result. We presume that this is caused by the fact that in this case some phase angles of the vector $\varvec{\phi }_\textrm{opt}$, especially those of transducers located at the edge of the PAT, do not necessarily have to be adjusted, as their contribution to the sound pressure field for a trap shift by $\Delta r_\textrm{z} = +5\,\textrm{mm}$ barely changes. This observation was also persistent in further investigations with bigger PATs or different object sizes, as long as small translations were maintained.

**Table 1:** Number of iterations, mean run time per iteration, and the resulting total time of the algorithms L-BFGS^76^, ADMM^95^, BCD^57,84^, and Algorithm 1 for the example in Fig. 5.

Method	L-BFGS	ADMM	BCD		Algorithm 1		
Strategy			uniform	cyclic	uniform	cyclic	greedy
iterations	50	256	256	256	256	256	256
time/iter.	$53\,\mathrm {\mu s}$	$109\,\mathrm {\mu s}$	$5.0\,\mathrm {\mu s}$	$5.4\,\mathrm {\mu s}$	$0.3\,\mathrm {\mu s}$	$0.3\,\mathrm {\mu s}$	$0.8\,\mathrm {\mu s}$
total time	${\underline{{3.0}\,\mathrm {{ms}}}}$	${28.2}\,\mathrm {{ms}}$	${14.2}\,\mathrm {{ms}}$	${14.3}\,\mathrm {{ms}}$	${0.50}\,\mathrm {{ms}}$	${0.49}\,\mathrm {{ms}}$	${\underline{{0.56}\,\mathrm {{ms}}}}$

However, for larger trap shifts, Algorithm 1 with a *greedy* selection strategy can take between 2*N* and 4*N* iterations to converge to an accurate result. This decrease in convergence speed and the fluctuations in the required number of iterations were also partially observed when using the L-BFGS algorithm. Further research is needed to provide a thorough explanation for these observations which is, however, beyond the scope of this work. In addition, Fig. 5 shows that algorithms like block-coordinate descent (BCD)^57,84^ or Algorithm 1 with a *cyclic* or *uniform* selection strategy are clearly inferior to Algorithm 1 with a *greedy* strategy, since the former algorithms are not able to converge to an optimal result within $i_\textrm{max} = 1000$ iterations. The low computational cost of Algorithm 1 has a simple reason: For instance, ADMM involves a matrix-vector product in each iteration, leading to a complexity of at least ${\mathcal {O}}(N^2)$ in the number of transducers *N*. For the second-best method, the L-BFGS method, the complexity is at least ${\mathcal {O}}(N\,M)$ per iteration, where *M* denotes the number of control points. Our method, however, only requires a single matrix-vector product at its start-up, followed by updates of vectors from a single column in the coefficient matrix (see Algorithm 1 and the section Real-time capability in Methods for details). In case of $L = 1$, this leads to a reduced complexity of only $\mathcal O(N)$ operations per iteration. Since the identification of the selected row can be performed in ${\mathcal {O}}(1)$ (uniform, cyclic) or ${\mathcal {O}}(N)$ (greedy) steps, the complexity per iteration is only ${\mathcal {O}}(N)$ for the case of $L=1$. This is illustrated by Table 1, in which the number of iterations, the mean run time per iteration, and the resulting total time of all algorithms for the corresponding example in Fig. 5 are listed. Here the total time includes the computation of the involved matrices from $\Delta r$, which requires initial phase computations through Bessel functions (all methods) and a matrix-matrix multiplication (BCD and Algorithm 1), accounting for a significant share of the overall run time (see also Fig. 5). Additionally, the complexity analysis above shows that the proposed methods become even more favorable for higher *N*, potentially outperforming previous methods more significantly.

### Real-time capability

To verify the real-time capability of the proposed method, Table 2 shows the runtime breakdown of an optimised implementation of Algorithm 3 in C++. Here, the computation of ${\varvec{W}} = {\varvec{A}}^\textrm{H} \varvec{A}$ (lines 4–7 in Algorithm 3) takes a larger share of run time than Algorithm 1 itself for many configurations, especially with 26 or 38 control points. Moreover, all cases were completed in less than approx. $11\,\textrm{ms}$, often below $10\,\textrm{ms}$, which can be considered real-time capable. To achieve this, two optimisations are necessary: First, the computation of the Bessel function in Eq. (5) for ${\varvec{G}}_1$ uses a polynomial approximation to degree 16, rather than C++’s built-in function, making use of the possible argument range with a guaranteed accuracy of 12 digits. Second, the code uses an optimised implementation of the complex matrix-matrix product for ${\varvec{W}}$ that runs above 60% of the processor’s peak arithmetic performance, using the best single-instruction$\,/\,$multiple-data (SIMD) capability of the processor. Note that a custom implementation, following matrix-matrix multiplication with intrinsics described in ref.^96^, Sec. 3.2, on a array-of-struct-of-array layout is used to ensure contiguous loads of real and imaginary parts. This approach avoids the data rearrangement costs necessary by BLAS-3 zgemm implementations^97^ for the given small column size and with $\varvec{W}$ exceeding the cache capacity. In addition, Table 2 shows a deviation of run time of Algorithm 1 for different values of *M* slightly larger than the statistical noise, even though it should not depend on *M*. This is because Algorithm 1 picks columns in $\widetilde{{\varvec{W}}}$ in slightly different orders, leading to a different run-time behaviour due to different cache access patterns despite the same amount of computations.

**Table 2:** Run time in $\textrm{ms}$ of Algorithm 3 on a single core of an AMD Ryzen 7 7840U CPU operated at 5.1 GHz and compiled with the gcc compiler version 13.2 with flags -O3 -march=znver4. The main steps of Algorithm 3 are indicated with lines of the algorithm statement, using the block-coordinate minimisation of Algorithm 1. All run times are the statistical mean of 50 runs rounded to two significant digits (statistical variations are around 2%).

size	${\varvec{G}}_1$, ${\varvec{p}}_0$	${\varvec{W}}$	Algorithm 1	finalise	total
$N\times M$	lines 4–6	line 7	l. 8–10	l. 11–14	time
	$i_\text {max}=N$ iterations				
256 × 14	0.07 ms	0.08 ms	0.24 ms	0.01 ms	0.40 ms
256 × 26	0.14 ms	0.15 ms	0.24 ms	0.01 ms	0.54 ms
256 × 38	0.26 ms	0.22 ms	0.24 ms	0.01 ms	0.73 ms
512 × 14	0.15 ms	0.31 ms	0.98 ms	0.01 ms	1.45 ms
512 × 26	0.30 ms	0.56 ms	0.98 ms	0.01 ms	1.85 ms
512 × 38	0.47 ms	0.78 ms	0.97 ms	0.01 ms	2.23 ms
1024 × 14	0.29 ms	1.16 ms	3.98 ms	0.03 ms	5.46 ms
1024 × 26	0.57 ms	2.06 ms	4.02 ms	0.03 ms	6.68 ms
1024 × 38	0.86 ms	3.05 ms	4.05 ms	0.03 ms	7.99 ms
	$i_\text {max}=2048$ iterations				
1024 × 14	0.29 ms	1.21 ms	7.04 ms	0.03 ms	8.57 ms
1024 × 14	0.57 ms	2.06 ms	7.05 ms	0.03 ms	9.71 ms
1024 × 38	0.86 ms	3.04 ms	7.07 ms	0.03 ms	11.00 ms

### Accuracy of the sound field recovery of component ${\textcircled {{2}}}$ ($\Delta \varvec{r} \rightarrow \varvec{\phi }^{*}$)

**Fig. 6:**
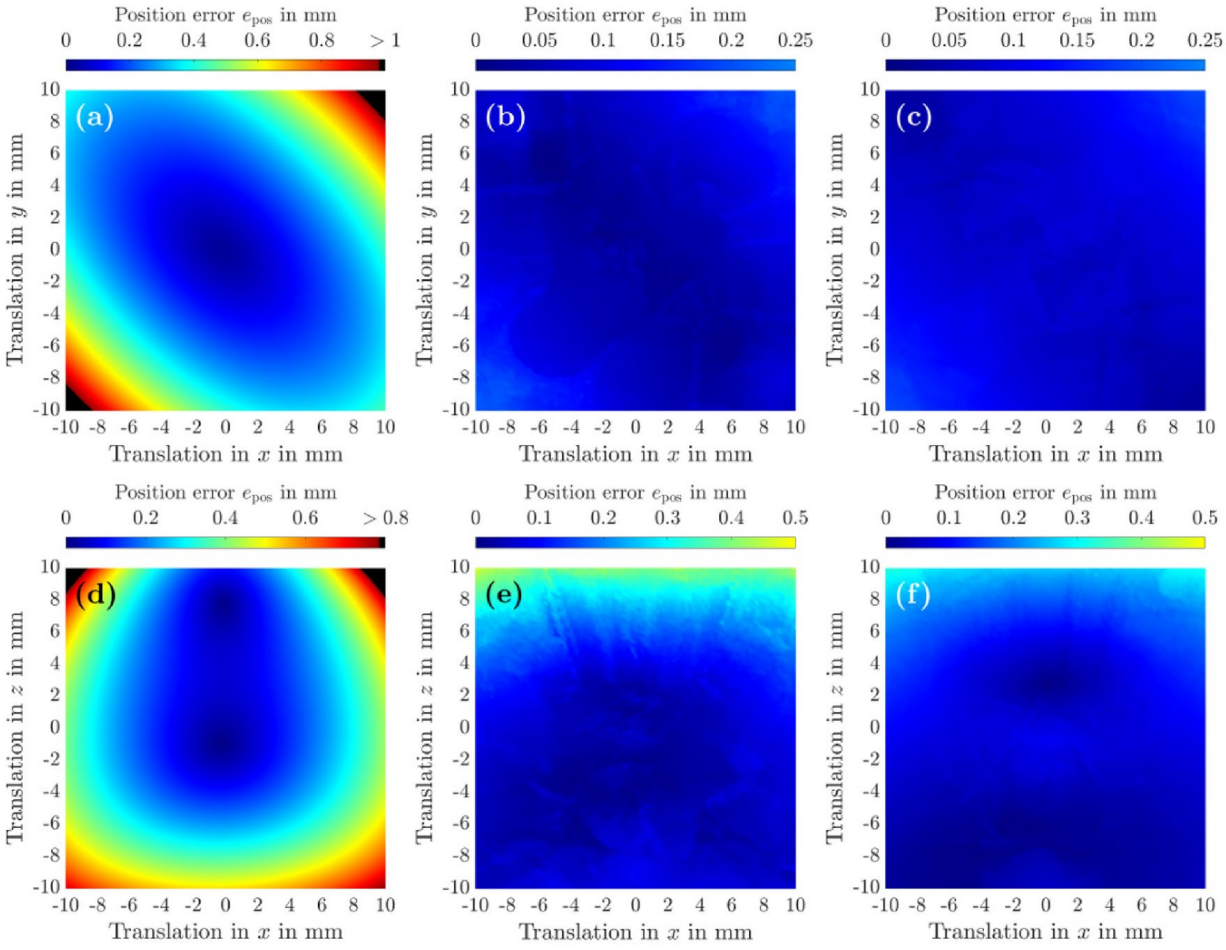
**Comparison of the sound field recovery accuracy of the holographic acoustic elements approach** [(**a**),(**d**)] **of Marzo et al.**^14^** described on p. 6. with Algorithm **3 **[(b),(c),(e),(f)].** In this simulative analysis, the settings from the experimental setup in Augsburg were used, starting with a *twin tunings forks trap*^16^ defined by $\varvec{\phi }_\textrm{opt}$ to levitate a EPS sphere of $d = 8.5\,\textrm{mm}$ in diameter and a mass of $m = 7.4\,\textrm{mg}$ at $\varvec{r}_\textrm{opt} = (0\,\varvec{e}_\textrm{x} + 0\,\varvec{e}_\textrm{y} + 50\,\varvec{e}_\textrm{z})\,\textrm{mm}$. From $\varvec{r}_\textrm{opt}$, the sound pressure field of the trap was translated by $\Delta x, \Delta y \in \left[ -10,10\right] \,\textrm{mm}$ in the *xy*-plane **[(a)**, **(b)**, **(c)]** as well as by $\Delta x, \Delta y \in \left[ -10,10\right] \,\textrm{mm}$ in the *xz*-plane **[(d)**, **(e)**, **(f)]**. To compare the accuracy of both algorithms, the position error $e_\textrm{pos} = \left\| \varvec{r}_\textrm{eq} - \varvec{r}_\textrm{trap} \right\| _2$ (see Eq. (24)) was used as a metric, denoting by $\varvec{r}_\textrm{trap}$ the new trap centre and by $\varvec{r}_\textrm{eq}$ the equilibrium position of the Mie sphere inside the shifted trap. To obtain $\varvec{r}_\textrm{eq}$ for each $\varvec{r}_\textrm{trap}$, we initialised sphere position at $\varvec{r}_\textrm{trap}$ and ran Algorithm 2 with $\varvec{r}_{\textrm{eq},0} = \varvec{r}_\textrm{trap}$, $\alpha = 20 \times 10^{-3}$, $F_\textrm{tol} = 1 \times 10^{-19}\,\mathrm {{N}}$, $r_\textrm{tol} = 5\,\textrm{mm},$and $i_\textrm{max} = 4000$ as initial values. In **(b)** and **(e)**, Algorithm 3 was executed with $\varvec{G} \in {\mathbb {R}}^{M \times N}$, whereas in **(c)** and **(f)**, Algorithm 3 employed $\widehat{\varvec{G}} \in {\mathbb {R}}^{4M \times N}$, $$\widehat{\varvec{G}} = \begin{pmatrix} \varvec{G}^\top\varvec{G}_\textrm{x}^\top\varvec{G}_\textrm{y}^\top\varvec{G}_\textrm{z}^\top \end{pmatrix}^\top$$ (see Eqs. (4)–(7)), also taking the spatial derivates of the pressure $\varvec{p} \in {\mathbb {C}}^{M \times 1}$ at the locations $\mathcal {M} = \left\{ \varvec{r}_1, \varvec{r}_2, \dots , \varvec{r}_M \right\}$ of the $M = 26$ selected nodes into account. In this case, we applied a balancing on $\varvec{W}$ by $\varvec{W} = \varvec{A}^{\textrm{H}} \varvec{\Lambda } \varvec{A}$, where $$\varvec{\Lambda } = \textrm{diag}\left( \begin{bmatrix} \varvec{1}^{1 \times M}&\left( 1\,/\,k\right) \cdot \varvec{1}^{1 \times 3M} \end{bmatrix}^\top \right) , \varvec{\Lambda } \in {\mathbb {R}}^{4M \times 4M}$$ and *k* denotes the wave number. Finally, in **[(b),(c),(e),(f)]**, Algorithm 3 used $L=1$ and $i_\textrm{max} = 1024$.

After evaluating the computational performance and real-time capability of Algorithm 3, we now investigate its accuracy with respect to the recovery of sound fields. Shifting a given trap by $\Delta \varvec{r}$ at high precision is a mandatory requirement for the inverse model in Fig. 2, since the assumption of a static radiation force distribution by the component ${\textcircled {{1}}}$ ($\varvec{F}^{*} \rightarrow \Delta \varvec{r})$ strongly relies on the accuracy of ${\textcircled {{2}}}$ ($\Delta \varvec{r} \rightarrow \varvec{\phi }^{*}$). The results of this simulative study are depicted in Fig. 6, where we compared Algorithm 3 with the popular *holographic acoustic elements* approach^14^ described on p. 6. As our focus lies on acoustic levitation and contactless manipulation, we evaluate the precision of both methods in shifting complex pressure fields with the metric $e_\textrm{pos} = \left\| \varvec{r}_\textrm{eq} - \varvec{r}_\textrm{trap} \right\| _2$, denoting by $\varvec{r}_\textrm{trap}$ the new trap centre and by $\varvec{r}_\textrm{eq}$ the equilibrium position of the Mie sphere inside the shifted trap. Thus, a value of $e_\textrm{pos} = 0$ means in this case that the acoustic trap can be perfectly shifted from its initial location $\varvec{r}_\textrm{opt}$ to the new location $\varvec{r}_\textrm{trap}$ by a total displacement of $\Delta \varvec{r} = \Delta r_\textrm{x}\varvec{e}_\textrm{x} + \Delta r_\textrm{y} \varvec{e}_\textrm{y} + \Delta r_\textrm{z} \varvec{e}_\textrm{z} = \varvec{r}_\textrm{trap} - \varvec{r}_\textrm{opt},$ whereas values of $e_\textrm{pos} \ge 0.5\,\textrm{mm}$ indicate a low recovery precision at $\varvec{r}_\textrm{trap}$, resulting in a high mismatch of $\varvec{r}_\textrm{trap}$ and $\varvec{r}_\textrm{eq}$. It should be noted that an acoustic trap usually cannot be shifted by $\Vert \Delta \varvec{r}\Vert _2 \ge 1.5\,\textrm{mm}$ in a single step in any control strategy, as high and immediate trap displacements are very likely to cause the levitated object to be ejected out of the trap. Therefore, the result depicted in Fig. 6 is not intended for an analysis of the dynamic behaviour of the object, but rather for a comparison of the feasible workspaces $\mathcal {W}$ that can be safely passed by the levitated object at moderate velocities, using either an open-loop control strategy like in refs.^48^^,^^68^ with $\Vert \Delta \varvec{r}\Vert _2 \le 0.2\,\textrm{mm}$ between two adjacent trap positions $\varvec{r}_{i}$ and $\varvec{r}_{j}$ or a closed-loop control strategy. As it can bee seen from Figs. 6(a,d), the *holographic acoustic elements* approach^14^ guarantees a recovery of the original trap at high precision ($e_\textrm{pos} \le 0.25\,\textrm{mm}$) only in a volume $\mathcal {V}_\mathrm {\left\{ (a),(d)\right\} }$ that is given by $\mathcal {V}_\mathrm {\left\{ (a),(d)\right\} } = \left\{ \Delta \varvec{r}\,\big\vert \, \Delta r_\textrm{x} \in \left[ -4,4\right] \textrm{mm}, \Delta r_\textrm{y} \in \left[ -4,4\right] \textrm{mm}, \Delta r_\textrm{z} \in \left[ -4,8\right] \textrm{mm} \right\}$. If the Mie sphere is translated from $\varvec{r}_\textrm{opt}$ to positions $\varvec{r}_i \notin \mathcal {V}_\mathrm {\left\{ (a),(d)\right\} }$, there are higher position errors which indicate a stronger mismatch of the radiation force distribution around the new trap centre, compared to the original distribution at positions $\varvec{r}_i$ adjacent to $\varvec{r}_\textrm{opt}$. Consequently, a dynamic manipulation of the Mie sphere in these regions along a discretised trajectory $\mathcal {T} = \left\{ \varvec{r}_0,\varvec{r}_1, \dots , \varvec{r}_n\right\}$ with an iterative refocusing strategy^14^ is strongly inhibited. Each trap shift from $\varvec{r}_i$ to a subsequent adjacent position $\varvec{r}_j$ by a feasible $\Delta \varvec{r}_{ij} = \varvec{r}_j - \varvec{r}_i$ will cause a strong mismatch between the predicted force $\widehat{\varvec{F}}_\textrm{res} = \varvec{P}(\Delta \varvec{r}_{ij},\varvec{A})$ (see ${\textcircled {{1}}}$ ($\varvec{F}^{*} \rightarrow \Delta \varvec{r})$ in Fig. 2) and the actual force $\varvec{F}_\textrm{res}$. This disturbing force $\varvec{F}_\textrm{err} = \varvec{F}_\textrm{res} - \widehat{\varvec{F}}_\textrm{res}$ exerted on the sphere is likely to cause the sphere to be ejected out of the trap on its course on $\mathcal {T}$.

In contrast, Figs. 6(b,e) shows that Algorithm 3 has a significantly higher precision in $$\mathcal {V}_\mathrm {\left\{ (a),(d)\right\} }$$ and enables a larger workspace, providing a volume $$\mathcal {V}_\mathrm {\left\{ (b),(e)\right\} } = \left\{ \Delta \varvec{r}\,\big \vert \, \Delta r_\textrm{x} \right.$$$$ \left.\in \left[ -10,10\right] \textrm{mm}, \Delta r_\textrm{y} \in \left[ -10,10\right] \textrm{mm}, \Delta r_\textrm{z} \in \left[ -10,5\right] \textrm{mm} \right\}$$ at the same accuracy of $$e_\textrm{pos} \le 0.25\,\textrm{mm}$$ as the approach presented by Marzo et al.^14^. It should be noted that $$e_\textrm{pos}$$ increases up to $$0.45\,\textrm{mm}$$ as the trap is shifted vertically by $$\Delta r_\textrm{z} \ge 8\,\textrm{mm}$$, indicating that the original acoustic trap around $$\varvec{r}_\textrm{opt}$$ cannot be reconstructed exactly for such large displacements, either in terms of the absolute pressure or its spatial distribution. This is probably caused by the decreasing contribution to the pressure field by each transducer as the distance to the object increases for all transducers of the PAT (compare Eq. (4)). This reduction usually cannot be fully compensated by an improved radiation characteristic, taken into account by the directivity $$D_\textrm{f}(\theta _j)$$ in the piston source model, see Eq. (5). To tackle this issue, Figs. 6(c,f) show the result of a modified version of Algorithm 3 where $$\widehat{\varvec{G}} = \begin{pmatrix} \varvec{G}^\top\varvec{G}_\textrm{x}^\top\varvec{G}_\textrm{y}^\top\varvec{G}_\textrm{z}^\top \end{pmatrix}^\top$$ with $$\widehat{\varvec{G}} \in {\mathbb {R}}^{4M \times N}$$, see Eqs. (4)-(7), instead of $$\varvec{G} \in {\mathbb {R}}^{M \times N}$$ was used. Here, the spatial derivatives $$\widetilde{\varvec{p}}_i \in {\mathbb {C}}^{P \times 1}, i \in \left\{ \textrm{x,y,z}\right\}$$ of the acoustic pressure $$\widetilde{\varvec{p}}$$ at the locations $$\mathcal {M} = \left\{ \varvec{r}_1, \varvec{r}_2, \dots , \varvec{r}_M \right\}$$ of the nodes were also taken into account. This modification apparently strongly facilitates the precise recovery of the complex pressure field of the trap. This idea is motivated by the fact that the acoustic radiation force $$\varvec{F}_\textrm{rad} \in {\mathbb {R}}^{3 \times 1}$$ exerted on a levitated object depends both on $$\widetilde{\varvec{p}}$$ and the spatial derivatives $$\widetilde{\varvec{p}}_i$$ on the object surface^15^. Using this approach, $$e_\textrm{pos}$$ still does not drop below $$0.10\,\textrm{mm}$$ for large parts of the considered areas, depending on the specific total trap displacement. We presume that this error is mainly caused by the low spatial resolution of the PAT^98^ and the present transducer directivity. Another possible reason could be that we do not consider the scattered sound field of the object to ensure the real-time capability of the approach. To reduce $$e_\textrm{pos}$$ even further, it might be necessary to directly map $$\widetilde{\varvec{p}}$$ and $$\widetilde{\varvec{p}}_i$$ according to the term given in Eq. (4) in ref.^15^ for the computation of $$\varvec{F}_\textrm{rad}$$ instead of considering only their linear contributions. However, this modification would lead to a non-linear problem of 4th order, which to the authors’ best knowledge cannot be solved using the proposed technique of semidefinite programming. Although there are clear indications that by simply reconstructing the complex-valued sound pressure field of the acoustic trap, its force distribution is also recovered to a large extent, there is no strong evidence for this. In consequence, no generalisable statement can be made as on how accurately an arbitrary configuration of an acoustic trap can be reconstructed at another location. Regarding this question, we thus have limited the scope of this work to the following simulative analysis.

### Accuracy of the linkage $\varvec{F}^{*} \leftrightarrow \Delta \varvec{r}$ of component ${\textcircled {{1}}}$

**Fig. 7:**
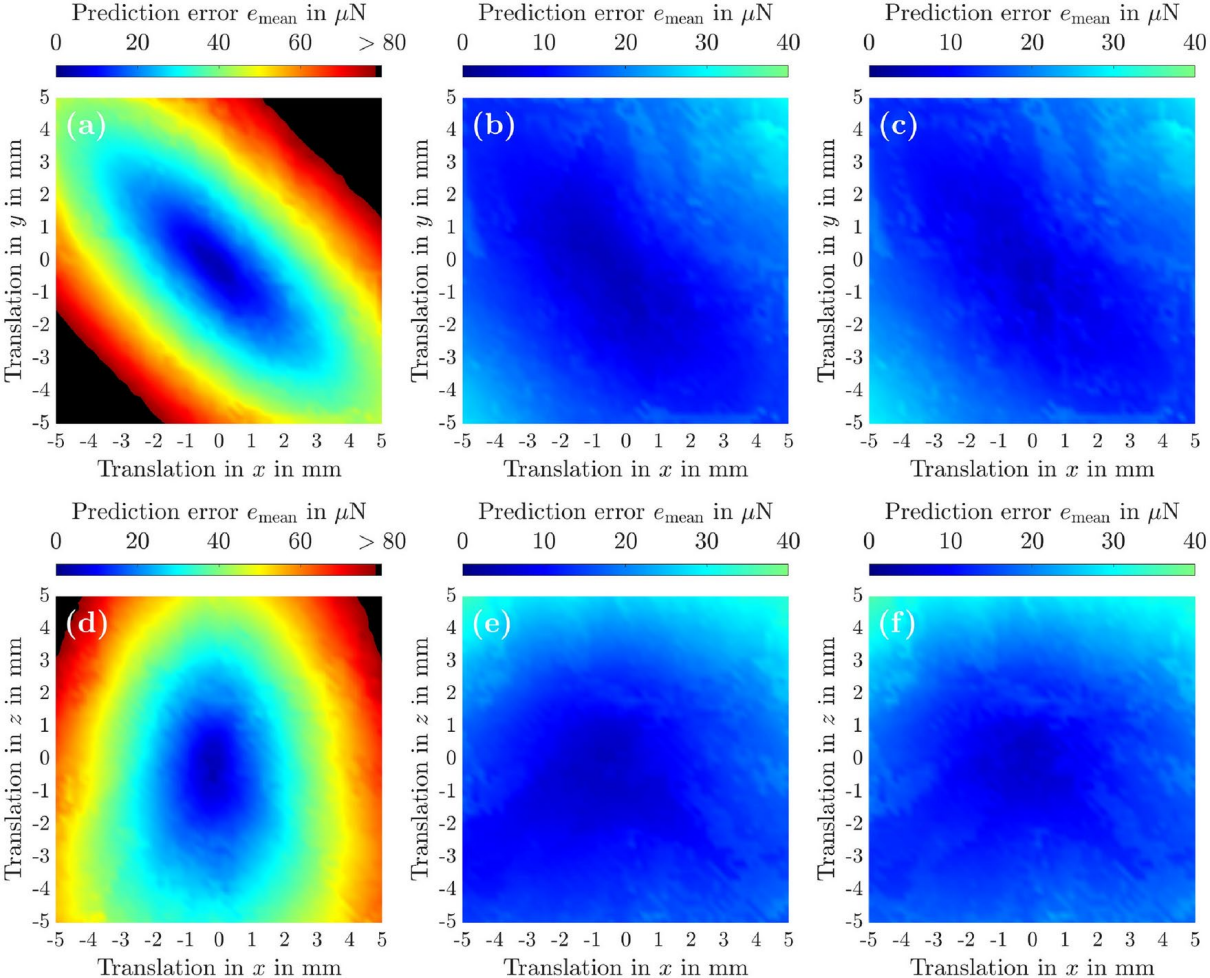
**Comparison of the force distribution recovery accuracy of the holographic acoustic elements approach** [(**a**),(**d**)]** of Marzo et al.**^14^ **described on p. 6. with Algorithm **3 **[(b),(c),(e),(f)].** In this simulative analysis, the same settings as in Fig. 6 were used. From $\varvec{r}_\textrm{opt} = (0\,\varvec{e}_\textrm{x} + 0\,\varvec{e}_\textrm{y} + 50\,\varvec{e}_\textrm{z})\,\textrm{mm}$, the sound pressure field of the optimised trap was translated by $\Delta x, \Delta y \in \left[ -5,5\right] \,\textrm{mm}$ in the *xy*-plane **[(a)**, **(b)**, **(c)]** as well as by $\Delta x, \Delta z \in \left[ -5,5\right] \,\textrm{mm}$ in the *xz*-plane **[(d)**, **(e)**, **(f)]**. To compare the accuracy of both algorithms, we employed Algorithm 4 and the prediction error $e_\textrm{mean}$ (see Eq. (32)) as a metric. For the training of $\widehat{\varvec{F}}_\textrm{res} = \varvec{P}(\Delta \varvec{r},\varvec{A})$, we selected $\delta = 3$, $\varvec{r}_\mathcal {W} = \varvec{r}_\textrm{opt}$, $R_\Omega = 6.25\,\textrm{mm}$ for $\mathcal {W} = \mathcal {V}(\varvec{r}_\mathcal {W},R_\Omega )$ and $R_\lambda = 1.25\,\textrm{mm}$ for $\mathcal {B} = \mathcal {V}(\varvec{r}_\textrm{opt},R_\lambda ) \subset \mathcal {W}$, see Eq. (1). Sampling $\mathcal {B}$ and $\mathcal {V}(\varvec{0},R_\lambda )$ in cubic grids with spacings of $0.125\,\textrm{mm}$ and $0.25\,\textrm{mm}$ respectively resulted in $I = 9261$ data tuples and $J = 1331$ relative displacements $\Delta \varvec{r}_j \in \mathcal {V}(\varvec{0},R_\lambda )$, $j = \left\{ 1,2,\dots ,J\right\}$, where the local force distribution was evaluated. To calculate $\varvec{F}_\textrm{rad}$, we used the approach in^17^. To evaluate $\widehat{\varvec{F}}_\textrm{res} = \varvec{P}(\Delta \varvec{r},\varvec{A})$, we sampled $\mathcal {V}(\varvec{r}_\mathcal {W},R_\Omega - R_\lambda ) \subset \mathcal {W}$ with a cubic grid with $0.2\,\textrm{mm}$ spacing, resulting in $L = 2601$ test positions. In **(b)** and **(e)**, Algorithm 3 was executed with $\varvec{G} \in {\mathbb {R}}^{M \times N}$, whereas in **(c)** and **(f)**, Algorithm 3 employed $\widehat{\varvec{G}} \in {\mathbb {R}}^{4M \times N}$, $$\widehat{\varvec{G}} = \begin{pmatrix} \varvec{G}^\top\!&\!\varvec{G}_\textrm{x}^\top\!&\!\varvec{G}_\textrm{y}^\top\!&\!\varvec{G}_\textrm{z}^\top \end{pmatrix}^\top$$ (see Eqs. (4)-(7)). Finally, in **[(b),(c),(e),(f)]**, Algorithm 3 was executed with $L=1$ and $i_\textrm{max} = 1024$.

In this analysis, we compare the force distribution recovery accuracy of the *holographic acoustic elements* approach^14^ with component ${\textcircled {{1}}}$ that establishes the linkage $\varvec{F}^{*} \leftrightarrow \Delta \varvec{r}$. To ensure a fair comparison, we restricted the feasible workspace $\mathcal {W}$ in this example to $\mathcal {W} = \mathcal {V}(\varvec{r}_\textrm{opt},6.25\,\textrm{mm})$, since both algorithms yielded similar position errors $e_\textrm{pos}$ in this volume, see Fig. 6 for further details. Figs. 7(a,d) shows the method of *holographic acoustic elements* which presents high recovery accuracies $(e_\textrm{mean} \le 30\,\mathrm {\mu N})$ of the resulting force distribution of the translated acoustic trap only in a small volume $\mathcal {V}_\mathrm {\left\{ (a),(d)\right\} } = \left\{ \Delta \varvec{r}\,\big\vert \, \Delta r_i \in \left[ -2,2\right] \textrm{mm}, i = \left\{ \textrm{x},\textrm{y},\textrm{z}\right\} \right\}.$ In contrast, the identified model $\widehat{\varvec{F}}_\textrm{res} = \varvec{P}(\Delta \varvec{r},\varvec{A})$ in Figs. 7(b,e) provides the same prediction accuracy in a much bigger volume, namely $\mathcal {V}_\mathrm {\left\{ (b),(e)\right\} } = \left\{ \Delta \varvec{r}\,\big\vert \, \Delta r_i \in \left[ -4,4\right] \textrm{mm}, i = \left\{ \textrm{x},\textrm{y},\textrm{z}\right\} \right\}$. As it can be seen by supplementary Fig. S1, the advantage of component ${\textcircled {{1}}}$ over the *holographic acoustic elements* approach becomes even clearer if instead of $16\,\textrm{Vpp}$ only $10\,\textrm{Vpp}$ are used to excite the transducers of the PAT. Furthermore, it can be inferred from Figs. 7(c,f) and Figs. S1(c,f) that including the spatial derivatives $\widetilde{\varvec{p}}_i$ in Algorithm 3 apparently does not improve the recovery accuracy of the force distribution significantly. This is in contrast to the improvement of the position errors $e_\textrm{pos} = \left\| \varvec{r}_\textrm{eq} - \varvec{r}_\textrm{trap} \right\| _2$ in Fig. 6. Finally, it is noteworthy that a bigger workspace $\mathcal {W}$ for component ${\textcircled {{1}}}$ can be generated by simply concatenating several volumes $\mathcal {V}_i$, in which corresponding polynomial models have been identified. In this case, an additional criteria in the dynamic model based on the current position $\varvec{r}$ of the levitated object has to be defined to properly switch between these polynomial models.

### Manipulation capabilities of the algorithm

**Fig. 8:**
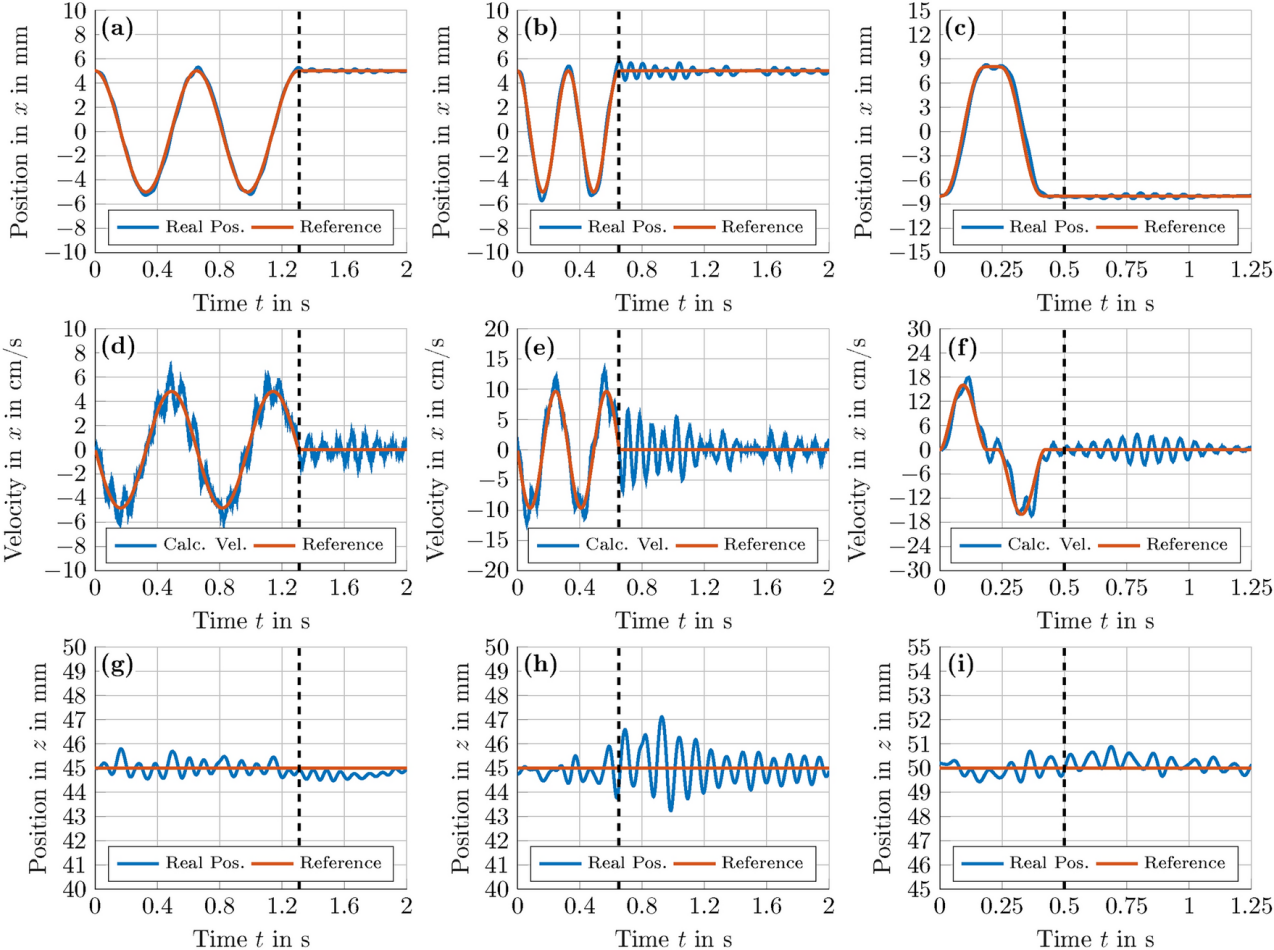
**Comparison of a kinematic and an optimal feed-forward control for a horizontal translation of a Mie sphere at various speeds**. In the experiments conducted in São Paulo [**(a),(b),(d),(e),(g),(h)**], an expanded polystyrene (EPS) sphere of $d = 8.1\,\textrm{mm}$ in diameter $(d\,/\lambda \approx 0.93)$ was first suspended at $\varvec{r} = \left( 5\,\varvec{e}_\textrm{x}+0\, \varvec{e}_\textrm{y}+45\,\varvec{e}_\textrm{z}\right) \,\textrm{mm}$ in a *twin tuning forks trap*^16^ and then manipulated with Algorithm 3 by altering the trapping position in $r_\textrm{x}$ according to $r_\textrm{x}(t)=A \cos (\omega t)$ using a kinematic open loop control, where $A = 5\,\textrm{mm}$ is the translation amplitude and the angular frequency $\omega$ was set to $\omega = 9.64\, \textrm{rad}\,\textrm{s}^{-1}$ **[(a),(d),(g)]** for a slow and to $\omega = 19.28\, \textrm{rad}\,\textrm{s}^{-1}$ [**(b),(e),(h)**] for a fast motion. In addition, a similar experiment was conducted in Augsburg [**(c),(f),(i)**]. Here, an EPS sphere of $8.5\,\textrm{mm}$ in diameter $(d\,/\lambda \approx 0.97)$ was first suspended at $\varvec{r} = \left( -8\,\varvec{e}_\textrm{x}+0\,\varvec{e}_\textrm{y}+50\,\varvec{e}_\textrm{z}\right) \,\textrm{mm}$ in a *twin tuning forks trap*^16^ and then manipulated in *x*-direction along the given reference trajectory with a model-based optimal feed-forward control. Furthermore, the black dashed lines in [**(a)-(i)**] indicate the last change of the activation $\varvec{\phi }$ of the PAT. Supplementary videos 2 and 3 show the manipulation of the Mie sphere using a kinematic ([**(a),(d),(g)**] & [**(b),(e),(h)**]) and a model-based optimal feed-forward control [**(c),(f),(i)**], respectively.

The real-time capability of the presented method was first investigated in an experiment conducted in the laboratory in São Paulo, see section Experimental setup for details. Here, an EPS particle of $d = 8.1\,\textrm{mm}$ in diameter $(d/\lambda \approx 0.93)$ was first suspended at $\varvec{r} = (5\,\varvec{e}_\textrm{x}+0\,\varvec{e}_\textrm{y}+45\, \varvec{e}_\textrm{z})\,\textrm{mm}$ and then manipulated by altering the trapping position in $r_\textrm{x}$ at various speeds using a kinematic open-loop control. This terminology means that only the component ${\textcircled {{2}}}$ ($\Delta \varvec{r} \rightarrow \varvec{\phi }^{*}$) with Algorithm 3 was used to directly shift the given acoustic trap along the reference trajectory, not taking the resulting forces or the dynamic behaviour of the levitated Mie sphere inside the acoustic trap into account. The experimental results for a slow and a fast horizontal motion are presented in Figs. 8(a,d,g) and Figs. 8(b,e,h), respectively. The motion of the sphere can also be seen in the supplementary movie 2. Figs. 8(d,e) show a top speed of the sphere of $v_\textrm{max}\approx 50\,\textrm{mm}\,\textrm{s}^{-1}$ for the slow motion and $v_\textrm{max}\approx 100\,\textrm{mm}\,\textrm{s}^{-1}$ for the fast motion. In Fig. 8(a), the sphere follows the reference trajectory at slow motion and presents almost no oscillation when it reaches $r_x = 5\,\textrm{mm}$ at $t\approx 1.3\,\textrm{s}$. However, for the fast motion depicted in Fig. 8, the sphere displays a small horizontal oscillation after two manipulation cycles. Despite this small oscillation, this experiment clearly indicates that Algorithm 3 is able to manipulate a Mie sphere with sufficient precision in real time.

To improve these results, similar experiments were conducted in Augsburg. Instead of a kinematic open-loop control, an EPS sphere of $d = 8.5\,\textrm{mm}$ $(d/\lambda \approx 0.97)$ was manipulated by means of an optimal feed-forward control to follow given paths, taking the dynamic behaviour of the Mie sphere into account. To obtain such a control, we first determined a feasible $\varvec{\phi }_\textrm{opt}$ for the PAT using a non-linear optimisation^16,17^, creating a *twin tuning forks trap*^16^ which stably suspends the sphere at $\varvec{r}_\textrm{opt} = \left( 0\,\varvec{e}_\textrm{x}+0\,\varvec{e}_\textrm{y}+50\,\varvec{e}_\textrm{z}\right) \,\textrm{mm}$. Subsequently, by applying a polynomial regression, we approximated the force distribution of the trap in a cubic volume

1 $$\begin{aligned} \mathcal {V}(\varvec{r}_\textrm{opt},R_\lambda ) = \left\{ \varvec{r}_i \in {\mathbb {R}}^3 \,\big \vert \,\vert {r}_{j,i} - {r}_{j,\textrm{opt}} \vert \le R_\lambda , R_\lambda \in {\mathbb {R}}^{+}, j \in \left\{ \textrm{x,y,z}\right\} \right\} \end{aligned}$$

centred around $\varvec{r}_\textrm{opt}$ with an edge length $2R_\lambda = 2.50\,\textrm{mm}$. Next, the resulting multivariate polynomial models $\varvec{F}_\textrm{res} = \varvec{P}\left( \Delta \varvec{r}, \varvec{A} \right)$ and $\Delta \varvec{r} = \varvec{P}\left( \varvec{F}_\textrm{res},\varvec{B}\right)$ with total degree $\delta = 3$ were validated in $\mathcal {V}(\varvec{r}_\mathcal {W},R_\Omega - R_\lambda ) \subset \mathcal {W},$ where the workspace $\mathcal {W} = \mathcal {V}\left( \varvec{r}_\mathcal {W},R_\Omega \right)$ centred at $\varvec{r}_\mathcal {W} = \left( 0\,\varvec{e}_\textrm{x}+0\,\varvec{e}_\textrm{y}+50\,\varvec{e}_\textrm{z}\right) \,\textrm{mm}$ had an edge length of $2R_\Omega = 25\,\textrm{mm}$ (see section Training and validation of the polynomial models for details).

To obtain an optimal feed-forward control for the given reference trajectories, we applied a model prective control (MPC)^99^ algorithm on the corresponding path-following problems. Finally, an obtained sequence of trap shifts by $\Delta \varvec{r}_{i}$ is fed to component ${\textcircled {{2}}}$ ($\Delta \varvec{r} \rightarrow \varvec{\phi }^{*}$) of the inverse model in Fig. 2 to calculate a feasible control sequence of activations $\varvec{\phi }_i$ with Algorithm 3 for the given trap at $\varvec{r}_\textrm{opt}$ which is defined by $\varvec{\phi }_\textrm{opt}$.

**Fig. 9:**
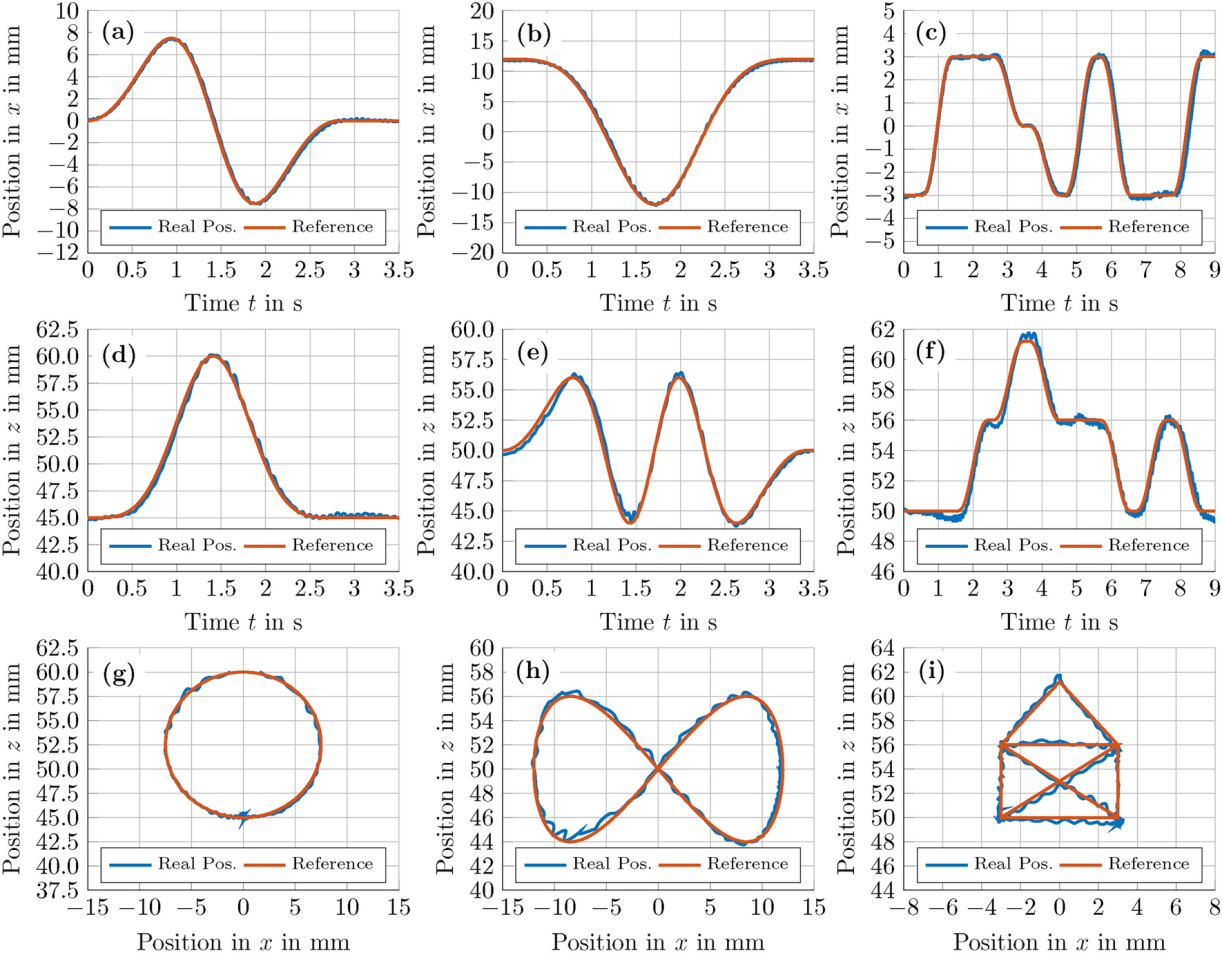
**Manipulation of an EPS sphere of** $\mathbf {8.5\,mm}$ **in diameter along various trajectories using an optimal feed-forward control**. Similar to the experiment presented in Fig. 8(c,f,i), a MPC algorithm^99^ was employed to solve the corresponding path-following problems. Supplementary movies 4-6 show the manipulation of the Mie sphere using the experimental setup in Augsburg along the circle **[(a),(d),(g)]**, the infinite symbol **[(b),(e),(h)]**, and the cross house trajectory **[(c),(f),(i)]**, respectively.

After stating the necessary steps of implementing an optimal feed-forward control, we now take a look at the results obtained in Augsburg. Comparing Figs. 8(c,f) with Figs. 8(b,e), the model-based optimal feed-forward control enables a faster horizontal manipulation of the Mie sphere, which reaches a top speed of $v_\textrm{max}\approx 150\,\textrm{mms}^{-1}$ in this experiment, see also Fig. 8(f). Despite the high velocities, the Mie sphere displays minimal oscillations when reaching its final position $\varvec{r} = (-8\,\varvec{e}_\textrm{x}+0\,\varvec{e}_\textrm{y}+50\,\varvec{e}_\textrm{z})\,\textrm{mm}$ at $t \approx 0.4\,\textrm{s}$. The horizontal motion of the sphere can also be seen in the supplementary movie 3. In further experiments, we translated the levitated Mie sphere along more complex trajectories, namely a circular (Figs. 9(a,d,g)), an infinite symbol (Figs. 9(b,e,h)) and a cross house (Figs. 9(c,f,i)) trajectory. The path of the sphere for the experiments depicted in Fig. 9 can also be seen in the corresponding supplementary videos 4-6. Starting from $\varvec{r}_0 = \left( 0\,\varvec{e}_\textrm{x}+0\,\varvec{e}_\textrm{y}+45\,\varvec{e}_\textrm{z}\right) \,\textrm{mm}$, the reference circular trajectory depicted in Figs. 9(a,d,g) is given by

2 $$\begin{aligned} \begin{aligned} r_\textrm{x}(t)&= A \sin \left( \pi \left( 1 - \cos \left( \omega t\right) \right) \right) \quad \text {and} \\ r_\textrm{z}(t)&= r_{\textrm{z},0} + A - A \cos \left( \pi \left( 1 - \cos \left( \omega t\right) \right) \right) , \end{aligned} \end{aligned}$$

where $t \in \left[ 0,3\right] \,\textrm{s}$, $A = 7.5\,\textrm{mm}$, and $\omega = \pi /3$. For the infinite symbol trajectory starting at $\varvec{r}_0 = \left( 12\,\varvec{e}_\textrm{x}+0\,\varvec{e}_\textrm{y}+50\,\varvec{e}_\textrm{z}\right) \,\textrm{mm}$, $r_\textrm{x}(t)$ and $r_\textrm{z}(t)$ are in turn defined by the following two equations:

3 $$\begin{aligned} \begin{aligned} r_\textrm{x}(t)&= A \cos \left( \pi \left( 1 - \cos \left( \omega t\right) \right) \right) \\ r_\textrm{z}(t)&= r_{\textrm{z},0} + 0.5 A \sin \left( 2 \pi \left( 1 - \cos \left( \omega t\right) \right) \right) . \end{aligned} \end{aligned}$$

In Eq. (3), the parameters *A*, $\omega$, and *t* are given by $A = 12\,\textrm{mm}$, $\omega = \pi /3.5$ and $t\,\in \left[ 0,3.5\right]$. Finally, for the cross house trajectory, the reference trajectory begins at $\varvec{r}_0 = \left( -3\,\varvec{e}_\textrm{x}+0\,\varvec{e}_\textrm{y}+50\,\varvec{e}_\textrm{z}\right) \,\textrm{mm}$ at the lower left corner and the Mie sphere is iteratively commanded to each corner of the house (see Fig. 9(i)) along the path depicted in Figs. 9(c,f), reaching the final position $\varvec{r}_\textrm{T} = \left( 3\,\varvec{e}_\textrm{x}+0\,\varvec{e}_\textrm{y}+50\,\varvec{e}_\textrm{z}\right) \,\textrm{mm}$ at $t \approx 8.75\,\textrm{s}$. To obtain the reference trajectory, we simply concatenated consecutive corner positions by polynomials with minimal curvature.

Taking a look at the obtained experimental results, it can be seen from Fig. 9(a,d,g) that the Mie sphere moves smoothly along the circular path, reaching top speeds of $v_\textrm{max}\approx 30\,\textrm{mm}\,\textrm{s}^{-1}$ and presenting maximum position errors of $e_\textrm{max} \approx 1\,\textrm{mm}$ during its motion. Considering the long manipulation horizon and the numerous unknown disturbances caused by typical acoustic phenomena like acoustic streaming^53,54^, acoustic viscous torques^55^, and harmonic generation^56^, which are all not considered in our dynamic model, these are very good results. It is noteworthy that the results of our model-based optimal open-loop control are achieved without any position feedback by a camera system. Furthermore, these initial impressions are confirmed by the experiment presented in Figs. 9(b,e,h), where the Mie sphere moves along an infinite symbol trajectory defined by Eq. (3), reaching a maximum velocity of $v_\textrm{max}\approx 50\,\textrm{mm}\,\textrm{s}^{-1}$. Here, the sphere moves smoothly along the given reference trajectory with minimal oscillations, resulting in a maximum position error of $e_\textrm{pos} \approx 0.8\,\textrm{mm}.$ The main oscillatory behaviour of the sphere occurs around the position $\varvec{r} = \left( -8\,\varvec{e}_\textrm{x}+0\,\varvec{e}_\textrm{y}+44\,\varvec{e}_\textrm{z}\right) \,\textrm{mm}$ at $t \approx 1.4\,\textrm{s}$ during a sharp change in acceleration, which apparently cannot be fully reflected by our chosen dynamic model. If such changes in acceleration are smaller, for example when the sphere is passing the point $\varvec{r} = \left( 8\,\varvec{e}_\textrm{x}+0\,\varvec{e}_\textrm{y}+44\,\varvec{e}_\textrm{z}\right) \,\textrm{mm}$ at $t \approx 2.6\,\textrm{s}$, the sphere presents significantly smaller oscillations, indicating a good agreement between the chosen dynamic model and the experimental results. Finally, after two smooth trajectories, the sphere is moved along a path with sharp bends, namely the cross house trajectory depicted in Figs. 9(c,f,i). In this experiment, the Mie sphere reaches a top speed of $v_\textrm{max}\approx 25\,\textrm{mm}\,\textrm{s}^{-1}$ and maximum position error of $e_\textrm{pos} \approx 0.6\,\textrm{mm}$. Similar to the infinite symbol trajectory the sharp changes in acceleration pose minor problems for the optimal model-based feed-forward control, resulting in a slight jitter during the movement of the sphere. However, these small oscillations are common for acoustic levitation systems that are driven by a (model-based) open-loop control. In general we show that the proposed algorithm is capable of moving a Mie sphere along the chosen trajectories inside the working space with minimal position error. The optimal model-based feed-forward control is able to improve these results even further by taking the dynamic behaviour of the Mie sphere into account.

## Conclusion

This work presents a real-time capable inverse model that allows the unambiguous determination of a feasible activation $\varvec{\phi }^{*}$ for a PAT from a demanded control $\varvec{F}^{*}$ (see Fig. 1), paving the road for model-based closed-loop control to overcome current limitations of acoustic levitation systems such as low speed, undesired oscillations of the levitated object or reduced precision in positioning. To establish the relation $\varvec{F}^{*} \rightarrow \varvec{\phi }^{*}$, the inverse model in Fig. 2 is comprised of the components ${\textcircled {{1}}}$ ($\varvec{F}^{*} \rightarrow \Delta \varvec{r})$ and ${\textcircled {{2}}}$ ($\Delta \varvec{r} \rightarrow \varvec{\phi }^{*}$). To obtain such an inverse model, the following steps must be carried out. At first, a feasible activation $\varvec{\phi }_\textrm{opt}$ for an initial trap at $\varvec{r}_\textrm{opt}$ is obtained in the *offline phase* using a non-linear optimisation approach^15–17^. Subsequently, the radiation force distribution of the trap is approximated by a polynomial regression, identifying the parameter matrices $\varvec{A}$ and $\varvec{B}$ of the the two real-time capable models $\varvec{F}_\textrm{res} = \varvec{P}\left( \Delta \varvec{r}, \varvec{A}\right)$ and $\Delta \varvec{r} = \varvec{P}\left( \varvec{F}^{*}, \varvec{B}\right)$. The models in turn are used in the *online phase* to trace back the desired force $\varvec{F}^{*}$ on the levitated object to a displacement $\Delta \varvec{r}$ of the trap centre from the current position $\varvec{r}$ of the object and vice versa. Since $\varvec{F}_\textrm{rad}$ is related to the complex pressure exerted on the object surface^14,15^, a trap is exactly shifted to a new location if its original pressure distribution can be recreated there. Besides the prediction of $\varvec{F}_\textrm{res}$ and $\Delta \varvec{r}$, this insight reduces the remaining tasks in the crucial *online phase* (see Fig. 2) to one. This task is obtaining a feasible activation $\varvec{\phi }^{*}$ for the PAT in real time in order to shift the trap from its last position by $\Delta \varvec{r}$. For this purpose, we employ the results of Waldspurger et al.^57^ and relax the phase recovery problem to an SDP called *PhaseCut*, which is very similar to the *MaxCut* problem. This enables the adaption of recent approaches for *MaxCut* for *PhaseCut*, leading to the development of the real-time capable Algorithm 3 that is based on a block-coordinate minimisation (BCM) algorithm and the Burer-Monteiro method. We identify that BCM with a *greedy* coordinate selection strategy is an excellent choice for the associated linear SDP, as it comes with a very favourable complexity and run-time behaviour. Furthermore, our proposed method comes with exceptionally low computational costs (see Fig. 5), since it only requires a single matrix-vector product at startup, followed by updates of a single column, which results in a favourable complexity of $\mathcal O(N)$ operations per iteration when using a single-vector strategy ($L = 1$) in Algorithm 1. Even for big PATs with $N = 1024$ transducers, the phase retrieval problem can be solved in less than approximately $11\,\textrm{ms}$ on a *single core* of a modern CPU in our examples in Table 2, strongly facilitating its usage in future real-time applications. Furthermore, even larger setups are conceivable when using parallel implementations of the algorithm and more powerful hardware such as GPUs.

For applications such as model-based open or closed-loop control, our proposed inverse model provides a sound basis, whether directly being employed in the loop or as knowledge basis for the design of an optimal feed-forward control. The latter was demonstrated by our experimental investigations, in which we were able to precisely manipulate a Mie sphere along different complex paths such as a circle, an infinite symbol, and cross house trajectory (see Fig. 9) at high velocities using an optimal feed-forward control. Finally, due to the clear inference from $\varvec{F}^{*}$ to $\varvec{\phi }^{*}$ in real time (see Fig. 1) provided by our proposed inverse model, the present work makes a noteworthy contribution to the design of closed-loop circuits for future acoustic levitation systems, not only allowing the precise contactless handling of Rayleigh objects, but also the dynamic manipulation of objects in the Mie regime.

## Methods

### Acoustic pressure field

To calculate the acoustic pressure $p_j(\varvec{r})$ at $\varvec{r} = r_\textrm{x} \varvec{e}_\textrm{x} + r_\textrm{y} \varvec{e}_\textrm{y} + r_\textrm{z} \varvec{e}_\textrm{z}$ that is created by a single acoustic transducer at $\varvec{r}_{\textrm{t},j} = r_{\textrm{t},\textrm{x},j} \varvec{e}_\textrm{x} + r_{\textrm{t},\textrm{y},j} \varvec{e}_\textrm{y} + r_{\textrm{t},\textrm{z},j} \varvec{e}_\textrm{z}$ emitting waves with a constant frequency $f_0$, we employ the piston source model in the far-field^100^ that is given as

4 $$\begin{aligned} p_j(\varvec{r}) = \frac{P_j A_j e^{\textrm{i}\phi _j}}{\Vert \varvec{r} - \varvec{r}_{\textrm{t},j} \Vert _2} D_\textrm{f} (\theta _j) e^{\textrm{i} k \Vert \varvec{r} - \varvec{r}_{\textrm{t},j} \Vert _2 }, \end{aligned}$$

where $\Vert \cdot \Vert _2$ denotes the Euclidean distance, i$\mathrm{i}$ is the imaginary unit, $P_j$ denotes the sensitivity of the transducer, $A_j$ and $\phi _j$ are the transducer’s amplitude and phase angle, and $k = \frac{2 \pi f_0}{c_0}$ is the wave number where $c_0$ is the speed of sound. The directivity function is taken as

5 $$\begin{aligned} D_\textrm{f}(\theta _j) = \frac{2J_1(k r_\textrm{p} \sin (\theta _j))}{k r_\textrm{p} \sin (\theta _j)}, \end{aligned}$$

where $J_1$ is the first-order Bessel function of the first kind, $r_\textrm{p}$ is the radius of the piston, and $\theta _j$ denotes the angle between the transducer normal $\varvec{n}_j = n_{\textrm{x},j} \varvec{e}_\textrm{x} + n_{\textrm{y},j} \varvec{e}_\textrm{y} + n_{\textrm{z},j} \varvec{e}_\textrm{z}$ and the vector $\varvec{r} - \varvec{r}_{\textrm{t},j}$. Owing to linearity, the total acoustic pressure $\widetilde{p}(\varvec{r})$ that is created by a transducer array of *N* sources can be calculated by superimposing the contributions of all emitted waves: $\widetilde{p}(\varvec{r}) = \sum _{j=1}^{N} p_j(\varvec{r}).$ This relation is also valid for the spatial derivatives of $\widetilde{p}(\varvec{r})$ that are given by $\widetilde{p}_h(\varvec{r}) = \sum _{j=1}^{N} p_{h,j}(\varvec{r}), h \in \left\{ \textrm{x},\textrm{y},\textrm{z} \right\}$^14^. The pressure field $\varvec{p} \in {\mathbb {C}}^{M \times 1}$ that is represented by a set of points $\mathcal {M} = \left\{ \varvec{r}_1, \varvec{r}_2, \dots , \varvec{r}_M \right\}$ is thus given by $\varvec{p} = \varvec{G} \varvec{q}$, where the activation of the transducers with respect to their phase angles is denoted by $$\varvec{q} \in \begin{pmatrix} e^{\textrm{i}\phi _1}&e^{\textrm{i}\phi _2}&\dots&e^{\textrm{i}\phi _N} \end{pmatrix}^\top \in {\mathbb {C}}^{N \times 1}$$. The propagator matrix $\varvec{G} \in {\mathbb {C}}^{M \times N}$ has the elements

6 $$\begin{aligned} G_{i,j} = G(\varvec{r}_i,\varvec{r}_{\textrm{t},j}) = \frac{P_j A_j D_\textrm{f} (\theta _{i,j})}{\Vert \varvec{r}_i - \varvec{r}_{\textrm{t},j} \Vert _2} e^{\textrm{i} k \Vert \varvec{r}_i - \varvec{r}_{\textrm{t},j} \Vert _2}, \end{aligned}$$

which describe the wave propagation from each transducer to each point of the sound pressure field. Consequently, the spatial derivatives $\widetilde{\varvec{p}}_h$ can be written as $\widetilde{\varvec{p}}_h = \varvec{G}_h \varvec{q}$, where the elements $G_{h,i,j}$ of $\varvec{G}_h \in {\mathbb {C}}^{M \times N}$ are given by

7 $$\begin{aligned} G_{h,i,j} = \frac{\partial G_{ij}}{\partial h}, \ h \in \left\{ \textrm{x},\textrm{y},\textrm{z}\right\} . \end{aligned}$$

Note that in Eqs. (6,7) we assume that all transducer amplitudes remain constant and the phase angles are solely used to create different pressure fields. This limitation is motivated by the fact that our hardware^98^ only supports phase modulation, which is common for several transducer arrays that were presented in the last years, see also Table 1 in^101^.

### Semidefinite programming

To create a desired pressure field $\varvec{p} \in {\mathbb {C}}^{M \times 1}$, a feasible $\varvec{q}^{*}\in {\mathbb {C}}^{N \times 1}$ fulfilling $\varvec{p} = \varvec{G} \varvec{q}^{*}$ has to be found. To determine $\varvec{q}^{*}$, a complex least squares problem with quadratic constraints can be formulated:

8 $$\begin{aligned} \begin{aligned} \min \limits _{\varvec{q}} \ &\Vert \varvec{G}\varvec{q} - \varvec{p} \Vert _{2} \\ \quad \text {s.t.} \ &|q_i |= 1 \ \qquad \forall i \in \left\{ 1,2,\dots ,N \right\} . \end{aligned} \end{aligned}$$

This problem can be effectively solved by semidefinite programming^58^. For this purpose, we add a variable $s \in {\mathbb {C}}$ that is subject to the same constraint as the elements ${q_i}$:

9 $$\begin{aligned} \begin{aligned} \min \limits _{\varvec{q},s} \ &\Vert \varvec{G}\varvec{q} - s \varvec{p} \Vert _{2} \\ \quad \text {s.t.} \ &|q_i |= 1 \ \qquad \forall i \in \left\{ 1,2,\dots ,N \right\} , \\&\ |s |= 1. \end{aligned} \end{aligned}$$

This does not alter the character of $\varvec{q}^{*}$, since, if $\varvec{q}^{*}$ and *s* are both feasible, *s* is only a constant added to each phase angle. Thus, a solution for Eq. (8) can easily be derived from a solution $\varvec{q}^{*}$ for Eq. (9). By introducing $$\varvec{z} = \begin{pmatrix} \varvec{q}^\top s \end{pmatrix}^\top \in {\mathbb {C}}^{(N+1) \times 1}$$ and $$\varvec{A} = \begin{pmatrix} \varvec{G}-\varvec{p} \end{pmatrix} \in {\mathbb {C}}^{M \times (N+1)}$$, the optimisation problem in Eq. (9) can be reformulated as follows:

10 $$\begin{aligned} \begin{aligned} \min \limits _{\varvec{z}} \ &\Vert \varvec{A}\varvec{z} \Vert _{2} \\ \quad \text {s.t.} \ &|z_i |= 1 \ \qquad \forall i \in \left\{ 1,2,\dots ,N+1 \right\} . \end{aligned} \end{aligned}$$

If $\varvec{z}^{*}$ is a feasible solution to Eq. (10), $\varvec{q}^{*}$ for Eq. (8) is determined by selecting the first *N* components of $\varvec{z}^{*},\varvec{z}^{*}_{(1:N)}$, and dividing each element by $z^{*}_{(N+1)}$:

11 $$\begin{aligned} \varvec{q}^{*} = \left( \frac{\varvec{z}^{*}_{(1:N)}}{z^{*}_{(N+1)}}\right) . \end{aligned}$$

The problems in Eqs. (8, 10) are non-convex with quadratically constrained variables and therefore difficult to solve. To simplify them, we use the relation $\Vert \varvec{A}\varvec{z} \Vert _{2}^{2} = \textrm{tr}(\varvec{A}^{\textrm{H}} \varvec{A} \varvec{z}\varvec{z}^{\textrm{H}}) = \textrm{tr}(\varvec{W} \varvec{Z})$, where $\varvec{W} = \varvec{A}^{\textrm{H}} \varvec{A} \in {\mathbb {C}}^{(N+1) \times (N+1)}$, $\varvec{Z} = \varvec{z}\varvec{z}^{\textrm{H}} \in {\mathbb {C}}^{(N+1) \times (N+1)}$, $\textrm{tr}(\varvec{W}\varvec{Z})$ is the trace of $\varvec{W}\varvec{Z}$ and $\varvec{A}^\textrm{H}$ is the conjugated transpose of $\varvec{A}$. Similar to ref.^102^, we can employ the relation for $\Vert \varvec{A}\varvec{z} \Vert _{2}^{2}$ to reformulate the optimisation problem that is stated in Eq. (10) as

12 $$\begin{aligned} \begin{aligned} \min \limits _{\varvec{Z}} \ &\textrm{tr} \left( \varvec{W} \varvec{Z} \right) \\ \quad \text {s.t.} \ &\qquad \ \ \varvec{Z} \succeq 0, \\&\quad \ \ \ Z_{i,i} = 1 \ \qquad \forall i \in \left\{ 1,2,\dots ,N+1 \right\} , \\&\textrm{rank}(\varvec{Z}) = 1, \end{aligned} \end{aligned}$$

where the positive semi-definiteness of $\varvec{Z}$ is ensured by $\varvec{Z} \succeq 0$. This problem is equivalent to Eq. (10), although the quadratic objective function is transformed into a linear one. If $\varvec{Z}^{*}$ is a feasible solution to Eq. (12), $\varvec{Z}^{*}$ can be written as $\varvec{Z}^{*} = \varvec{z}^{*} \left( \varvec{z}^{*}\right) ^\mathrm {{H}}$ where the condition $|z_i^{*} |= 1, 1 \le i \le N+1$ is fulfilled. This is taken into account by the constraints in Eq. (12). The idea of semidefinite programming is to omit the non-convex rank constraint, see ref.^57^, and to solve

13 $$\begin{aligned} \begin{aligned} \min \limits _{\varvec{Z}} \ &\textrm{tr} \left( \varvec{W} \varvec{Z} \right) \\ \quad \text {s.t.} \ &\ \ \ \, \varvec{Z} \succeq 0, \\&\ \, Z_{i,i} = 1 \ \qquad \forall i \in \left\{ 1,2,\dots ,N+1 \right\} . \end{aligned} \end{aligned}$$

Since Eq. (13), unlike the problems in Eqs. (8–10 , 12), is a convex problem, every local optimum $\varvec{Z}^{*}$ is also a global optimum. However, since $\textrm{rank}(\varvec{Z}^{*}) = 1$ cannot be ensured, $\varvec{Z}^{*}$ can only be regarded as an approximate solution to Eq. (12). Thus, a suitable $\varvec{z}^{*}$ based on $\varvec{Z}^{*}$ has to be determined that satisfies the constraints for $\varvec{Z}^{*}$ in Eq. (12) as good as possible. For this purpose, the eigenvector $\varvec{\lambda }$ of $\varvec{Z}^{*}$ with the highest eigenvalue $\lambda _\textrm{max}$ is used, as it is the optimal rank-one approximation of $\varvec{Z}^{*}$ in the $\mathcal {L}_2$-norm^103^. Subsequently, each element of $\varvec{\lambda }$ is normalised to obtain a feasible solution for the components

14 $$\begin{aligned} z_i^{*} = \frac{\lambda _i}{|\lambda _i |} \ \qquad \forall i \in \left\{ 1,2,\dots ,N+1 \right\} , \end{aligned}$$

which can be used with Eq. (11) to determine a feasible $\varvec{q}^{*}$ for Eq. (8). Finally, the key problem is solving Eq. (13) quickly and reliably. This semidefinite program is similar to the well-known *MaxCut* problem^104^, for which several algorithms were presented in recent years^57,59,62,73,84–87^. Among these, a block coordinate minimisation (BCM) with a low-rank factorisation of $\varvec{Z}$ is suitable for Eq. (13). The idea of low-rank factorisation, pioneered by Burer and Monteiro^60,61^, is to write $\varvec{Z}$ as $\varvec{Z} = \varvec{V}^\textrm{H} \varvec{V}$, where $\varvec{V} \in {\mathbb {C}}^{L \times (N+1)}$ is a low-rank matrix with $1 \le L \ll (N+1)$ By this choice, the condition $\varvec{Z} \succeq 0$ is fulfilled and the problem stated in Eq. (13) can be rewritten as

15 $$\begin{aligned} \begin{aligned}&\min \limits _{\varvec{V}} \ \textrm{tr} \left( \varvec{W} \varvec{V}^\textrm{H} \varvec{V} \right) &\\ \qquad \qquad & \text { s.t.} \ \Vert \varvec{V}_{:,j} \Vert _2 = 1 \ \qquad \forall j \in \left\{ 1,2,\dots ,N+1 \right\} , \end{aligned} \end{aligned}$$

where each column norm $\Vert \varvec{V}_{:,j} \Vert _2$ of $\varvec{V}$ must add up to one to ensure $Z_{i,i} = 1, i = 1,2,\dots ,N+1$. It is noteworthy that by this low-rank factorisation, the convex problem in Eq. (13) is transformed back into an unfavourable non-convex problem in Eq. (15), which appears to be a step back on first sight. However, it has been shown that the non-convexity of Eq. (15) does not cause any major problems in practice^59,60,62,105^. In contrast, the transformation $\varvec{Z} = \varvec{V}^\textrm{H} \varvec{V}$ significantly reduces the number of variables from $(N+1)^2$ in $\varvec{Z}$ to $L (N+1)$ in $\varvec{V}$, since *L* is chosen as $1 \le L \ll (N+1)$. Barvinok^106^ and Pataki^107^ proved that if Eq. (15) has an optimum and $N+1$ constraints, it admits optimal results of rank *L* such that $L(L + 1)/2 \le (N+1)$^62,85^.

Using this result, it was shown in refs.^60,61,108^ that for $L > \sqrt{2(N+1)}$, the optimal solution $\varvec{V}^{*}$ to Eq. (15) can recover $\varvec{Z}^{*}$ for Eq. (13)^59,62^. However, it is also possible to select even smaller values for *L* that are suitable for the individual application in order to improve the execution speed, since storage requirements and the costs for matrix-vector multiplications would drop significantly from $\mathcal {O}\left( (N+1)^2\right)$ in Eq. (13) to $\mathcal {O}\left( L(N+1)\right)$ in Eq. (15)^85^. Although Eq. (15) is a non-convex problem^59^, it was shown in ref.^62^ that BCM is able to converge to a solution with accuracy $\epsilon$ in time $\mathcal {O}\left( \frac{1}{\epsilon }\right)$ for $L > \sqrt{(2(N+1))}$, see also ref.^85^. The idea of BCM is to optimise $\textrm{tr} \left( \varvec{W} \varvec{V}^\textrm{H} \varvec{V} \right)$ with respect to one column $\varvec{V}_{:,j}$ and to consider all other columns of $\varvec{V}$ as constant. Thus, BCM solves the problem

16 $$\begin{aligned} \begin{aligned}&\min \limits _{\varvec{V}_{:,j}} \ \textrm{tr} \left( \varvec{W} \varvec{V}^\textrm{H} \varvec{V} \right) \\&\text { s.t.} \ \Vert \varvec{V}_{:,j} \Vert _2 = 1 \end{aligned} \end{aligned}$$

in each iteration, which has an analytic solution. A closer look at $\textrm{tr} \left( \varvec{W} \varvec{V}^\textrm{H} \varvec{V} \right)$ and the constraints $\varvec{V}_{:,j}^\textrm{H} \varvec{V}_{:,j} = 1, j \in \left\{ 1,2,\dots ,N+1 \right\}$ reveals that the diagonal terms of $\varvec{W}$ do not affect the optimal result of Eq. (16)^59^. Consequently, the overall performance of the algorithm can be improved by optimising the following objective function instead, where these constant terms are omitted:

17 $$\begin{aligned}&\phantom {=} \ \ \textrm{tr} \left( \varvec{W} \varvec{V}^\textrm{H} \varvec{V} \right)-\sum _{\begin{array}{c} i=1 \\ i \ne j \end{array}}^{N+1} \sum _{\begin{array}{c} l=1 \\ l \ne j \end{array}}^{N+1} W_{i,l} \varvec{V}_{:,i}^\textrm{H} \varvec{V}_{:,l} - W_{j,j} \varvec{V}_{:,j}^\textrm{H} \varvec{V}_{:,j} \nonumber \\&= \varvec{V}^\textrm{H}_{:,j} \underbrace{ \left( \sum _{\begin{array}{c} i=1 \\ i \ne j \end{array}}^{N+1} W_{i,j} \varvec{V}_{:,i} \right) }_{:= \, \varvec{g}_{j}} + \underbrace{ \left( \sum _{\begin{array}{c} i=1 \\ i \ne j \end{array}}^{N+1} W_{j,i} \varvec{V}_{:,i}^\textrm{H} \right) }_{:= \, \varvec{g}_{j}^\textrm{H}} \varvec{V}_{:,j} \nonumber \\&= 2 \Re \left\{ \varvec{g}_j^\textrm{H} \varvec{V}_{:,j} \right\} , \text {where} \ \Re \left\{ a + \textrm{ib} \right\} = a. \end{aligned}$$

Hence, minimising Eq. (16) is, up to a constant, equivalent to the problem

18 $$\begin{aligned} \begin{aligned}&\min \limits _{\varvec{V}_{:,j}} \ 2 \Re \left\{ \varvec{g}_j^\textrm{H} \varvec{V}_{:,j} \right\} \\&\text { s.t.} \ \Vert \varvec{V}_{:,j} \Vert _2 = 1. \end{aligned} \end{aligned}$$

Similar to refs.^59,62^, the unique solution to the problem in Eq. (18) is

19 $$\begin{aligned} \varvec{V}_{:,j}^{*} = -\left( {\varvec{g}_j}\, / \,{\Vert \varvec{g}_j \Vert _2} \right) . \end{aligned}$$

Inserting Eq. (19) in Eq. (18) results in

20 $$\begin{aligned} 2 \Re \left\{ \varvec{g}_j^\textrm{H} \varvec{V}_{:,j}^{*} \right\} = 2 \Re \left\{ -\varvec{g}_j^\textrm{H} \left( \varvec{g}_j\,/\,\Vert \varvec{g}_j \Vert _2 \right) \right\} = -2 \Vert \varvec{g}_j \Vert _2. \end{aligned}$$

**Algorithm 1:**
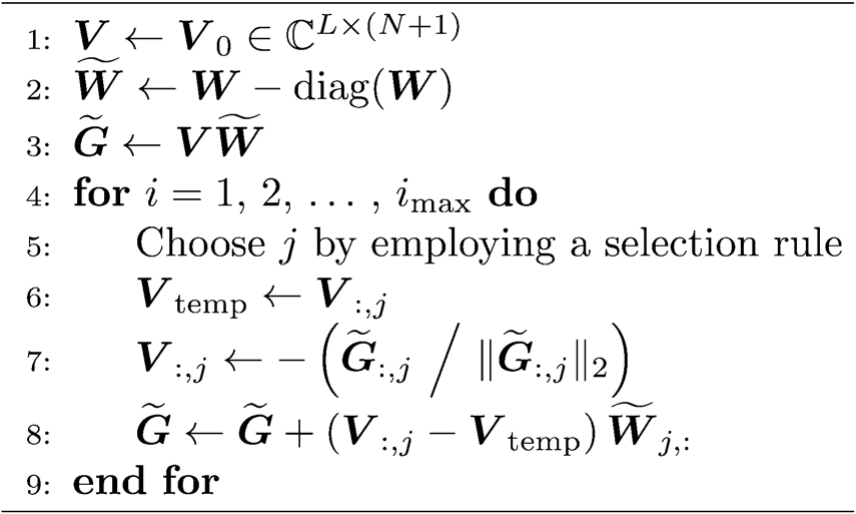
Block coordinate minimisation.

By subtracting Eq. (20) from Eq. (18), the objective function is reduced by

21 $$\begin{aligned} 2 \left( \Re \left\{ \varvec{g}_j^\textrm{H} \varvec{V}_{:,j} \right\} + \Vert \varvec{g}_j \Vert _2 \right) . \end{aligned}$$

Based on the obtained results, we can now state the implementation of the BCM algorithm in Algorithm 1, where we took hints given in^62^ into account. For example, by caching the vectors $\varvec{g}_j$ in a matrix $\widetilde{\varvec{G}}$ to keep the changes after each step, the total number of operations in one iteration can be reduced to $\mathcal {O}\left( L(N+1)\right)$^85^. To select the column index *j* in line 5, we implemented four different strategies to investigate their performance in solving Eq. (15):

- *Uniform sampling*^62^: Select *j* randomly with probability $p_j = {1}\,/\,{(N+1)}$.
- *Importance sampling*^62^: Select *j* with $p_j = \Vert \widetilde{\varvec{G}}_{:,j}\Vert _2\,\big /\,\sum _{k=1}^{N+1} \Vert \widetilde{\varvec{G}}_{:,k}\Vert _2$.
- *Cyclic update*^59,87^: Update all columns of $\widetilde{\varvec{G}}$ cyclically in ascending order.
- *Greedy coordinate selection*^62^: Evaluate
  22 $$\begin{aligned} \mathop {{{\,\textrm{argmax}\,}}}\limits _{1 \le j \le N+1} 2 \left( \Re \left\{ \widetilde{\varvec{G}}^\textrm{H}_{:,j} \varvec{V}_{:,j} \right\} + \Vert \widetilde{\varvec{G}}_{:,j} \Vert _2 \right) \end{aligned}$$
  and select that column *j* leading to a maximal improvement of $\textrm{tr} \left( \varvec{W} \varvec{V}^\textrm{H} \varvec{V} \right)$. The BCM algorithm terminates after $i_\textrm{max}$ column updates. This value can be set by the user to meet requirements that may be imposed in terms of the quality of the resulting sound pressure field and the real-time capability of the algorithm. In addition to this hard criterion, soft threshold-based termination criteria like $2 \left( \Re \left\{ \varvec{g}_j^\textrm{H} \varvec{V}_{:,j} \right\} + \Vert \varvec{g}_j \Vert _2 \right) \ge \zeta$ to enforce a minimum convergence rate $\zeta \in {\mathbb {R}}^{+}$ of BCM or $\textrm{tr} \left( \varvec{W} \varvec{V}^\textrm{H} \varvec{V} \right) \le \xi , \ \xi \in {\mathbb {R}}$, to ensure a certain quality of the reconstructed sound pressure field can also be considered. Finally, it is worth noting that Algorithm 1 requires $\Vert \widetilde{\varvec{G}}_{:,j}\Vert _2 \ne 0$ for each selected column *j* in each step. Although the case $\Vert \widetilde{\varvec{G}}_{:,j}\Vert _2 = 0$ did not occur in any of our investigations, we cannot completely exclude its occurrence. Hence, as proposed in Sec. 3.1 in ref.^86^ for the strategy *cyclic update*, no column update is performed in this case. If another selection strategy is used, we suggest to take the second most appropriate element according to the chosen selection strategy or to simply heuristically pick another column.

### Characterising sound pressure fields

To evaluate the reconstruction quality with respect to a given sound pressure field, it is necessary to formulate an objective based on the present application. As acoustic holograms and levitation techniques are widely used in numerous and heterogeneous applications, we introduce three separate metrics. If the focus lies on the exact reconstruction of pressure amplitudes at positions $\varvec{r}_i \in \mathcal {M}$, as in case of haptic interfaces^42^ or acoustic holograms^36^, it is useful to calculate the relative errors

23 $$\begin{aligned} e_\textrm{SDP}&= \frac{\sqrt{\textrm{tr} \left( \varvec{W} \varvec{V}^\textrm{H} \varvec{V} \right) }}{\Vert {\varvec{p}} \Vert _2} \quad \text {and} \quad e_\textrm{p,rel} = \frac{\left\| \varvec{G} \varvec{q}^{*} - \varvec{p}\right\| _2}{\Vert {\varvec{p}} \Vert _2}. \end{aligned}$$

In Eq. (23), $e_\textrm{SDP}$ compares the solution of the SDP in Eq. (13) with the exact pressure, whereas $e_\textrm{p,rel}$ describes the actual relative pressure error after rounding the optimal solution $\varvec{Z}^{*}$ to rank one to obtain $\varvec{z}^{*}$ and $\varvec{q}^{*}$, see also Eqs. (11, 14). However, these metrics are of limited use for the contactless manipulation of objects, where the force distribution of the acoustic trap is more significant. Therefore, for a given $\varvec{q}^{*}$, we evaluate the displacement error

24 $$\begin{aligned} e_\textrm{pos} = \left\| \varvec{r}_\textrm{eq} - \varvec{r}_\textrm{trap} \right\| _2 \end{aligned}$$

that is determined by the norm between the position $\varvec{r}_\textrm{trap}$, at which the pressure field of the acoustic trap was reconstructed, and the actual equilibrium position $\varvec{r}_\textrm{eq}$ of the object inside the trap. To obtain $\varvec{r}_\textrm{eq}$, we employed the Levenberg-Marquardt algorithm^77,78^ in Algorithm 2, which interpolates between the gradient descent method and the Gauss-Newton algorithm, whose individual influence can be adjusted by the damping parameter $\alpha$. First, starting from $\varvec{r}_{\textrm{eq},0} = \varvec{r}_\textrm{trap}$, the resulting force $\varvec{F}_\textrm{res}$ was determined in each iteration. According to line 7 in Algorithm 2, this force can be calculated from the sum of the acoustic radiation force $\varvec{F}_\textrm{rad}\left( \varvec{r}_{\textrm{eq},{i-1}} \right)$ and the gravitational force $\varvec{F}_g = -m \varvec{g}$ acting on the levitated object with a mass *m* at $\varvec{r}_{\textrm{eq},{i-1}},$ where $\varvec{g} = g_\textrm{x} \varvec{e}_\textrm{x} + g_\textrm{y} \varvec{e}_\textrm{y} + g_\textrm{z} \varvec{e}_\textrm{z}$ denotes the vector of gravitational acceleration. To determine $\varvec{F}\left( \varvec{r}_{\textrm{eq},{i-1}} \right),$ appropriate force models such as Gor’kov^63^, Acoustokinetics^65^, the boundary element method^15^ as well as approaches based on spherical harmonics^16,17^ or the angular spectrum method^64^ can be used. In this case, we employed the equations stated in^17^ to calculate the acoustic radiation force on the Mie sphere. Subsequently, the position shift $\Delta \varvec{r}$ is calculated in line 11 in Algorithm 2, where $\varvec{I}^{3 \times 3} \in {\mathbb {R}}^{3 \times 3}$ is the identity matrix, $\nabla \varvec{F}\left( \varvec{r}_{\textrm{eq},{i-1}} \right)$ is the Jacobian matrix of the acoustic radiation force $\varvec{F}$ at $\varvec{r}_{\textrm{eq},{i-1}}$, and $\varvec{A}^{-1}$ denotes the inverse of $\varvec{A}$. Finally, $\Delta \varvec{r}$ is used to update the current equilibrium position $\varvec{r}_{\textrm{eq},{i-1}}$ in line 12. The iterative search in Algorithm 2 is executed until either a possible equilibrium position satisfies the abort criterion $\varvec{F}_\textrm{res}^\top \varvec{F}_\textrm{res} \le F_\textrm{tol}$ in line 8, the maximum number of iterations $i_\textrm{max}$ is exceeded, or the domain of attraction defined by $r_\textrm{tol}$ (see line 4) is left. This occurs if the acoustic trap had an unstable force distribution.

**Algorithm 2:**
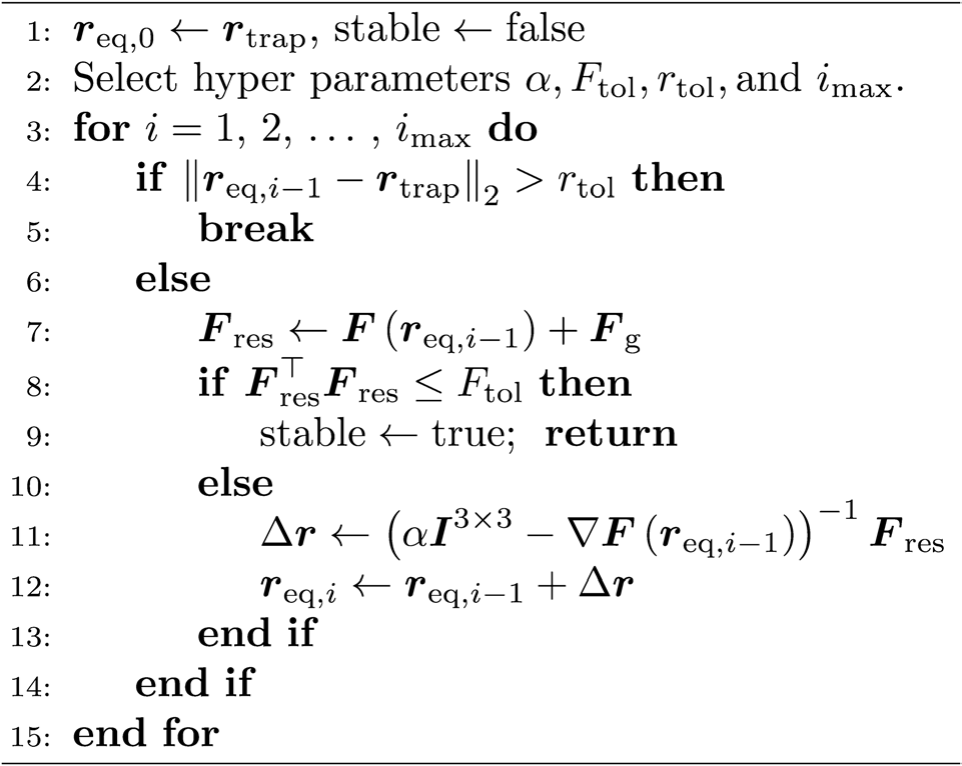
Determine equilibrium position $\varvec{r}_\textrm{eq}$.

### Shifting acoustic pressure fields

Algorithm 3 presents our approach to shift a desired sound pressure field $\widetilde{\varvec{p}} \in {\mathbb {C}}^{P \times 1}$ at $\mathcal {P} = \left\{ \varvec{r}_1, \varvec{r}_2, \dots , \varvec{r}_P \right\}$ to a new position or orientation, taking into account the activation $\varvec{q} \in {\mathbb {C}}^{N \times 1}$ of a transducer array with *N* elements. At first, important features of the sound field are extracted by means of a point grid $\mathcal {M} = \left\{ \varvec{r}_1, \varvec{r}_2, \dots , \varvec{r}_M \right\}$ with $M \ll P$. Picking $\varvec{r}_i \in \mathcal {M}$ can be done using a 2D Gaussian grid, one of the spherical grids in Fig. 4, or simply in a heuristic manner. After calculating the reduced representation $\varvec{p}_0$ of the sound field $\widetilde{\varvec{p}}$ by using Eqs. (4-6), the whole grid $\mathcal {M}$ is translated or respectively rotated to a different pose. This is accomplished by applying a corresponding transformation rule, e.g., a translation by a vector $\varvec{v} \in {\mathbb {R}}^{3 \times 1}$ or a rotation according to a given matrix $\varvec{R} \in {\mathbb {R}}^{3 \times 3}$, to each point $\varvec{r}_i \in \mathcal {M}$ in order to obtain the corresponding positions $\varvec{s}_i \in \mathcal {N}$ of the new grid $\mathcal {N} = \left\{ \varvec{s}_1, \varvec{s}_2, \dots , \varvec{s}_M \right\}$. Subsequently, by using Eqs. (4-6), the propagator matrix $\varvec{G}_1 \in {\mathbb {R}}^{M \times N}$ can be calculated for $\mathcal {N}$, which can be used together with $\varvec{p}_0$ to obtain $\varvec{W} \in {\mathbb {C}}^{(N+1) \times (N+1)}$.

**Algorithm 3:**
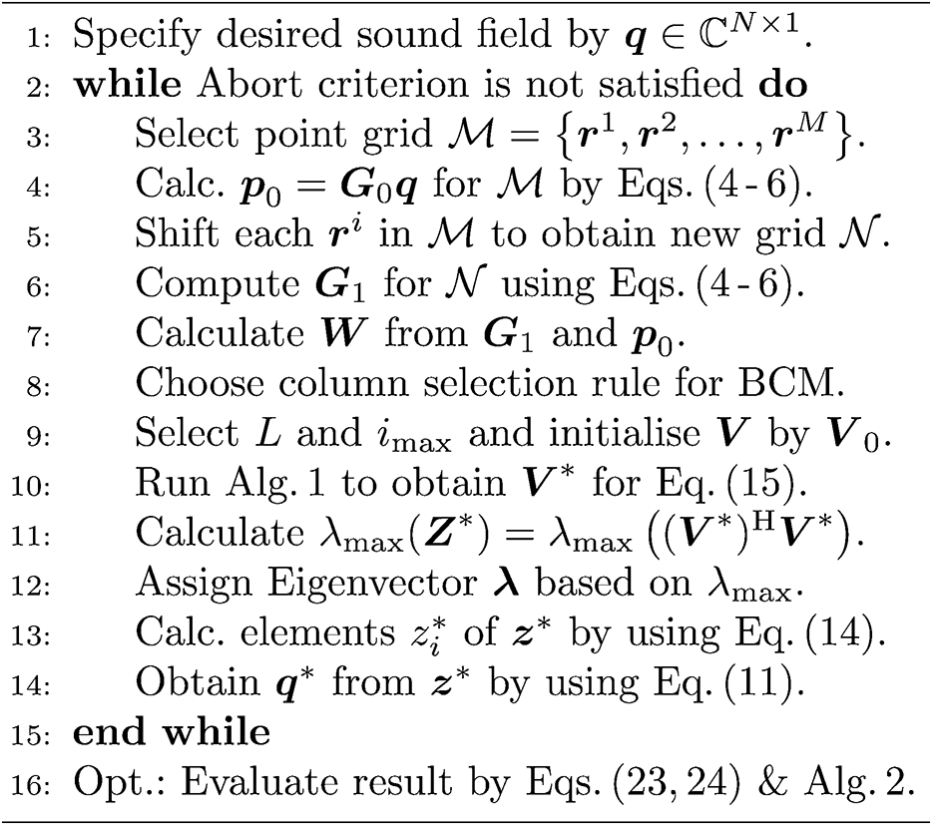
Shifting acoustic pressure fields.

After choosing a suitable column selection rule for BCM as well as appropriate hyper parameters $\varvec{V}_0$, *L*, and $i_\textrm{max}$, Algorithm 1 is executed to obtain an optimal solution $\varvec{V}^{*}$ for the semidefinite program in Eq. (15). Due to the low-rank factorisation of $\varvec{Z}$, $\varvec{Z}^{*}$ is defined as $\varvec{Z}^{*} = (\varvec{V}^{*})^\textrm{H} \varvec{V}^{*}$, which makes it possible to obtain the optimal rank-one approximation of $\varvec{Z}^{*}$ by selecting the Eigenvector $\varvec{\lambda }$ of $(\varvec{V}^{*})^\textrm{H} \varvec{V}^{*}$ with the highest eigenvalue $\lambda _\textrm{max}$. Finally, the optimal activation $\varvec{q}^{*}$ for the phased array can be calculated from $\varvec{\lambda }$ by employing Eqs. (14) and (11). To evaluate $\varvec{q}^{*}$, one can employ Eqs. (23, 24) together with Algorithm 2. In addition, other criteria, such as the execution time of Algorithm 3, with respect to a required real-time capability in the present application, could also be taken into account. If $\varvec{q}^{*}$ is feasible, Algorithm 3 can be terminated. Otherwise, the quantity *M* of points in $\mathcal {M}$ or their positions $\varvec{r}_i \in \mathcal {M}$ may have to be changed. Furthermore, one can also try a modification of the parameters $\varvec{V}_0$, *L*, and $i_\textrm{max}$ before executing Algorithm 1 again.

### Real-time capability

To show the real-time capability of the proposed method, we investigated the run-time cost of Algorithm 1 when run as part of Algorithm 3. Before the start of the iteration, the matrix $\widetilde{{\varvec{W}}}$ is computed, which in turn is constructed as ${\varvec{W}} = \varvec{A}^\textrm{H}\varvec{A}$ from the matrix $\varvec{G}$ of Eq. (6) and one additional column. For *N* transducers and *M* control points, computing $\varvec{G}$ involves ${\mathcal {O}}(N M)$ arithmetic operations and memory accesses. The matrix-matrix multiplication to compute $\varvec{W}$ involves ${\mathcal {O}}(N^2 M)$ arithmetic operations. Note that in the limit of very large *N* and *M*, the complexity can be lowered somewhat, see refs.^109^^,^^110^. The final step of the setup phase, line 3 of Algorithm 1, is the computation of $\widetilde{\varvec{G}}$, which involves ${\mathcal {O}}(L N^2)$ arithmetic operations in case *L* vectors are kept in the matrix $\varvec{V}$. All other operations in the setup phase purely work on vectors and are of cost ${\mathcal {O}}(N)$ and $\mathcal O(M)$, respectively. Each iteration of the loop in Algorithm 1 involves the identification of a suitable index *j* for the update, which is ${\mathcal {O}}(L N)$ for importance sampling and the greedy algorithm and ${\mathcal {O}}(1)$ for the uniform sampling or cyclic update. Lines $6-8$ in the algorithm update the matrix column ${\varvec{V}}_{:,j}$ at ${\mathcal {O}}(L)$ operations and eventually update $\widetilde{\varvec{G}}$ at ${\mathcal {O}}(LN)$ arithmetic operations. Overall, the complexity of the optimisation loop then becomes ${\mathcal {O}}(i_{\max } L N).$ The main advantage of the algorithm is the linear complexity per iteration, since our algorithm only works with a column of the matrix $\widetilde{\varvec{W}}$ at a time to compute the update to $\widetilde{\varvec{G}}$. By comparison, many algorithms from the literature, such as the one given in ref.^34^, involve a dense matrix-vector product in inner iterations, albeit with smaller matrix dimensions *M*.

Regarding the actual run time when executed with an optimised code on contemporary hardware, we assume that $i_\textrm{max}\sim N$ (see Fig. 5), which implies a cost for the optimisation loop of ${\mathcal {O}}(L N^2)$. Then, the following three observations can be made: First, the formally most expensive operation, the matrix-matrix multiplication $\varvec{A}^\textrm{H} \varvec{A}$, is of BLAS-3 type and can be highly optimised on modern CPU or GPU processors due to the high operational intensity^97,111^. In addition, the resulting matrix is Hermitian, such that only the upper triangular part needs to be computed. Taking as an example $N = 1024$, $M=26$ for a single core of an AMD Ryzen 7 Pro 7840U core running at $5.1\,\textrm{GHz}$, which offers an arithmetic throughput of $81.6\,\text {Gflops}\,\textrm{s}^{-1}$, the ideal run time would be $1025^2 \cdot 26 \cdot 8 / 2 / ({81.6}\textrm{e}{9}) = 1.34\,\textrm{ms}$. Note that the measured time in Table 2 is $2.06\,\textrm{ms}$. Second, since *M* and *N* take relatively low values in practice, the proportionality constants and especially the evaluation of the Bessel function of first kind for Eq. (5) can be significant. However, for the chosen range of arguments, a Taylor expansion with $10-15$ terms typically suffices, making the evaluation cost lower than for the matrix-matrix multiplication for $N>100$. Third, the vector updates and column selection in the optimisation iteration are akin to BLAS-1 type (operations on vectors). These have low arithmetic intensities and are limited by the memory access. Since the values of *N* and *L* are often very low, the vector ${\varvec{G}}$ can fit into fast level-1 or level-2 caches, making the access to $\widetilde{{\varvec{W}}}$ the only expensive step at ${\mathcal {O}}(N)$ operations.

### Polynomial regression

The goal of this polynomial regression is to obtain the parameters $\varvec{A} \in {\mathbb {R}}^{\alpha \times 3}$ and $\varvec{B} \in {\mathbb {R}}^{\beta \times 3}$ with $\alpha ,\beta \in {\mathbb {N}}^{+}$ of the models $\Delta \varvec{r} = \varvec{P}(\varvec{F}_\textrm{res},\varvec{B})$ and $\varvec{F}_\textrm{res} = \varvec{P}(\Delta {\varvec{r}},\varvec{A})$ (see Fig. 2) in such way that the conditions $\Delta \varvec{r} \overset{!}{=} \varvec{P}(\varvec{F}_\textrm{res},\varvec{B})$ and $\varvec{F}_\textrm{res} \overset{!}{=} \varvec{P}(\Delta {\varvec{r}},\varvec{A})$ are fulfilled. To train these models $\varvec{P}$ of multivariate polynomials, a look-up table (LUT) is created based on an activation $\varvec{\phi }_\textrm{opt}$ that ensures a stable trapping of the object at $\varvec{r}_\textrm{opt}$ by the PAT. Each data tuple $(\Delta \varvec{r}_i, \varvec{F}_{\textrm{res},i})$ of the LUT with $i \in \left\{ 1,2,\dots ,P\right\}$ is comprised of distances $$\Delta \varvec{r}_i = \begin{pmatrix} \Delta r_{\textrm{x},i}&\Delta r_{\textrm{y},i}&\Delta r_{\textrm{z},i} \end{pmatrix}^\top = \varvec{r}_i - \varvec{r}_\textrm{opt}$$ and resulting forces $\varvec{F}_{\textrm{res},{i}} = \varvec{F}_{\textrm{rad},{i}}(\Delta \varvec{r}_i) + \varvec{F}_\textrm{g}$. These forces $\varvec{F}_\textrm{res}(\Delta \varvec{r}_i)$ will be exerted on the object due to trap displacements by $\Delta \varvec{r}_i$. Depending on the object and the target accuracy of the result, various methods can be applied to calculate $\varvec{F}_{\textrm{rad},{i}}(\Delta \varvec{r}_i)$^15–17,63–65^.

In both cases, $\varvec{A}$ or $\varvec{B}$ will be considered as optimal when they minimise the resulting Euclidean norm of the errors between the given LUT data and the corresponding predicted values. Denoting $\varvec{P}(\varvec{X})$ as general model of multivariate polynomials with total degrees $\textrm{Grad}(\varvec{P}) = \delta$, an optimal result can be obtained by solving the following optimisation problem:

25 $$\begin{aligned} \min \limits _{\varvec{P}} \left\| \varvec{Y} - \varvec{P}(\varvec{X}) \right\| _\textrm{F} \ \text {with} \ \textrm{Grad}(\varvec{P}) = \delta . \end{aligned}$$

In Eq. (25), $\left\| \varvec{C}\right\| _\textrm{F} = \sqrt{\sum _{i,j} C_{i,j}^2}$ denotes the Frobenius norm, given by the square root of the sum of the squares of all elements $C_{i,j}$ of the matrix $\varvec{C}$. Furthermore, the input $\varvec{X}$ and the output $\varvec{Y}$ are obtained by concatenating the corresponding data points of the LUT. In case of $\varvec{F}_\textrm{res} = \varvec{P}(\Delta {\varvec{r}},\varvec{A})$, the matrices $\varvec{X} \in {\mathbb {R}}^{P \times 3}$ and $\varvec{Y} \in {\mathbb {R}}^{P \times 3}$ are given by $$\varvec{X} = \begin{pmatrix} \Delta \varvec{r}_1\Delta \varvec{r}_2\dots\Delta \varvec{r}_P \end{pmatrix}^\top$$ and $$\varvec{Y} = \begin{pmatrix} \varvec{F}_{\textrm{res},1}&\varvec{F}_{\textrm{res},2}&\dots&\varvec{F}_{\textrm{res},P} \end{pmatrix}^\top$$, whereas in case of $$\varvec{Y} = \begin{pmatrix} \varvec{F}_{\textrm{res},1}\varvec{F}_{\textrm{res},2}\dots\varvec{F}_{\textrm{res},P} \end{pmatrix}^\top$$, $\varvec{X}$ and $\varvec{Y}$ are swapped. Since both vectors $\varvec{F}_\textrm{res}(\Delta \varvec{r})$ and $\Delta \varvec{r}$ are defined in Cartesian space, it is necessary to setup corresponding polynomials in (*x*, *y*, *z*) with coefficients $h_{l,m,n}$ and total degree $\delta \in {\mathbb {N}}^{+}$:

26 $$\begin{aligned} p(x,y,z) = \sum _{l+m+n \le \delta } h_{l,m,n} x^l y^m z^n. \end{aligned}$$

As $\Delta \varvec{r}$ and $\varvec{F}_\textrm{res}(\Delta \varvec{r})$ have three spatial components, polynomials $p_{i}(x,y,z)$ with coefficients $h_{i,l,m,n}$, $i \in \left\{ \textrm{x},\textrm{y},\textrm{z}\right\}$, are needed in total for each case. Consequently, the evaluation of the model $\varvec{P}(\varvec{X})$ of the three multivariate polynomials can be written in matrix notation as follows:

27 $$\begin{aligned} \varvec{P}(\varvec{X}) = \widehat{\varvec{Y}} = \underbrace{ \begin{pmatrix} 1 & x_1 & y_1 & z_1 & \ldots \\ 1 & x_2 & y_2 & z_2 & \ldots \\ \vdots & \vdots & \vdots & \vdots & \ddots \\ 1 & x_P & y_P & z_P & \ldots \end{pmatrix} }_{\varvec{M}} \underbrace{ \begin{pmatrix} h_{\textrm{x},{0,0,0}} & h_{\textrm{y},{0,0,0}} & h_{\textrm{z},{0,0,0}} \\ h_{\textrm{x},{1,0,0}} & h_{\textrm{y},{1,0,0}} & h_{\textrm{z},{1,0,0}} \\ h_{\textrm{x},{0,1,0}} & h_{\textrm{y},{0,1,0}} & h_{\textrm{z},{0,1,0}} \\ h_{\textrm{x},{0,0,1}} & h_{\textrm{y},{0,0,1}} & h_{\textrm{z},{0,0,1}} \\ \vdots & \vdots & \vdots \end{pmatrix} }_{\varvec{H}} \end{aligned}$$

In Eq. (27), $\widehat{\varvec{Y}} \in {\mathbb {R}}^{P \times 3}$ denotes the prediction of $\varvec{P}(\varvec{X})$, $\varvec{M} \in {\mathbb {R}}^{P \times \gamma }$ is the matrix of monomials and the matrix $\varvec{H} \in {\mathbb {R}}^{\gamma \times 3}$ is comprised of all polynomial coefficients. Here $\gamma = \alpha$ for $\varvec{F}_\textrm{res} = \varvec{P}(\Delta {\varvec{r}},\varvec{A})$ and $\gamma = \beta$ for $\Delta \varvec{r} = \varvec{P}(\varvec{F}_\textrm{res},\varvec{B})$. Inserting Eq. (27) in Eq. (25) yields

28 $$\begin{aligned} \min \limits _{\varvec{H}} \left\| \varvec{Y} - \varvec{M}\varvec{H} \right\| _\textrm{F}. \end{aligned}$$

The modified optimisation problem stated in Eq. (28) has an analytical solution, namely $\varvec{H}^{*} = \varvec{M}^{\dagger } \varvec{Y}$, with $\varvec{M}^{\dagger } = (\varvec{M}^\top \varvec{M})^{-1}\varvec{M}^{\top }$ denoting the pseudo-inverse of the matrix $\varvec{M}$. Thus, the parameter sets $\varvec{A}$ and $\varvec{B}$ can simply be obtained by solving Eq. (28) with the corresponding matrices $\varvec{M}$ and $\varvec{Y}$.

### Training and validation of the polynomial models

In the previous section Polynomial Regression, the necessary steps were explained to obtain two polynomial models $\widehat{\varvec{F}}_\textrm{res} = \varvec{P}(\Delta \varvec{r},\varvec{A})$ and $\Delta \widehat{\varvec{r}} = \varvec{P}(\varvec{F}_\textrm{res},\varvec{B})$ with parameter matrices $\varvec{A} \in {\mathbb {R}}^{\alpha \times 3}$ and $\varvec{B} \in {\mathbb {R}}^{\beta \times 3}$ based on a LUT comprised of tuples $(\Delta \varvec{r}_i,\varvec{F}_{\textrm{res},i}(\Delta \varvec{r}_i))$ with $i \in \left\{ 1,2,\dots ,P\right\}$. To train our models and validate their quality, we defined a cubic volume centred at $\varvec{r}_\textrm{opt}$ with edge length $2 R_\lambda$:

29 $$\begin{aligned} \mathcal {V}(\varvec{r}_\textrm{opt},R_\lambda ) = \left\{ \varvec{r}_i \in {\mathbb {R}}^3 \,\big \vert \,\vert {r}_{j,i} - {r}_{j,\textrm{opt}} \vert \le R_\lambda , R_\lambda \in {\mathbb {R}}^{+}, j \in \left\{ \textrm{x,y,z}\right\} \right\} . \end{aligned}$$

Using an activation $\varvec{\phi }_\textrm{opt}$ for the PAT that stably suspends an object at $\varvec{r}_\textrm{opt}$, the samples for the LUT were obtained from $\mathcal {V}(\varvec{r}_\textrm{opt},R_\lambda )$. For the polynomial models, we assumed that the radiation force distribution around a given trap centre $\varvec{r}_\textrm{trap}$ is constant in a corresponding volume $\mathcal {V}(\varvec{r}_\textrm{trap},R_\lambda )$. As a consequence, the radiation force distribution can be considered as invariant with regard to trap shifts in a certain workspace $\mathcal {W} = \mathcal {V}(\varvec{r}_\textrm{opt},R_\Omega )$, compare with Eq. (29), with edge length $2R_\Omega \gg 2R_\lambda$. This means that when a trap is shifted from $\varvec{r}_\textrm{opt}$ to $\varvec{r}_\textrm{trap},$ the resulting force $\varvec{F}_\textrm{res}$ shall depend exclusively on the relative distance $\Delta \varvec{r}$ between object and actual trap centre, but not on the absolute position of the trap centre in the workspace $\mathcal {W}$:

30 $$\begin{aligned} \varvec{F}_\textrm{res}\left( \Delta \varvec{r},\varvec{r}_\textrm{opt} \right) \overset{!}{=} \varvec{F}_\textrm{res}\left( \Delta \varvec{r},\varvec{r}_\textrm{trap} \right) \approx \varvec{F}_\textrm{res}\left( \Delta \varvec{r} \right) \end{aligned}$$

To validate this assumption, we defined a volume $\mathcal {V}\left( \varvec{r}_\textrm{opt},R_\Omega - R_\lambda \right)$ in which the prediction quality of the polynomial models shall be evaluated. After shifting a trap defined by $\varvec{\phi }_\textrm{opt}$ at $\varvec{r}_\textrm{opt}$ to a position $\varvec{r}_\textrm{trap}$ by Algorithm 3, we can compare the force $\varvec{F}_{\textrm{res},j} \left( \Delta \varvec{r}_j,\varvec{r}_\textrm{trap}\right)$ calculated with approaches like^15–17^ with the prediction $\widehat{\varvec{F}}_{\textrm{res},j}$ by the polynomial model $\varvec{P}(\Delta \varvec{r}_j,\varvec{A})$ for displacements $\Delta \varvec{r}_j$ at positions $\varvec{r}_j = \Delta \varvec{r}_j + \varvec{r}_\textrm{trap}$, $j = \left\{ 1,2,\dots ,J\right\}$ in a corresponding volume $\mathcal {V}\left( \varvec{r}_\textrm{trap},R_\lambda \right)$. For each of these *J* positions, we can define an error $e_j = \Vert \widehat{\varvec{F}}_{\textrm{res},j} -\varvec{F}_{\textrm{res},j} \Vert _2$ and store them in a corresponding set $\mathcal {E}(\varvec{r}_\textrm{trap})$. Finally, to quantify the prediction quality of the polynomial model at $\varvec{r}_\textrm{trap}$, we use the maximum and mean error as metrics:

31 $$\begin{aligned} e_\textrm{max}\left( \varvec{r}_\textrm{trap} \right)&= \max _{{e_j \in \mathcal {E}(\varvec{r}_\textrm{trap})}} e_j \end{aligned}$$

32 $$\begin{aligned} e_\textrm{mean}\left( \varvec{r}_\textrm{trap} \right)&= \frac{1}{J}\sum _{j = 1}^{J} e_j, \quad e_j \in \mathcal {E}(\varvec{r}_\textrm{trap}) \end{aligned}$$

Following this idea, we can define similar metrics for the evaluation of $\Delta \widehat{\varvec{r}} = \varvec{P}(\varvec{F}_\textrm{res},\varvec{B})$ to quantify the prediction quality of $\Delta \widehat{\varvec{r}}$. Finally, this workflow is summarised in Algorithm 4 for the polynomial model $\widehat{\varvec{F}}_\textrm{res} = \varvec{P}(\Delta \varvec{r},\varvec{A})$.

**Algorithm 4:**
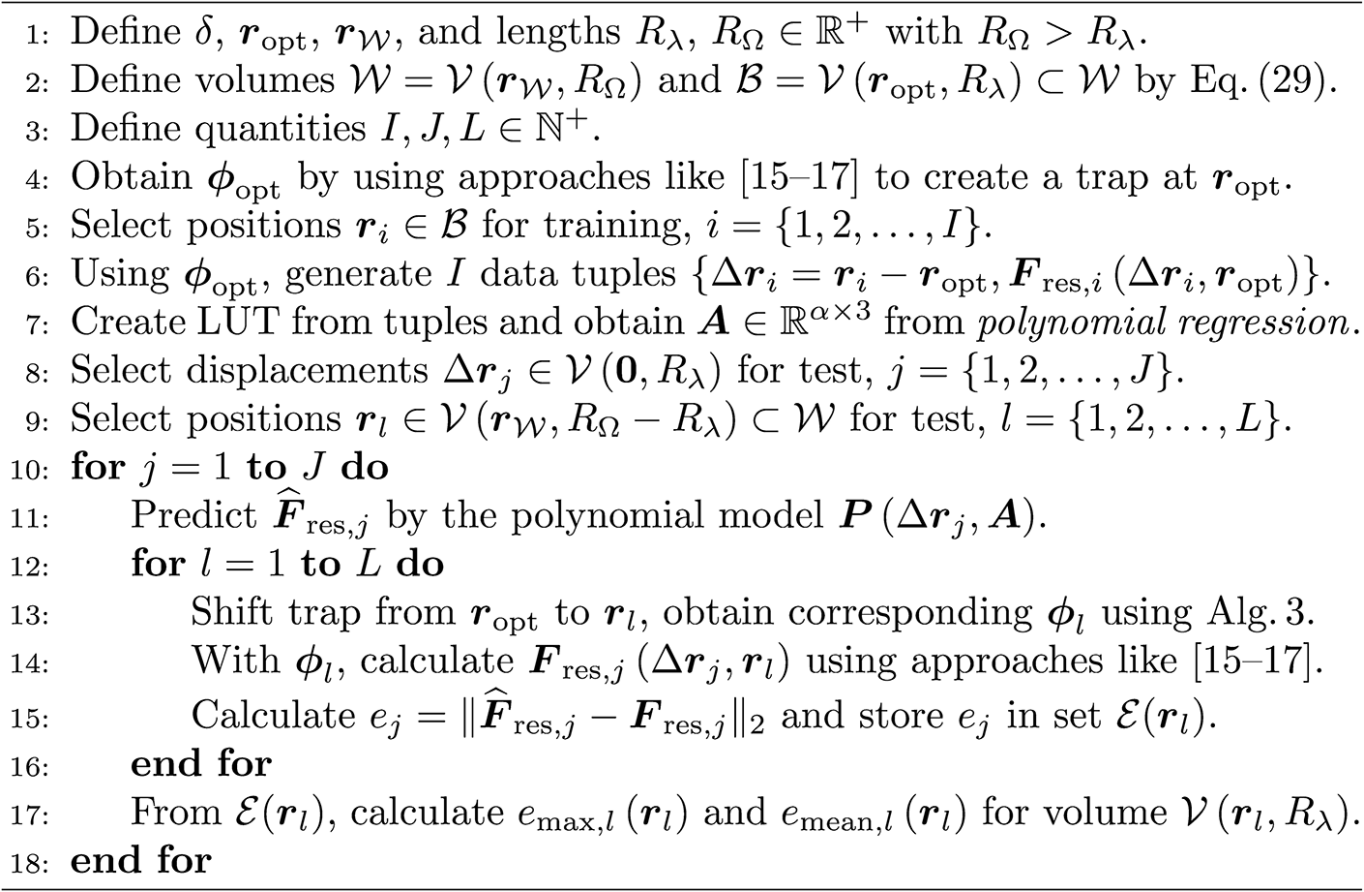
Validation of $$\widehat{\varvec{F}}_\textrm{res} = \varvec{P}(\Delta \varvec{r},\varvec{A})$$.

### Dynamic model of the Mie sphere

The motion of the Mie sphere through quiescent air can be described by the non-linear state space model

33 $$\begin{aligned} \begin{aligned} \dot{\varvec{x}} = \begin{pmatrix} \dot{\varvec{r}} \\ \dot{\varvec{v}} \end{pmatrix}&= \begin{pmatrix} \varvec{v} \\ \varvec{M} \left( \varvec{F}_\textrm{rad}+\varvec{F}_\textrm{d} + \varvec{F}_\textrm{g} \right) \end{pmatrix} \\ \varvec{y}&= \varvec{r}, \end{aligned}, \end{aligned}$$

where the state vector $\varvec{x} \in {\mathbb {R}}^{6 \times 1}$ of the model is comprised of the position $\varvec{r} = r_\textrm{x} \varvec{e}_\textrm{x} + r_\textrm{y} \varvec{e}_\textrm{y} + r_\textrm{z} \varvec{e}_\textrm{z}$ and the velocity $\varvec{v} = v_\textrm{x} \varvec{e}_\textrm{x} + v_\textrm{y} \varvec{e}_\textrm{y} + v_\textrm{z} \varvec{e}_\textrm{z}$ of the sphere. The time-dependency of the state $\varvec{x}(t)$, input $\varvec{u}(t)$, and output $\varvec{y}(t)$ are omitted for brevity. In addition, we assumed that $\varvec{r}$, taken as output $\varvec{y}$ of the system, is measurable. In Eq. (33), $\varvec{F}_\textrm{g} = m\varvec{g} = -\frac{4}{3}\pi \rho _\textrm{P}a^3g_\textrm{z}\varvec{e}_\textrm{z}$ denotes the gravitational force, where *a* and $\rho$ are the radius and the density of the sphere, respectively. Furthermore, $\varvec{F}_\textrm{rad} \in {\mathbb {R}}^{3 \times 1}$ denotes the acoustic radiation force to be exerted on the sphere at its current position $\varvec{r}$. Instead of employing common approaches like refs.^15–17^ to calculate $\varvec{F}_\textrm{rad}(\varvec{r},\varvec{\phi })$, we solely used the polynomial model $\varvec{P}\left( \Delta \varvec{r}, \varvec{A} \right)$ to predict $\varvec{F}_\textrm{res} = \varvec{F}_\textrm{rad} + \varvec{F}_\textrm{g}$ for a displacement $\Delta \varvec{r} = \varvec{r} - \varvec{r}_\textrm{trap}$ between the sphere and the centre of the trap at $\varvec{r}_\textrm{trap}$, see sections Polynomial Regression and Training and validation of the polynomial models. Similar to our previous work^16^, we adapted the formula used by Fushimi et al.^47^ to calculate the drag force $\varvec{F}_\textrm{d} \in {\mathbb {R}}^{3 \times 1}$ by the formula

34 $$\begin{aligned} \varvec{F}_\textrm{d} = {\left\{ \begin{array}{ll} -\frac{1}{2} C_\textrm{d} \pi a^2 \rho _0 \left\| \varvec{v} \right\| _2 \varvec{v}, \quad \textrm{Re} > 0, \\ \phantom {-\frac{1}{2} C_\textrm{d} \pi a^2}\varvec{0}, \phantom {\left\| \varvec{v} \right\| _2 \varvec{v}} \quad \ \; \textrm{Re} = 0. \end{array}\right. } \end{aligned}$$

In Eq. (34), $\rho _0$ denotes the density of the fluid, $\left\| \varvec{v} \right\| _2 = \sqrt{v_\textrm{x}^2 + v_\textrm{y}^2 + v_\textrm{z}^2}$, and the Reynolds number $\textrm{Re}$ is calculated by

35 $$\begin{aligned} \textrm{Re} = \frac{2a \left\| \varvec{v} \right\| _2 \rho _0}{\eta }, \end{aligned}$$

where $\eta$ denotes the dynamic viscosity of the fluid^47^. In contrast to our previous work^16^, we used a more complex model for the drag coefficient $C_\textrm{d}$ from refs.^112,113^, namely

36 $$\begin{aligned} C_\textrm{d} = \frac{24}{\textrm{Re}} + \frac{2.6 \left( \frac{\textrm{Re}}{5.0}\right) }{1+\left( \frac{\textrm{Re}}{5.0}\right) ^{1.52}} + \frac{0.411 \left( \frac{\textrm{Re}}{2.63 \times 10^5}\right) ^{-7.94}}{1 + \left( \frac{\textrm{Re}}{2.63 \times 10^5}\right) ^{-8.00}} + \frac{0.25 \left( \frac{\textrm{Re}}{10^6}\right) }{1+\left( \frac{\textrm{Re}}{10^6}\right) }. \end{aligned}$$

In addition, without further evidence, the added mass $m_\textrm{I} = \frac{4}{3} \pi a^3 \rho _0$ of the sphere and history force $\varvec{F}_\textrm{h} \in {\mathbb {R}}^{3 \times 1}$ must be taken into account to reproduce the dynamic behaviour of the Mie sphere in the experiment adequately. Consequently, for the state space model in Eq. (33), this results in a matrix

37 $$\begin{aligned} \varvec{M} = \begin{pmatrix} \frac{1}{m + m_\textrm{I} - m_{\textrm{h,x}}} & 0 & 0 \\ 0 & \frac{1}{m + m_\textrm{I} - m_{\textrm{h,y}}} & 0 \\ 0 & 0 & \frac{1}{m + m_\textrm{I} - m_{\textrm{h,z}}} \\ \end{pmatrix}, \end{aligned}$$

where we use the formula for $\varvec{F}_\textrm{h}$ given in Eq. (13) in the work of Pantaleone and Messer^114^ and chose $B = 0.5 \tau C_\textrm{d} \rho _0 \pi a^2$ with $\tau \approx 0.3\,\textrm{s}$. This yields

38 $$\begin{aligned} m_{\textrm{h},i} = 0.5 \tau C_\textrm{d} \rho _0 \pi a^2 v_i, \quad i \in \left\{ \textrm{x},\textrm{y},\textrm{z}\right\} . \end{aligned}$$

### Trajectory planning by model predictive control

To improve the contactless manipulation of levitated objects, it is reasonable to use model-based trajectory planning. Similar to our previous work for the one-dimensional case^115^, this control problem can be formulated as the determination of a feasible trajectory $\varvec{u}(t) \in {\mathbb {R}}^{3 \times 1}$ for the centre position $\varvec{r}_\textrm{trap}(t)$ of the acoustic trap such that the position $\varvec{r}(t)$ of the sphere, taken as measurable output $\varvec{y}(t)$ of the system (see Eq. (33)), follows a given reference trajectory $\varvec{w}(t)$. The optimisation problem can then be stated as minimising the control error $e(t) = \left\| \varvec{w}(t)-\varvec{y}(t)\right\| _2$. Considering the constraints of the non-linear system, we first had to ensure that the prediction $\widehat{\varvec{F}}_\textrm{res} = \widehat{\varvec{F}}_\textrm{rad} + \varvec{F}_\textrm{g}$ of the polynomial model $\varvec{P}\left( \Delta \varvec{r}, \varvec{A} \right)$ for a given displacement $\Delta \varvec{r} = \varvec{r} - \varvec{r}_\textrm{trap}$ was feasible. Since we limited these predictions to a corresponding cubic volume $\mathcal {V}\left( \varvec{r}_\textrm{trap},R_\lambda \right)$ centred at $\varvec{r}_\textrm{trap}$ with edge length $2R_\lambda$, see Eq. (29), we had to ensure that the acoustic trap was always kept sufficiently close to the levitated object, which results in the following constraints:

39 $$\begin{aligned} \vert u_i(t) - x_i(t) \vert - R_\lambda \le 0, \quad i = \left\{ 1,2,3\right\} . \end{aligned}$$

In addition, the input $\varvec{u}(t)$ of the system was restricted by the serial communication interface between the PC and the FPGA. For the experimental setup in Augsburg, $\varvec{u}(t)$ could only be changed every $T_\textrm{c} = 2.82\,\textrm{ms}$ and remained constant between two consecutive activation updates. This constraint can be interpreted as a zero-order hold circuit with a sampling frequency of $f_\textrm{c}=1/T_\textrm{c}$ which converts the input $\varvec{u}(t)$ into a piecewise-constant signal:

40 $$\begin{aligned} \varvec{u}(t) = \varvec{u}[n], \quad n = \lfloor t/T_\textrm{c} \rfloor . \end{aligned}$$

To simplify matters, we further assumed that after an update of the activation $\varvec{\phi }$ the resulting radiation force $\varvec{F}_\textrm{rad}(\varvec{r},\varvec{\phi })$ is immediately exerted on the surface of the object placed at $\varvec{r}$. The transient oscillations of the transducers during a phase change and the subsequent propagation of the emitted wave fronts were neglected by this assumption. We assumed that the resulting error in each time interval $[nT_c,(n+1) T_c )$ is quite small and thus negligible in practical experiments due to the high influence of unknown disturbances. Moreover, to the best of the authors’ knowledge, it is currently unclear how the transient behaviour of $\varvec{F}_\textrm{rad}$ during a switch of the control variables of the PAT can be modelled with low computational costs and sufficient accuracy. Additionally, the PAT cannot generate $\varvec{\phi }$ with arbitrary resolution. For the PAT in Augsburg, this limit is $\pi /64\,\textrm{rad}$, causing a slight mismatch of the radiation force due to quantization errors of up to $\pi /128\,\textrm{rad}$. As it can be seen from Fig. (2b) in the supplementary material of the work of Andersson^17^, force magnitude errors of approximately up to $1\%$ can be expected for an EPS sphere of $8.5\,\textrm{mm}$ in diameter when each phase angle is randomly perturbed up to $2^\circ$. Although this error can be considered as significant, we decided to neglect its influence in our application, mainly to avoid the formulation of highly complicated mixed-integer problems.

Using the non-linear model in Eq. (33) and the constraints on $\varvec{u}(t)$ described above, we formulated an optimal control problem (OCP)^48^ for a given reference $\varvec{w}(t)$ with $t \in [0,T]$ and $T \gg T_\textrm{c}$, where $\xi \in {\mathbb {R}}^{+}$ is a positive hyper-parameter, see Eq. (41).

41 $$\begin{aligned} \begin{aligned}&\qquad \min \limits _{\varvec{u}(t)} {\xi \int _{0}^{T} \left\| \varvec{w}(t)-\varvec{y}(t)\right\| _2^2\,\,\textrm{d}t} \\ & \qquad \text { s.t.} \qquad \quad\dot{\varvec{x}}(t) = \begin{pmatrix} \varvec{v}(t) \\ \varvec{M} \left( \varvec{P}\left( \varvec{r}(t) - \varvec{u}(t),\varvec{A}\right) + \varvec{F}_\textrm{d}\right) \end{pmatrix}, \ \varvec{x}(0) = \varvec{x}_0, \ \\&\phantom {\vert u_i(t) - x_i(t) \vert } \ \ \varvec{y}(t) = \varvec{r}(t), \qquad\qquad \qquad \qquad \quad \quad \qquad \ \ \varvec{x}(T)= \varvec{x}_\textrm{T}, \\&\phantom {\vert u_i(t) - x_i(t) \vert } \ \ \varvec{u}(t) = \varvec{u}[n], \ nT_\textrm{c}\,\le \,t < (n+1) T_\textrm{c}, \ n \in {\mathbb {Z}}, \\&\vert u_i(t) - x_i(t) \vert - R_\lambda \le 0, \ i = \left\{ 1,2,3\right\} , \ R_\lambda \in {\mathbb {R}}^{+}, \ \ \, n \in [\,0,\lfloor T/T_\textrm{c} \rfloor \,]. \end{aligned} \end{aligned}$$

Furthermore, $\varvec{x}_0$ and $\varvec{x}_\textrm{T}$ denote the initial and the terminal state of the dynamic system in Eq. (33) in the interval $t \in [0,T]$, $T \in {\mathbb {R}}^{+}$. To solve the OCP, we decided to employ the model predictive control (MPC) toolbox in MATLAB. As MPC^99^ is mainly an optimal control strategy for discrete systems, the continuous OCP stated in Eq. (41) was discretised by the MPC toolbox using an implicit trapezoidal rule with a specified sample time of $T_\textrm{s} = T_\textrm{c}/100$. Furthermore, we chose $T_\textrm{c}$ as sample time of the controller and a zeroth-order interpolation scheme in each time interval $[nT_\textrm{c},(n+1) T_\textrm{c} )$ between two consecutive steps to reflect the restriction to the input $\varvec{u}(t)$ caused by the serial communication interface. Subsequently, we selected $\delta =500$ and $T_\textrm{p} = 10 T_\textrm{c}$ as prediction horizon and let the MPC toolbox determine a feasible $\varvec{u}^{*}(t)$ for the OCP. Finally, we employed Algorithm 3 with $L = 1$ and $i_\textrm{max} = 512$ from component ${\textcircled {{2}}}$ ($\Delta \varvec{r} \rightarrow \varvec{\phi }^{*}$) of the inverse model (see Fig. 2) to obtain a valid sequence of activations $\varvec{\phi }^{*}(t)$ from the trajectory $\varvec{u}^{*}(t)$ of the trap.

### Experimental setup

The experiments for this study were performed partially in São Paulo and partially in Augsburg. For the experiments in São Paulo, which are shown in Fig. 3(b,c), we used the *Sonic Surface*^98^, a $16\times16$ phased array that is equipped with $40\,\textrm{kHz}$ ultrasonic transducers (Manorshi MSO-P1040H07T) with a diameter of $9.8\,\textrm{mm}$ that are driven by square wave signals generated by a Field-programmable gate array (FPGA) (Altera Cyclone IV-EP4CE6). We used $8\,\textrm{bit}$ shift registers (Texas Instruments 74HC595) to convert the 32 outputs of the FPGA into 256 independent signals, which are amplified to up to $20\,\textrm{Vpp}$ by 128 dual low-side MOSFET gate drivers (Microchip MIC4127). Although square wave signals are used to drive the transducers, the emitted acoustic waves are sinusoidal due to the narrow bandwidth of the transducers^116^. To control their phase with a resolution of $\pi /16\,\textrm{rad}$, commands from MATLAB were transferred to the FPGA via a serial interface using a data transfer rate of $250\,\textrm{kbit}\,\textrm{s}^{-1}$. For the calibration of the phased array, we used a calibrated microphone (Brüel  & Kjaer, type 4138-A-015), which was moved by a 3 - axis XYZ translation stage (NRT150/M Thorlabs stages and BSC203 Thorlabs motor controller) to measure the emitted ultrasound waves (amplitudes and phases) of all transducers. These signals were amplified by a conditioning amplifier (Brüel  & Kjaer, Nexus 2690-A-0S2) and captured by an oscilloscope (Keysight DSOX2014A), which communicated with the PC via USB. During levitation experiments, the motion of the levitated objects was recorded with a high-speed camera (Photron FASTCAM Mini UX50) with a spatial resolution of $28\,\textrm{pixels}\,\textrm{mm}^{-1}$ and a frame rate of $500\,\textrm{fps}$. Subsequently, a tracking algorithm was used to extract the objects position from the videos.

The experimental setup in Augsburg was similar, in that it used the *Sonic Surface*^98^ and the same electronics for generating the driving signal for the ultrasonic transducers. A difference were the transducers used to generate the signals (Murata MA40S4S), which have the same dimensions and resonance frequency. Additionally, fans were mounted below the array to cool the MOSFET gate drivers and stabilise the operating conditions for the PAT, strongly facilitating the long-term stable trapping of levitated objects. Serial communication from MATLAB to the FPGA was set up with $922\,\textrm{kbit}\,\textrm{s}^{-1}$ and transferred phases with a resolution of $\pi /64\,\textrm{rad}$. For the experiments shown in Figs. 8 and 9, a *twin tuning forks trap*^16^ was created using an amplitude of $16\,\textrm{Vpp}$ for all transducers. The polystyrene sphere used in these experiments had a mass of $7.4\,\textrm{mg}$ and a diameter of $8.5\,\textrm{mm}$. The trajectories were filmed using a Photron FASTCAM Nova 6 with a spatial resolution of $20\,\textrm{pixels}\,\textrm{mm}^{-1}$ and a frame rate of $500\,\textrm{fps}$. The sphere positions were extracted using the same tracking algorithm as above and the motion accuracy was determined between the given reference trajectory and recorded positions.

## Supplementary Information

Supplementary Information 1.

Supplementary Information 2.

Supplementary Information 3.

Supplementary Information 4.

Supplementary Information 5.

Supplementary Information 6.

Supplementary Information 7.

## Data Availability

The data that support the findings of this study are provided within the article or supplementary information files and are available from the corresponding authors upon reasonable request.

## References

[1] Bruus, H. Acoustofluidics 7: The acoustic radiation force on small particles. *Lab on a chip* **12**, 1014–1021 (2012). 22349937 10.1039/c2lc21068a

[2] Andrade, M. A. B., Camargo, T. S. A. & Marzo, A. Automatic contactless injection, transportation, merging, and ejection of droplets with a multifocal point acoustic levitator. *The Review of scientific instruments* **89**, 125105 (2018). 30599572 10.1063/1.5063715

[3] Morris, R. H., Dye, E. R., Docker, P. & Newton, M. I. Beyond the Langevin horn: Transducer arrays for the acoustic levitation of liquid drops. *Physics of Fluids* **31**, 101301 (2019).

[4] Zang, D. (ed.) *Acoustic Levitation: From Physics to Applications* (Springer Singapore and Springer, Singapore, 2020), 1st ed. 2020 edn.

[5] Brandt, E. H. Levitation in physics. *Science (New York, N.Y.)* **243**, 349–355 (1989). 10.1126/science.243.4889.349 17787252

[6] Yarin, A. L., Pfaffenlehner, M. & Tropea, C. On the acoustic levitation of droplets. *Journal of Fluid Mechanics* **356**, 65–91 (1998).

[7] Xie, W. J. & Wei, B. Dynamics of acoustically levitated disk samples. *Physical review. E, Statistical, nonlinear, and soft matter physics* **70**, 046611 (2004). 15600551 10.1103/PhysRevE.70.046611

[8] Xie, W. J., Cao, C. D., Lü, Y. J., Hong, Z. Y. & Wei, B. Acoustic method for levitation of small living animals. *Applied Physics Letters* **89**, 214102 (2006).

[9] Zang, D. et al. Acoustic levitation of soap bubbles in air: Beyond the half-wavelength limit of sound. *Applied Physics Letters* **110**, 121602 (2017).

[10] Zang, D. et al. Acoustic levitation of liquid drops: Dynamics, manipulation and phase transitions. *Advances in colloid and interface science* **243**, 77–85 (2017). 28343560 10.1016/j.cis.2017.03.003

[11] Andrade, M. A. B. & Marzo, A. Numerical and experimental investigation of the stability of a drop in a single-axis acoustic levitator. *Physics of Fluids* **31**, 117101 (2019).

[12] Zhang, B. W., Hong, Z. Y. & Drinkwater, B. W. Transfer of orbital angular momentum to freely levitated high-density objects in airborne acoustic vortices. *Physical Review Applied* **18** (2022).

[13] Brandt, E. H. Acoustic physics. suspended by sound. *Nature* **413**, 474–475 (2001). 11586343 10.1038/35097192

[14] Marzo, A. et al. Holographic acoustic elements for manipulation of levitated objects. *Nature communications* **6**, 8661 (2015). 26505138 10.1038/ncomms9661 PMC4627579

[15] Inoue, S. et al. Acoustical boundary hologram for macroscopic rigid-body levitation. *The Journal of the Acoustical Society of America* **145**, 328 (2019). 30710964 10.1121/1.5087130

[16] Zehnter, S., Andrade, M. A. B. & Ament, C. Acoustic levitation of a Mie sphere using a 2D transducer array. *Journal of Applied Physics* **129**, 134901 (2021).

[17] Andersson, C. Acoustic levitation of multi-wavelength spherical bodies using transducer arrays of non-specialized geometries. *The Journal of the Acoustical Society of America* **151**, 2999 (2022). 35649903 10.1121/10.0010358

[18] Ueha, S., Hashimoto, Y. & Koike, Y. Non-contact transportation using near-field acoustic levitation. *Ultrasonics* **38**, 26–32 (2000). 10829622 10.1016/s0041-624x(99)00052-9

[19] Courtney, C. R. P. et al. Dexterous manipulation of microparticles using Bessel-function acoustic pressure fields. *Applied Physics Letters* **102**, 123508 (2013).

[20] Kang, S.-T. & Yeh, C.-K. Potential-well model in acoustic tweezers. *IEEE transactions on ultrasonics, ferroelectrics, and frequency control* **57**, 1451–1459 (2010). 20529720 10.1109/TUFFC.2010.1564

[21] Ghanem, M. A. et al. Noninvasive acoustic manipulation of objects in a living body. *Proceedings of the National Academy of Sciences of the United States of America* **117**, 16848–16855 (2020). 32631991 10.1073/pnas.2001779117 PMC7382215

[22] Helander, P. et al. Omnidirectional microscopy by ultrasonic sample control. *Applied Physics Letters* **116**, 194101 (2020).

[23] Tsujino, S. & Tomizaki, T. Ultrasonic acoustic levitation for fast frame rate X-ray protein crystallography at room temperature. *Scientific reports* **6**, 25558 (2016). 27150272 10.1038/srep25558 PMC4858681

[24] Contreras, V. et al. Chemical elemental analysis of single acoustic-levitated water droplets by laser-induced breakdown spectroscopy. *Optics letters* **43**, 2260–2263 (2018). 29762567 10.1364/OL.43.002260

[25] Barbosa, E. J. et al. Acoustic levitation and high-resolution synchrotron x-ray powder diffraction: A fast screening approach of niclosamide amorphous solid dispersions. *International journal of pharmaceutics* **602**, 120611 (2021). 33872710 10.1016/j.ijpharm.2021.120611

[26] Andrade, M. A. B., Ramos, T. S., Adamowski, J. C. & Marzo, A. Contactless pick-and-place of millimetric objects using inverted near-field acoustic levitation. *Applied Physics Letters* **116**, 054104 (2020).

[27] Foresti, D., Nabavi, M., Klingauf, M., Ferrari, A. & Poulikakos, D. Acoustophoretic contactless transport and handling of matter in air. *Proceedings of the National Academy of Sciences of the United States of America* **110**, 12549–12554 (2013). 23858454 10.1073/pnas.1301860110 PMC3732964

[28] Vasileiou, T., Foresti, D., Bayram, A., Poulikakos, D. & Ferrari, A. Toward contactless biology: Acoustophoretic DNA transfection. *Scientific reports* **6**, 20023 (2016). 26828312 10.1038/srep20023 PMC4734324

[29] Andrade, M. A. B., Bernassau, A. L. & Adamowski, J. C. Acoustic levitation of a large solid sphere. *Applied Physics Letters* **109**, 044101 (2016).

[30] Marzo, A. & Drinkwater, B. W. Holographic acoustic tweezers. *Proceedings of the National Academy of Sciences of the United States of America* **116**, 84–89 (2019). 30559177 10.1073/pnas.1813047115 PMC6320506

[31] Röthlisberger, M., Schmidli, G., Schuck, M. & Kolar, J. W. Multi-frequency acoustic levitation and trapping of particles in all degrees of freedom. *IEEE transactions on ultrasonics, ferroelectrics, and frequency control* **69**, 1572–1575 (2022). 35130156 10.1109/TUFFC.2022.3149302

[32] Ochiai, Y., Hoshi, T. & Rekimoto, J. Three-dimensional mid-air acoustic manipulation by ultrasonic phased arrays. *PloS one* **9**, e97590 (2014). 24849371 10.1371/journal.pone.0097590 PMC4029622

[33] Hoshi, T., Ochiai, Y. & Rekimoto, J. Three-dimensional noncontact manipulation by opposite ultrasonic phased arrays. *Japanese Journal of Applied Physics* **53**, 07KE07 (2014).

[34] Plasencia, D. M., Hirayama, R., Montano-Murillo, R. & Subramanian, S. GS-PAT: high-speed multi-point sound-fields for phased arrays of transducers. *ACM Transactions on Graphics* **39** (2020).

[35] Puranen, T. *et al.* Multifrequency acoustic levitation. In *2019 IEEE International Ultrasonics Symposium (IUS)*, 916–919 (IEEE, 2019).

[36] Melde, K., Mark, A. G., Qiu, T. & Fischer, P. Holograms for acoustics. *Nature* **537**, 518–522 (2016). 27652563 10.1038/nature19755

[37] Melde, K. et al. Compact holographic sound fields enable rapid one-step assembly of matter in 3D. *Science advances* **9**, eadf6182 (2023). 36753553 10.1126/sciadv.adf6182 PMC9908023

[38] Hirayama, R., Martinez Plasencia, D., Masuda, N. & Subramanian, S. A volumetric display for visual, tactile and audio presentation using acoustic trapping. *Nature* **575**, 320–323 (2019). 31723288 10.1038/s41586-019-1739-5

[39] Ochiai, Y., Hoshi, T. & Rekimoto, J. Pixie dust. *ACM Transactions on Graphics* **33**, 1–13 (2014).

[40] Omirou, T., Marzo, A., Seah, S. A. & Subramanian, S. LeviPath. In Begole, B., Kim, J., Inkpen, K. & Woo, W. (eds.) *Proceedings of the 33rd Annual ACM Conference on Human Factors in Computing Systems*, 309–312 (ACM, New York, NY, USA, 2015).

[41] Korhonen, M. *et al.* Simultaneous orientation locking and translation of samples with phased arrays. In *2022 IEEE International Ultrasonics Symposium (IUS)*, 1–3 (IEEE, 2022).

[42] Long, B., Seah, S. A., Carter, T. & Subramanian, S. Rendering volumetric haptic shapes in mid-air using ultrasound. *ACM Transactions on Graphics* **33**, 1–10 (2014).

[43] Marzo, A., Caleap, M. & Drinkwater, B. W. Acoustic virtual vortices with tunable orbital angular momentum for trapping of Mie particles. *Physical review letters* **120**, 044301 (2018). 29437423 10.1103/PhysRevLett.120.044301

[44] Andrade, M. A. B., Pérez, N. & Adamowski, J. C. Experimental study of the oscillation of spheres in an acoustic levitator. *The Journal of the Acoustical Society of America* **136**, 1518–1529 (2014). 25324056 10.1121/1.4893905

[45] Pérez, N., Andrade, M. A. B., Canetti, R. & Adamowski, J. C. Experimental determination of the dynamics of an acoustically levitated sphere. *Journal of Applied Physics* **116**, 184903 (2014).

[46] Hasegawa, K. & Kono, K. Oscillation characteristics of levitated sample in resonant acoustic field. *AIP Advances* **9**, 035313 (2019).

[47] Fushimi, T., Hill, T. L., Marzo, A. & Drinkwater, B. W. Nonlinear trapping stiffness of mid-air single-axis acoustic levitators. *Applied Physics Letters* **113**, 034102 (2018).

[48] Paneva, V., Fleig, A., Plasencia, D. M., Faulwasser, T. & Müller, J. OptiTrap: Optimal trap trajectories for acoustic levitation displays. *ACM Transactions on Graphics* **41**, 1–14 (2022).

[49] Fliess, M., Lévine, J., Martin, P. & Rouchon, P. Flatness and defect of non-linear systems: introductory theory and examples. *International journal of control* **61**, 1327–1361 (1995).

[50] Matouš, J., Kollarčík, A., Gurtner, M., Michálek, T. & Hurák, Z. Optimization-based feedback manipulation through an array of ultrasonic transducers. *IFAC-PapersOnLine* **52**, 483–488 (2019).

[51] Kasai, T., Furumoto, T. & Shinoda, H. Rotation and position control of a cubic object using airborne ultrasound. In *2020 IEEE International Ultrasonics Symposium (IUS)*, 1–4 (IEEE, 2020).

[52] Gurtner, M., Zemánek, J. & Hurák, Z. Alternating direction method of multipliers-based distributed control for distributed manipulation by shaping physical force fields. *The International Journal of Robotics Research* 027836492311539 (2023).

[53] Hasegawa, K., Abe, Y., Kaneko, A., Yamamoto, Y. & Aoki, K. Visualization measurement of streaming flows associated with a single-acoustic levitator. *Microgravity Science and Technology* **21**, 9–14 (2009).

[54] Hasegawa, K., Qiu, L., Noda, A., Inoue, S. & Shinoda, H. Electronically steerable ultrasound-driven long narrow air stream. *Applied Physics Letters* **111**, 064104 (2017).

[55] Busse, F. H. & Wang, T. G. Torque generated by orthogonal acoustic waves–theory. *The Journal of the Acoustical Society of America* **69**, 1634–1638 (1981).

[56] Andrade, M. A. B., Ramos, T. S., Okina, F. T. A. & Adamowski, J. C. Nonlinear characterization of a single-axis acoustic levitator. *Review of Scientific Instruments* **85**, 045125 (2014). 24784677 10.1063/1.4872356

[57] Waldspurger, I., d’Aspremont, A. & Mallat, S. Phase recovery, maxcut and complex semidefinite programming. *Mathematical Programming* **149**, 47–81 (2015).

[58] Vandenberghe, L. & Boyd, S. Semidefinite programming. *SIAM Review* **38**, 49–95 (1996).

[59] Wang, P.-W., Chang, W.-C. & Kolter, J. Z. The mixing method: low-rank coordinate descent for semidefinite programming with diagonal constraints. *arXiv preprint*arXiv:1706.00476 (2017).

[60] Burer, S. & Monteiro, R. D. A nonlinear programming algorithm for solving semidefinite programs via low-rank factorization. *Mathematical Programming* **95**, 329–357 (2003).

[61] Burer, S. & Monteiro, R. D. Local minima and convergence in low-rank semidefinite programming. *Mathematical Programming* **103**, 427–444 (2005).

[62] Erdogdu, M. A., Ozdaglar, A., Parrilo, P. A. & Vanli, N. D. Convergence rate of block-coordinate maximization Burer-Monteiro method for solving large sdps. *Mathematical Programming* **195**, 243–281 (2022).

[63] Gor’kov, L. P. On the forces acting on a small particle in an acoustical field in an ideal fluid. *Sov. Phys.-Doklady* **6**, 773–775 (1962).

[64] Sapozhnikov, O. A. & Bailey, M. R. Radiation force of an arbitrary acoustic beam on an elastic sphere in a fluid. *The Journal of the Acoustical Society of America* **133**, 661–676 (2013). 23363086 10.1121/1.4773924 PMC3574112

[65] Abdelaziz, M. A. & Grier, D. G. Acoustokinetics: Crafting force landscapes from sound waves. *Physical Review Research* **2** (2020).

[66] Röthlisberger, M., Schuck, M., Kulmer, L. & Kolar, J. W. Contactless picking of objects using an acoustic gripper. *Actuators* **10**, 70 (2021).

[67] Ezcurdia, I., Morales, R., Andrade, M. A. B. & Marzo, A. LeviPrint: Contactless fabrication using full acoustic trapping of elongated parts. In *Special Interest Group on Computer Graphics and Interactive Techniques Conference Proceedings, 1–9 (ACM* (eds Nandigjav, M. et al.) (NY, USA, New York, 2022).

[68] Bachynskyi, M., Paneva, V. & Müller, J. LeviCursor. In Koike, H. *et al.* (eds.) *Proceedings of the 2018 ACM International Conference on Interactive Surfaces and Spaces*, 253–262 (ACM, New York, NY, USA, 2018).

[69] Gerchberg, R. W. A practical algorithm for the determination of plane from image and diffraction pictures. *Optik* **35**, 237–246 (1972).

[70] Mellin, S. & Nordin, G. Limits of scalar diffraction theory and an iterative angular spectrum algorithm for finite aperture diffractive optical element design. *Optics express* **8**, 705–722 (2001). 19421262 10.1364/oe.8.000705

[71] Fushimi, T., Yamamoto, K. & Ochiai, Y. Target acoustic field and transducer state optimization using diff-pat. *AIP Advances* **11**, 125007 (2021).

[72] Fushimi, T., Yamamoto, K. & Ochiai, Y. Acoustic hologram optimisation using automatic differentiation. *Scientific reports* **11**, 12678 (2021). 34135364 10.1038/s41598-021-91880-2 PMC8209099

[73] Chen, J. *et al.* Sound pressure field reconstruction for ultrasound phased array by linear synthesis scheme optimization. In Seifi, H. *et al.* (eds.) *Haptics: Science, Technology, Applications*, vol. 13235 of *Lecture Notes in Computer Science*, 147–154 (Springer International Publishing, Cham, 2022).

[74] Suzuki, S., Fujiwara, M., Makino, Y. & Shinoda, H. Radiation pressure field reconstruction for ultrasound midair haptics by greedy algorithm with brute-force search. *IEEE transactions on haptics* **14**, 914–921 (2021). 33914686 10.1109/TOH.2021.3076489

[75] Chen, J. *et al.* Sound pressure field reconstruction for airborne ultrasound tactile display encountering obstacles. *IEEE transactions on haptics* **PP** (2023). 10.1109/TOH.2023.3309975 37647186

[76] Iablonskyi, D. *et al.* Tailored acoustic holograms with phased arrays. In *2022 IEEE International Ultrasonics Symposium (IUS)*, 1–3 (IEEE, 2022).

[77] Levenberg, K. A method for the solution of certain non-linear problems in least squares. *Quarterly of Applied Mathematics* **2**, 164–168 (1944).

[78] Marquardt, D. W. An algorithm for least-squares estimation of nonlinear parameters. *Journal of the Society for Industrial and Applied Mathematics* **11**, 431–441 (1963).

[79] Matsubayashi, A., Makino, Y. & Shinoda, H. Accurate control of sound field amplitude for ultrasound haptic rendering using the Levenberg–Marquardt method. In *2022 IEEE Haptics Symposium (HAPTICS)*, 1–6 (IEEE, 2022).

[80] Li, B., Lu, M., Liu, C., Liu, X. & Ta, D. Acoustic hologram reconstruction with unsupervised neural network. *Frontiers in Materials* **9**, 916527 (2022).

[81] Wang, S., Wang, X., You, F. & Xiao, H. A deep learning based method for generating holographic acoustic fields from phased transducer arrays. In *International Conference on Artificial Neural Networks*, 13–24 (2023).

[82] Zhong, C., Sun, Z., Li, T., Su, H. & Liu, S. Real-time acoustic holography with iterative unsupervised learning for acoustic robotic manipulation. In *2023 IEEE International Conference on Robotics and Automation (ICRA)*, 5466–5472 (IEEE, 2023).

[83] Yang, L., You, F. & Wang, X. A survey on acoustic control systems. In Akhtar, N., Draman, A. K. & Abdollah, M. F. (eds.) *Proceedings of the 2023 3rd International Conference on Public Management and Intelligent Society (PMIS 2023)*, vol. 8 of *Atlantis Highlights in Intelligent Systems*, 926–937 (Atlantis Press International BV, Dordrecht, 2023).

[84] Inoue, S., Makino, Y. & Shinoda, H. Active touch perception produced by airborne ultrasonic haptic hologram. In *2015 IEEE World Haptics Conference (WHC)*, 362–367 (IEEE, 2015).

[85] Majumdar, A., Hall, G. & Ahmadi, A. A. Recent scalability improvements for semidefinite programming with applications in machine learning, control, and robotics. *Annual Review of Control, Robotics, and Autonomous Systems* **3**, 331–360 (2020).

[86] Wang, P.-W. *Learning and Reasoning with Fast Semidefinite Programming and Mixing Methods*. Ph.D. thesis, Carnegie Mellon University (2021).

[87] Kim, J. L. *et al.* Momentum-inspired low-rank coordinate descent for diagonally constrained SDPs. *arXiv preprint* arXiv:2106.08775 (2021).

[88] Beentjes, C. H. L. Quadrature on a spherical surface. *Working note available on the website http://people. maths. ox. ac. uk/beentjes/Essays* (2015).

[89] Semechko Anton. Suite of functions to perform uniform sampling of a sphere (github) (2022). http://github.com/AntonSemechko/S2-Sampling-Toolbox.

[90] Lebedev, V. I. & Laikov, D. N. A quadrature formula for the sphere of the 131st algebraic order of accuracy. *In Doklady Mathematics* **59**, 477–481 (1999).

[91] Robert Parrish. getlebedevsphere (matlab central file exchange) (2022). http://www.mathworks.com/matlabcentral/fileexchange/27097-getlebedevsphere.

[92] Golub, G. H. & Welsch, J. H. Calculation of gauss quadrature rules. *Mathematics of computation* **23**, 221–230 (1969).

[93] von Winckel, G. Legendre-gauss quadrature weights and nodes (2004). https://de.mathworks.com/matlabcentral/fileexchange/4540-legendre-gauss-quadrature-weights-and-nodes.

[94] Becker, S. LBFGSB (L-BFGS-B) mex wrapper (2023). https://github.com/stephenbeckr/L-BFGS-B-C.

[95] Chen, Y. & Goulart, P. Burer-monteiro admm for large-scale diagonally constrained sdps. In *2022 European Control Conference (ECC)*, 66–71 (IEEE, 2022).

[96] Kronbichler, M. & Kormann, K. Fast matrix-free evaluation of discontinuous Galerkin finite element operators. *ACM Transactions on Mathematical Software* **45**(29), 1–40 (2019).

[97] van Zee, F. G. & Smith, T. M. Implementing high-performance complex matrix multiplication via the 3m and 4m methods. *ACM Transactions on Mathematical Software* **44**, 1–36 (2017).

[98] Morales, R., Ezcurdia, I., Irisarri, J., Andrade, M. A. B. & Marzo, A. Generating airborne ultrasonic amplitude patterns using an open hardware phased array. *Applied Sciences* **11**, 2981 (2021).

[99] Grüne, L. & Pannek, J. *Nonlinear model predictive control* (Springer, 2017).

[100] Koroyasu, Y. *et al.* Microfluidic platform using focused ultrasound passing through hydrophobic meshes with jump availability. *PNAS Nexus* **2**, pgad207 (2023). 10.1093/pnasnexus/pgad207 PMC10317206 37404834

[101] Zehnter, S. & Ament, C. A modular FPGA-based phased array system for ultrasonic levitation with MATLAB. In *2019 IEEE International Ultrasonics Symposium (IUS)*, 654–658 (IEEE, 2019).

[102] Luo, Z.-Q., Ma, W.-K., So, A.M.-C., Ye, Y. & Zhang, S. Semidefinite relaxation of quadratic optimization problems. *IEEE Signal Processing Magazine* **27**, 20–34 (2010).

[103] Eckart, C. & Young, G. The approximation of one matrix by another of lower rank. *Psychometrika* **1**, 211–218 (1936).

[104] Goemans, M. X. & Williamson, D. P. Improved approximation algorithms for maximum cut and satisfiability problems using semidefinite programming. *Journal of the ACM* **42**, 1115–1145 (1995).

[105] Boumal, N., Voroninski, V. & Bandeira, A. The non-convex Burer–Monteiro approach works on smooth semidefinite programs. *Advances in Neural Information Processing Systems* **29** (2016).

[106] Barvinok, A. I. Problems of distance geometry and convex properties of quadratic maps. *Discrete & Computational Geometry* **13**, 189–202 (1995).

[107] Pataki, G. On the rank of extreme matrices in semidefinite programs and the multiplicity of optimal eigenvalues. *Mathematics of Operations Research* **23**, 339–358 (1998).

[108] Journée, M., Bach, F., Absil, P.-A. & Sepulchre, R. Low-rank optimization on the cone of positive semidefinite matrices. *SIAM Journal on Optimization* **20**, 2327–2351 (2010).

[109] Coppersmith, D. & Winograd, S. Matrix multiplication via arithmetic progressions. *Journal of Symbolic Computation* **9**, 251–280 (1990).

[110] Le Gall, F. Faster algorithms for rectangular matrix multiplication. In *2012 IEEE 53rd Annual Symposium on Foundations of Computer Science*, 514–523 (IEEE, 2012).

[111] Goto, K. & de van Geijn, R. A. Anatomy of high-performance matrix multiplication. *ACM Transactions on Mathematical Software* **34**, 1–25 (2008).

[112] Morrison, F. *An introduction to fluid mechanics* (Cambridge University Press, 2013), 1st edn.

[113] Morrison, F. A. Data correlation for drag coefficient for sphere. *Department of Chemical Engineering, Michigan Technological University, Houghton, MI* **49931** (2013).

[114] Pantaleone, J. & Messer, J. The added mass of a spherical projectile. *American Journal of Physics* **79**, 1202–1210 (2011).

[115] Andrade, M. A. B., Zehnter, S., Funke, F. & Ament, C. Model-based feedforward control for an optimized manipulation of acoustically levitated spheres. *AIP Advances* **14** (2024).

[116] Marzo, A., Corkett, T. & Drinkwater, B. W. Ultraino: An open phased-array system for narrowband airborne ultrasound transmission. *IEEE transactions on ultrasonics, ferroelectrics, and frequency control* **65**, 102–111 (2018). 29283352 10.1109/TUFFC.2017.2769399

[117] Altman, Y. export_fig (github) (2022). http://github.com/altmany/export_fig/releases/tag/v3.28.

[118] Schlömer, N. matlab2tikz (github) (2022). http://github.com/matlab2tikz/matlab2tikz.

