# Supplementary material for: Semidefinite programming for manipulating acoustic traps in real time (SMART)
Source: Sci Rep. 2025 May 20;15:17523. doi: 10.1038/s41598-025-93153-8 (PMC12092699; doi:10.1038/s41598-025-93153-8)
Supplement: Supplementary file 1 — Supplementary Information 1. [file 41598_2025_93153_MOESM1_ESM.pdf]

Semidefinite Programming for Manipulating Acoustic Traps in Real Time (SMART)

Sebastian Zehnter[1], Kevin Endres[1], Martin Kronbichler[2], Marco A. B. Andrade[3], Felix Funke[1], and Christoph Ament[1]

Affiliations

[1] Chair of Control Engineering, University of Augsburg, Augsburg, 86159 Germany

[2] Chair of High-Performance Scientific Computing, University of Augsburg, Augsburg, 86159 Germany

[2] Faculty of Mathematics, Ruhr University Bochum, Bochum, 44801 Germany

[3] Institute of Physics, University of São Paulo, São Paulo 05508-090, Brazil

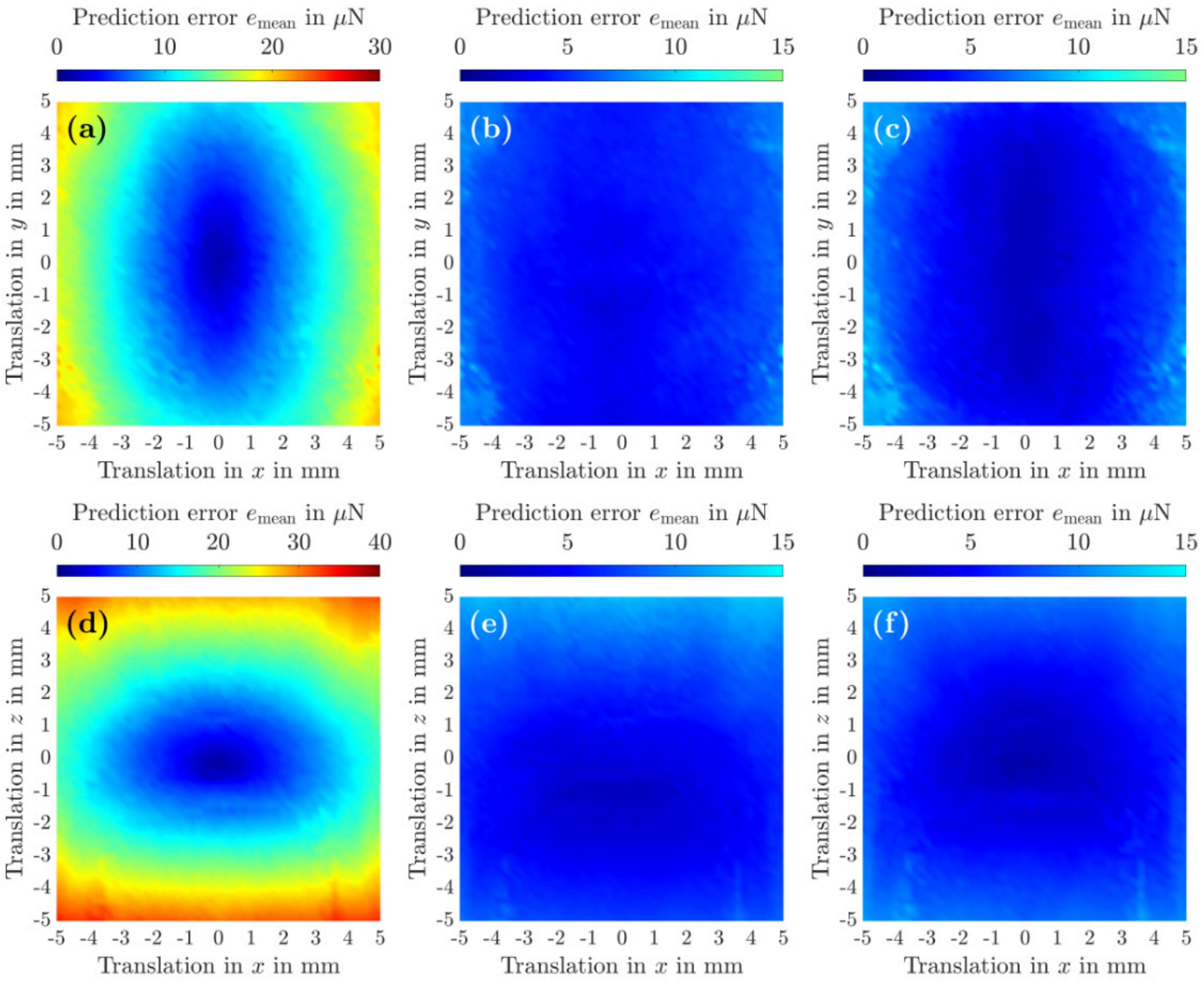

Figure S1: Comparison of the force distribution recovery accuracy of the holographic acoustic elements approach [(a),(d)] of Marzo et al. [14] described on p. 6. with Alg. 3 [(b),(c),(e),(f)]. In this simulative analysis, the settings from the experimental setup in Augsburg were used. Instead of 16 Vpp (see Fig. 7), we used here only 10 Vpp as amplitude for the transducers. Taking this modification into account, we created an appropriate *twin tunings forks trap* [16] defined by $\boldsymbol{\phi}_{\mathrm{opt}}$ to levitate a EPS sphere of $d = 8.5\,\mathrm{mm}$ in diameter and a mass of $m = 7.4\,\mathrm{mg}$ at $\boldsymbol{r}_{\mathrm{opt}} = (0\,\boldsymbol{e}_{\mathrm{x}} + 0\,\boldsymbol{e}_{\mathrm{y}} + 50\,\boldsymbol{e}_{\mathrm{z}})\,\mathrm{mm}$. From $\boldsymbol{r}_{\mathrm{opt}}$, the sound pressure field of the optimised trap was translated by $\Delta x, \Delta y \in [-5, 5]$ mm in the $xy-$plane $[\mathbf{(a)}, \mathbf{(b)}, \mathbf{(c)}]$ as well as by $\Delta x, \Delta z \in [-5, 5]$ mm in the $xz-$plane $[\mathbf{(d)}, \mathbf{(e)}, \mathbf{(f)}]$. To compare the accuracy of both algorithms, we employed Alg. 4 and the prediction error e_{mean} (see Eq. (32)) as metric. For the training of $\widehat{\boldsymbol{F}}_{\mathrm{res}} = \boldsymbol{P}(\Delta \boldsymbol{r}, \boldsymbol{A})$, we selected $\delta = 3$, $\boldsymbol{r}_{\mathcal{W}} = \boldsymbol{r}_{\mathrm{opt}}$, $R_{\Omega} = 6.25\,\mathrm{mm}$ for $\mathcal{W} = \mathcal{V}(\boldsymbol{r}_{\mathcal{W}}, R_{\Omega})$ and $R_{\lambda} = 1.25\,\mathrm{mm}$ for $\mathcal{B} = \mathcal{V}(\boldsymbol{r}_{\mathrm{opt}}, R_{\lambda}) \subset \mathcal{W}$, see Eq. (1). Sampling $\mathcal{B}$ and $\mathcal{V}(\mathbf{0}, R_{\lambda})$ in cubic grids with spacings of 0.125 mm and 0.25 mm respectively resulted in $I = 9261$ data tuples and $J = 1331$ relative displacements $\Delta \boldsymbol{r}_j \in \mathcal{V}(\mathbf{0}, R_{\lambda})$, $j = \{1, 2, \ldots, J\}$, where the local force distribution was evaluated. To calculate $\boldsymbol{F}_{\mathrm{rad}}$, we used the approach in [17]. To evaluate $\widehat{\boldsymbol{F}}_{\mathrm{res}} = \boldsymbol{P}(\Delta \boldsymbol{r}, \boldsymbol{A})$, we sampled $\mathcal{V}(\boldsymbol{r}_{\mathcal{W}}, R_{\Omega} - R_{\lambda}) \subset \mathcal{W}$ with a cubic grid with 0.2 mm spacing, resulting in $L = 2601$ test positions. In **(b)** and **(e)**, Alg. 3 was executed with $\boldsymbol{G} \in \mathbb{R}^{M \times N}$, whereas in **(c)** and **(f)**, Alg. 3 employed $\widehat{\boldsymbol{G}} \in \mathbb{R}^{4M \times N}$, $\widehat{\boldsymbol{G}} = \begin{pmatrix} \boldsymbol{G}^{\top} & \boldsymbol{G}_{\mathrm{x}}^{\top} & \boldsymbol{G}_{\mathrm{y}}^{\top} & \boldsymbol{G}_{\mathrm{z}}^{\top} \end{pmatrix}^{\top}$ (see Eqs. (4) – (7)). Finally, in **[(b),(c),(e),(f)]**, Alg. 3 was executed with $L = 1$ and $i_{\mathrm{max}} = 1024$.
